# Marine plastic pollution undermines the livelihoods and income of fishing communities in Viet Nam

**DOI:** 10.1038/s43247-026-03567-z

**Published:** 2026-06-08

**Authors:** Duc Nguyen, Phan Phuong Thanh, Heidi L. Burdett, Inna Yaneva-Toraman, Zhiling Liao, Qingping Zou, Trinh Quang Tu, Le Trung Dung, Le Thi Thu Huong, Ryan Pereira, Thomas Wagner, Huong Thi Thuy Ngo, Michel J. Kaiser

**Affiliations:** 1 The Lyell Centre, Heriot-Watt University, Edinburgh, UK; 2 Research Center of Fisheries Economics and Planning, Vietnam Academy of Fisheries Sciences, Hanoi, Viet Nam; 3 Umeå Marine Sciences Centre, Umeå University, Norrbyn, Sweden; 4 Department of Ecology, Environment and Geoscience, Umeå University, Umeå, Sweden; 5 Faculty of Biotechnology, Chemistry and Environmental Engineering, Phenikaa University, Hanoi, Viet Nam

**Keywords:** Environmental impact, Environmental studies

## Abstract

Globally, small-scale fishing communities are impacted by plastic debris accumulating in coastal waters. We studied the socioeconomic impact of marine plastic on fishers in the Mekong and Red River Deltas. Using a mixed methods design, we quantified the impacts of marine plastics through socioeconomic structured interviews with nearshore fishers (*n* = 199), post-hoc verification interviews (*n* = 94) and waste audits of debris in fishing nets (*n* = 282). The total cost of plastic pollution was calculated as ~3400 USD vessel^-1^ year^-1^, equivalent to 12% of the annual vessel revenue and 25% of the owner’s income. Fishers also experienced injuries and fatalities when dealing with plastic entanglement incidents at sea. Results highlighted a disproportionate and unequitable higher economic burden faced by lower-income fishers in the Mekong Delta compared to the Red River Delta. Our study found that incentive-based plastic collection at priority locations could reduce pollution while supporting sustainable livelihoods.

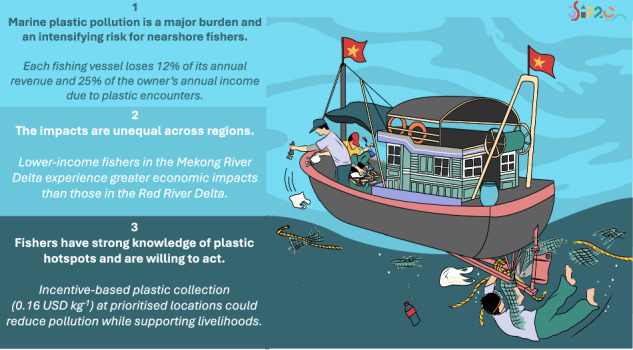

## Abstract (web summary)

Plastic pollution reduced annual revenue for fishers in Vietnam’s Mekong and Red River regions and caused safety risks, based on structured interviews, validation interviews and waste audits of plastics caught in fishing nets.

## Introduction

Plastic debris and microplastics have been detected in almost every marine environment, from Arctic ice^1^ to deep ocean trenches^2^, entangling and accumulating in wildlife^3,4^, and in human organs^5^. Numerous studies have identified the spatiotemporal magnitude of global marine plastic pollution, which encompasses plastic particles from metre to nanometre scales^6–8^. The majority of marine plastic debris entering oceans originates from major river systems in Asian countries^6,9^, especially during extreme hydrological events^10,11^, and becomes concentrated in coastal areas^12,13^. Even though plastic pollution is a global issue, the plastic waste trade^14^ and export to Asian countries with high coastal population densities and the associated plastic leakage rates render this region as the one that bears the highest price tag of plastic pollution, which is estimated to be 11 billion USD in 2018^15^. The lifetime socioeconomic cost of marine plastic pollution in 2019 was estimated at 3.7 trillion USD and is projected to exceed 7.1 trillion USD by 2040^16^. These costs were modelled based on quantifiable costs from greenhouse gases, waste management, and impacts of mismanaged waste on marine ecosystems. Globally, the direct economic impact of plastic pollution on tourism, fisheries, and governance along coastlines and waterways has been estimated at 2 USD per capita per year, with 82% attributed to cleanup costs and 18% to revenue loss^15^. Nevertheless, we currently lack an understanding of the socioeconomic impacts of plastic pollution on coastal communities; current knowledge is primarily informed through modelling that does not accurately quantify the unseen impacts on communities and their well-being^17,18^. Here, we address a key knowledge gap in existing macro-scale estimation models by providing empirically based costs of marine plastic debris on the incomes and livelihoods of fishing communities.

Fishing activities are estimated to contribute more than 10% of the plastic in the ocean^19^. Small-scale fisheries contribute over 40% of world catch and value, and play a vital role in nutrition and livelihoods, especially across Asia^20^. Viet Nam is one of the top five global exporters of fisheries products, valued at 10 billion USD in 2024 and fisheries are a significant contributor to national economic development, making up 4–5% of the national gross domestic product^21^. In 2023, 65% of Viet Nam’s 86,820 fishing vessels operated in the nearshore fishing zone^22^, mainly consisting of gillnetters and trawlers^22,23^. Despite the growing problem of plastic pollution along Viet Nam’s coastline^24^, there is limited understanding of the socioeconomic impact of marine plastic debris on coastal fishing communities^25^ and a lack of community voice on suitable interventions that might alleviate plastic pollution in a local context.

We used a combination of quantitative and qualitative methods to quantify the cost of plastic pollution from the perspective of the affected fishing communities at three major fishing areas in Viet Nam, located at the outlets of the Red River and Mekong River. This research provides important insights into the impacts of plastic pollution, which can help shape and implement realistic targets and policies at both local, national and international levels. It aims to advance marine plastics research by focusing on two key areas: (1) lack of data on the socioeconomic impacts of plastics on coastal communities, and (2) limitations of macroscopic economic models that reply on assumptions and overlook empirical data from impacted communities.

## Results and discussion

### Cost of marine plastic debris

This study used a mixed methods approach, including structured interviews with fishers (*n* = 199), focus group discussions (*n* = 3 with 8 participants each), semi-structured interviews with port managers (*n* = 6), and fisheries officers (*n* = 9) across three nearshore regions of Hai Phong, Nam Dinh, and Ben Tre, at the outlets of the Red and Mekong Rivers in Viet Nam. Reported data were analysed to estimate the economic and societal costs of marine plastic debris on small-scale nearshore fisheries. The socioeconomic impact assessment and fishers’ ecological knowledge of marine plastic pollution were further complemented by verification surveys with different fishers (*n* = 94) and waste audits of debris caught in fishing nets (*n* = 282) in the same geographic areas. The main findings are presented below; detailed provincial and gear-specific data are provided in the Supplementary Information.

**Fig. 1:**
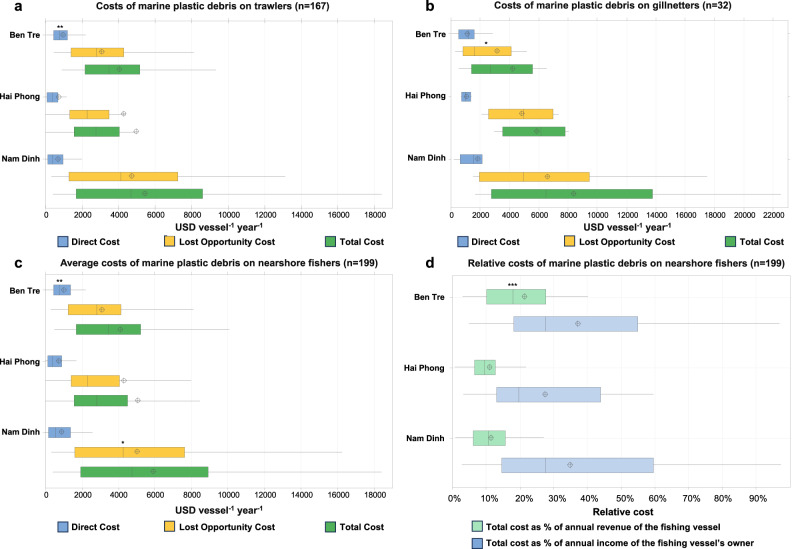
The economic cost of marine plastic debris on fishing vessels. Box plots of the direct costs, lost opportunity costs and total costs (USD vessel^−1^ year^−1^) associated with marine plastic debris that were calculated for trawlers (*n* = 167) (**a**), gillnetters (*n* = 32) (**b**), all gear types (*n* = 199) (**c**) across Nam Dinh, Hai Phong, and Ben Tre. (**d)** Box plots of relative total costs of all nearshore fishers (*n* = 199) expressed as percentages of annual revenue of vessel and annual income of vessel owners. Box and whisker plots show the distribution of cost across Hai Phong, Nam Dinh, and Ben Tre. The central line in each box represents the median; the bottom and top edges of the box represent the 25th and 75th percentiles (i.e. Q1–Q3 or interquartile range, IQR). Whiskers extend to the minimum and maximum values within 1.5× IQR. Significant differences in each cost component among the three study locations were tested using Kruskal–Wallis test (* *P* < 0.05, ** *P* < 0.01, *** *P* < 0.001 (See Supplementary Table 1 for detailed test statistics).

The fishers (*n* = 199) interviewed in our study incurred costs associated with plastic debris that were estimated at a median (Q1–Q3) value of 3421 (1848–6159) USD vessel^−1^ year^−1^ (Fig. 1). This amount is equivalent to 12 (7–18) % of their annual revenue and 25 (15–48) % of the fishing vessel owner’s income. The cost varied significantly among regions, ranging from 10% of the annual revenue of fishing vessels in Hai Phong and Nam Dinh in the Red River Delta, to 18% of the annual revenue of vessels in Ben Tre in the Mekong Delta (Kruskal–Wallis test, H_2, 164_ = 26.9, *P* < 0.0001) (Fig. 1d). This indicates nearly a two-fold higher impact in the Mekong Delta, the region with the highest fishery production.

The estimated total cost for trawl vessels and gillnetting vessels was not significantly different (H_1,199_ = 1.48, *P* = 0.4) with the median annual loss of 3356 and 4967 USD vessel^−1^ year^−1^, respectively (See Fig. 1a, b for details). The economic impacts of marine plastic debris expressed as lost annual revenue were 18 (9−29%) for gillnetters and 11 (7−18%) for trawlers in our study.

Taken across all study areas, contributions to the total economic cost of the impact of plastic on fishers included: 54% from lost revenue due to downtime that would otherwise be spent fishing, 28% from reduced catch efficiency, 12% from labour costs to remove plastics from the catch, 5% from fishing gear repair expenses, 1% from removing entangled plastics from propellers (See Supplementary Table 1 for detailed data).

The total labour downtime caused by marine plastic debris was 158 (62−360) hours vessel^−1^ year^−1^, equivalent to 6.5 (2−13) % of the total time spent fishing (See Supplementary Table 4). Plastic debris accumulated in the catch and nets alone increased total labour time associated with sorting catch from plastic debris by 15 (8−30) minutes per 3-h haul. Overall, 63% of the participants reported that they encountered marine plastics in their net almost every haul, and a further 18% said they frequently encountered plastics in 50−80% of their hauls.

Marine plastic debris also cause a high number of safety incidents while at sea. Among the 199 fishers interviewed, 45% (*n* = 90 fishers) reported propeller entanglement with discarded or lost ropes and nets, with a median of 4 (1−9) incidents per year (See Supplementary Table 4 for data by location and gear). During focus group discussions (FGD) conducted in Nam Dinh and Ben Tre, fishers reported incidents where attempts by fishers to untangle debris from vessel propellers resulted in several cases of serious injuries or tragic fatalities. One fisher reported:

“*I have experienced it myself, my hands were cut by barnacles while trying to remove rope caught in my propeller; … in this area people have died or* [had limbs] *amputated while doing it… they were pulled in the propeller when the old engine gear box malfunctioned…*”.

During the FGD, another fisher stated:

“*There was one person … well, the person above* [on the deck of the vessel] *shifted the gear and the man below* [trying to remove entangled propeller] *was chopped to death by the propeller*”.

Verification of the cost estimates above was derived from follow-up semi-structured verification interviews with 94 fishers who did not participate in the previous socioeconomic impact survey (See Methods). Among the interviewees, 77% of fishers agreed, 16% disagreed, and 7% partially agreed with the findings regarding the total cost of marine plastic debris on fishers’ incomes (Supplementary Table 9). Those who partially agreed noted that marine debris does not equally affect all fishing vessels stating that nearshore vessels and trawlers were impacted the most.

### Plastic debris composition based on fisher knowledge and waste audits

Fishers (*n* = 195) reported that they catch a median of ~15 (5−35) kg of mixed marine debris in each haul. They estimated that 17% of the mixed marine debris or 2 (1−5) kg by mass of the marine debris in each haul was plastic (See Supplementary Table 4 for data by location and gear type). The most frequently encountered plastic items were plastic bags, plastic bottles, single-use plastic items, fabrics, aquaculture feed bags, and ropes. Fishers (*n* = 165) provided information about the geographical distribution of encounters with plastic debris, such that fishers reported that they encountered more plastic debris in either the coastal zone (85% of responses) or the inshore area (13% of responses), with only 3% reporting more plastic in the offshore zone. Most fishers reported that marine plastics hotspots occurred at the outlets of rivers and agri/aquaculture sluice gates, especially during the rainy season from May to October when plastic transport from the land is greatest (See Supplementary Table 5 for detail reported hotspots).

**Fig. 2:**
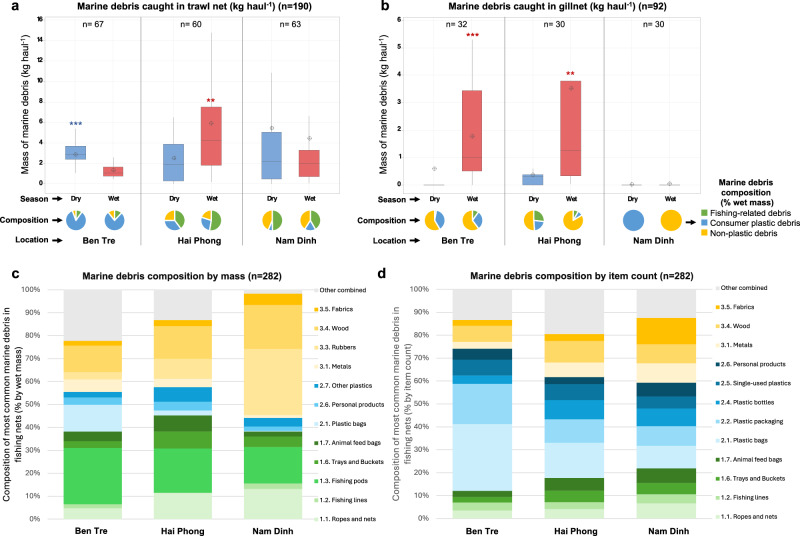
Waste audit of marine debris caught by nearshore fishing vessels in the studied areas during the dry and wet seasons. Mass (shown in box plot as kg haul^−1^) and composition (shown in pie charts as average percentage by mass) of marine debris caught by trawl nets (**a**, *n =* 190) and gillnets (**b**, *n =* 92) during dry and wet season. The box and whisker plots with the central line in each box represents the median; the circle symbol represents the mean; the bottom and top edges of the box represent the 25th and 75th percentiles (i.e. Q1–Q3 or interquartile range, IQR). Whiskers extend to the minimum and maximum values within 1.5× IQR. Note that the y-axis scale of (**a**, **b**) are not equal. Significant differences between marine debris caught in nets between the dry and wet season was analysed separately for each study location using a Kruskal–Wallis test *P < 0.05, **P < 0.01, ***P < 0.001 (detailed test statistics are available in Supplementary Table 2). The twelve most dominant categories of marine debris caught in each haul across seasons ranked by mass (**c**, *n =* 282, % by wet mass) and item count (**d**, *n =* 282, % by item count). Green, blue, and yellow shades of colour represent fishing-related debris, consumer plastic debris, and non-plastic debris, respectively.

**Fig. 3:**
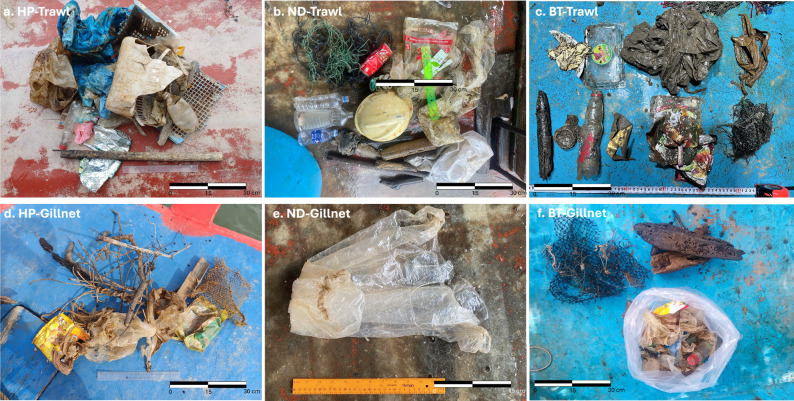
Visual examples of marine debris caught by nearshore fishing vessels. The images illustrate debris recovered from a single haul by trawlers (**a**–**c**) and gillnetters (**d**–**f**) during the waste audit in Hai Phong (HP), Nam Dinh (ND), and Ben Tre (BT); corresponding quantitative data are presented in Fig. 2.

The waste audit of marine debris caught by fishing vessels revealed that there was a median of 1.3 (0.2−3.4) kg of mixed marine debris haul^−1^ and 0.6 (0.01–2.6) kg haul^−1^ was plastic, measured on a wet mass basis (Fig. 2). The heaviest marine debris recovered was predominantly associated with fishing activities, whereas the most frequently encountered items by abundance were consumer plastics, including both domestic and international brand labels (Fig. 3).

Although the participating fishers overestimated the mass of marine debris (H_1,__477_* =* 222, *P <* 0.0001) and plastic debris (H_1,__477_* =* 73, *P <* 0.0001) compared to the waste audit data by 11 and 3 times, respectively, it is worth noting that their estimate of plastic debris is much more accurate than their perception of overall marine debris in their catches. This overestimation likely arises because fishers tend to base their estimates on occasions when debris is present in their nets, while overlooking periods with little or no plastic. When waste audit observations below 0.5 kg plastic per haul were excluded, fishers’ estimates closely matched field measurements, with no significant difference between estimated (2 kg haul⁻¹) and observed values (2.4 kg haul⁻¹; Kruskal–Wallis test, H_1,351_* =* 0.18, *P =* 0.7). We also acknowledge that the limited spatial and temporal coverage of the sampling campaign may not capture the variability in fishing conditions, which could contribute to the observed differences. Fishers demonstrated their local ecological knowledge on marine plastic debris by reliably recalling the most common marine debris items and their increased abundance during the wet season, including the seasonal fluctuation in Ben Tre province (Fig. 2 and Supplementary Table 5). Together, these results indicate that fishers’ local ecological knowledge can provide a reliable and cost-effective complement to field-based sampling for estimating and monitoring marine plastic debris.

### Fishers’ perceptions of potential interventions

Participating fishers were aware of the potential negative impacts of marine plastic debris and abandoned, lost, or otherwise discarded fishing gear (ALDFG) on the environment, fish stocks, and their livelihoods (See Supplementary Table 7). Despite this awareness, 57% of fishers felt unable to take action to reduce the impact of marine plastics. The majority (77%) continued to discard marine debris caught in their nets back into the ocean, with 18% only retaining only valuable items to sell or bringing back large objects to prevent future encounters. This mindset was encapsulated in a common sentiment:

“*I throw it back to the ocean, why should I bring it back? If I bring back but other people still throw the trash out, it will waste my efforts*”

Most fishers were interested in participating in a 'fishing for litter programme', whereby they would be compensated for collecting and bringing marine litter back to shore (*n* = 196, in which 56% strongly agree and 23% agree). In general, fishers indicated that they would need to be paid a median buyback price of 0.16 (0.08−0.23) USD kg^−1^ of marine plastic debris, with no significant difference among gill net or trawl net fishers (H_1, 119_ = 0.34, *p* = 0.6), although fishers in Hai Phong requested a higher marine litter buy-back price of 0.25 USD kg^−1^ of marine plastic debris (H_1, 119_ = 19.9, *P* < 0.001). A fisher in Hai Phong noted when asked about monetary incentive:

“*The buying price for marine plastic must* [be] *higher than the current price on land, otherwise no one will bring back…just throw it back to the sea to continue another haul*”. (See Supplementary Table 7).

### Inequities in the impacts of plastic pollution

Our findings demonstrate the substantial negative impact of marine plastic on the income, livelihoods and safety of nearshore trawl and gillnet fishers, with pronounced regional inequities.

The median annual economic loss from marine plastic debris per nearshore fishing vessel was estimated at USD 3421, comprising direct costs of USD 594 and lost opportunity costs of USD 2816. This exceeds reported losses in central Viet Nam^26^ (1364 USD year^−1^), Ecuador^27^ (569 USD year^−1^) and Peru^27^ (669 USD year^−1^). However, it is lower than estimates from higher-income countries, such as the Azores in Portugal^28^ (3502 USD year^−1^) and Scotland^29^ (20–23,000 USD year^−1^). The higher estimates relative to comparable regions in Viet Nam likely reflect the inclusion of lost opportunity costs in our method, which were omitted in previous studies. When restricted to direct costs, our estimates are consistent with earlier findings (Supplementary Table 8).

Although the economic loss between gillnet and trawl vessels was not significantly different (H_1,199_ = 1.48, *P* = 0.40), gillnet fishers reported encountering less marine debris in their nets (median 7 kg haul⁻¹) than trawlers (15 kg haul⁻¹), yet gillnet fishers spent 35% more labour time disentangling debris from their gillnet, resulted in 48% higher total economic loss compared to trawlers.

The cost burden varied from 10 (6−16) % of annual revenue of fishing vessels in Hai Phong and Nam Dinh in the Red River Delta to 18 (10−28) % of annual revenue of vessels in Ben Tre in the Mekong Delta (Fig. 1d). Land-based marine plastics were found to be primarily confined to the coastal zone^13,30^ and concentrated at outlets of major river systems such as the Mekong River in Ben Tre^6^, hence coastal fishing communities bear the greatest cost burden compared to offshore fishers. Although the total absolute cost of plastic litter to fishing vessels was not significantly different between our case-study regions, the relative cost in terms of percentage of revenue loss was significantly higher for fishers in the Mekong Ben Tre province (Fig. 1c, d). This disparity is likely a result of nearshore fishers in Ben Tre that had significantly lower annual revenue (711 USD vessel^−1^, H_2, 198_ = 23.33, *P* < 0.0001) and lower fishing yields (50 kg trip^−1^, H_2, 192_ = 41.07, *P* < 0.0001) than fishers in the Red River’s Nam Dinh and Hai Phong provinces, making them more vulnerable to the additional economic impacts of marine plastic pollution. Global inequalities in plastic pollution are known to occur such that lower income countries experience a 10.6 times higher cost from plastic pollution than higher income countries^31,32^. Income level is a key determinant of vulnerability to plastic pollution^33^. Lower-income coastal communities often have poorly developed waste management infrastructure, fewer alternatives for livelihoods, limited capacity to avoid or mitigate pollution impacts, and greater dependence on degraded marine ecosystems^31^. Plastic waste exacerbates inequality, with coastal and marginalised communities bearing disproportionate social, environmental, and health harms across all stages of the plastic lifecycle, despite contributing least to this problem and having limited influence over decision-making^34^.

The regional differences in this study are linked to several demographic factors (Supplementary Tables 3, 6). Smaller-scale fishers in Ben Tre were the most economically vulnerable to the impacts of plastic pollution because typically they had older vessels with smaller and less fuel-efficient engines and had lower catch efficiency compared to fishers from the Red River Delta front^35–37^. Furthermore, nearshore fishers in Ben Tre operated at the outlet of two of the world’s most plastic-polluted rivers, the Mekong River and Saigon-Dong Nai River systems^6^. Plastic leakage to the coastal and nearshore environment in Ben Tre province is three times higher compared to the Red River, Hai Phong and Nam Dinh in the north of Viet Nam^38^ (See Supplementary Table 6). This was reflected in our data from the waste audit, which showed significantly more plastic debris caught in the fishing nets of fishers in Ben Tre compared to fishers in Nam Dinh (H_2,283_ = 15.92, *P* < 0.001). Ben Tre fishers cannot move to alternative fishing grounds to avoid plastic pollution as fuel accounted for 64% of their operational costs. This was underlined by 53% of the interviewed fishers (*n* = 68) who considered that they cannot do anything to alleviate the impact from marine debris:

“*I have to accept it, what else can I do?*”.

Our study also revealed that marine debris resulted in serious accidents, especially when crew members need to untangle fishing nets and ropes from propellers. Although marine plastic-related accidents were rare and not all fishers directly witnessed the incidents (making it difficult to verify whether the entangled ropes originated from their own fishing vessels or from ALDFG), the accidents reported emphasise the potentially severe risks to nearshore fishers’ health and safety. Reports of accidents leading to crew injuries, casualties, or man-overboard situations caused by marine debris has been previously reported in other countries^18,39^. Thus, plastic pollution in the coastal ocean has cumulative impacts on nearshore fishing communities, thereby contributing to the lifetime cost of marine plastic pollution^16^. Marine plastic also compounds other challenges confronting these fishers such as the impacts of extreme weather events, reductions in biodiversity, depletion of fish stocks, and conflicts over-fishing zones^40^. These interconnected issues intensify the pressure on vulnerable communities, making their coastal livelihoods increasingly precarious as river delta economies continue to grow and exacerbate the plastic waste problem.

### Lack of comparable data among socioeconomic impact assessment studies

Our current understanding of the socioeconomic impacts of marine plastic in Viet Nam and elsewhere is scarce and lacks standardized data collection approaches^30^. Previous studies often only accounted for direct costs (i.e., gear damage, repair cost, time spent sorting catch from plastic)^28^ and did not include lost opportunity cost. We account for this here and found that that it is a substantial component of total cost (i.e. potential revenue from time lost that could otherwise be spent fishing, and reduction in catch due to interference with gear). Although fishers are often acutely aware of these opportunity losses, they rarely quantify them in monetary terms. For example, one fisher noted in interview:

“*We have to catch two tonnes a day before going home* (to have profit)*. Before, 6−7 hauls were enough for two tons; now it takes 20−25 hauls*”.

This illustrates how the combination of over-exploited fish stocks and plastic pollution increases the effort required to achieve the same catch, translating into substantial economic losses that are but frequently unaccounted for. Our results show that incorporation of these factors into socioeconomic assessments is critical, as their omission can lead to a systematic underestimation of the true economic impacts of plastic pollution on small-scale fisheries and limit the relevance of cost estimates for management and policy decisions.

The direct costs estimated in this study are between 0.6 and 3.6 times higher than previously reported values for Viet Nam, which could be associated with differences in local demographics and environmental context (Supplementary Table 8). Direct comparison with other empirical and large-scale modelling estimates from different locations worldwide remains challenging due to the absence of standardized methods and protocols, as well as differences in the scope and types of cost components and impact factors quantified across studies^28^ (See Supplementary Table 8 for detailed comparisons). The lack of consistent reporting units and economic estimation methods have been identified as one of the challenges in comparing the impacts of plastic pollution and ALDFG^41^. Detailed guidelines on quantifying marine debris and its impacts are therefore essential for quantifying pollution impacts^42^.

### Using local knowledge to identify and mitigate plastic pollution hotspots

Identifying spatiotemporal hotspots of marine plastic debris is essential for guiding targeted mitigation and cleanup efforts. While numerical models can simulate large-scale and long-term plastic transport via ocean currents, they require validation through field observations, which are often costly and spatially or temporally limited. Local ecological knowledge from fishers, who spend extensive time at sea and rely on understanding marine dynamics for their livelihoods, offers a valuable resource to bridge these knowledge gaps and enhance hotspot detection.

Results from the waste audit (Fig. 2) and surveys with fishing communities (See Supplementary Table 5) were aligned, indicating that local knowledge was a valuable and reliable source of information to identify plastic pollution hotspots, seasonal variability, diversity of waste items and key contributors to plastic pollution in the local area. The scientific reliability and accuracy of fishers’ knowledge about the distribution of plastic is also reflected by the fact that the reported hotspots (See Supplementary Table 5) agreed with previous predictions of macroplastic transport dynamics exported from Ba Lat River of the Red River system^13^. Local knowledge held by fishers has been widely used in conservation and informed context-specific problems that are often missed by top-down assessments^43^. Empirical evidence from Viet Nam^26^, Cameroon^44^, and European countries^45,46^ shows that incorporating fishers’ knowledge supports the co-design of targeted ALDFG prevention, retrieval schemes, and improved port waste management, leading to more practical and locally accepted interventions. Local ecological knowledge therefore complements empirical scientific data and, when combined, can be used to inform co-management for locating and tackling marine plastic debris^26,43^. On the other hand, once corroborated with local knowledge, field sampling data and plastics transport modelling may inform best practices and optimal locations for beach and ocean waste cleanup operations. For example, previous numerical modelling^13^ suggests that during the dry season plastics are pushed inshore in northern Viet Nam, creating a potential window of opportunity for removing plastics from the beach - which can be done at a much lower cost than from boats at sea.

### Solutions to mitigate impacts of plastic pollution on nearshore fishing communities

Fishers’ preferred solution to actively engage in the marine pollution problem was an incentivised 'fishing for litter programme', whereby fishers would be compensated for collecting marine plastic waste. Our data show that enrolment in such a programme should offer a median buyback price of only 0.16 (0.08−0.23) USD kg^−1^ of marine plastic debris, or as fishers often mentioned during the interviews as “*equivalent to the market price of trash fish*”.

Using waste audit data, each fishing boat was estimated to catch a median of 0.60 (0.01–2.55 kg) of marine plastic per haul, with a median of 630 hauls year^−1^. A fishing for litter programme would therefore need to pay each participating vessel ~60 USD year^−1^ to bring all collected marine plastic ashore (equivalent to 378 kg of plastic vessel^−1^ year^−1^). The additional cost of treating the returned plastic would be around 20 USD vessel^−1^ year^−1^, assuming a waste treatment rate of 54 USD ton^−1^ in 2025 at an annual inflation rate of 5%^47^.

Such incentivised programs have been piloted in Viet Nam^48^ and European countries^45,46^ with initial success. However, these pilots often failed to consider socioeconomic inequalities when selecting implementation sites. Our study provides an important recommendation that any fishing for litter initiative should be prioritised at plastic cost hotspots for maximum alleviation of economic impact of marine plastic for the most vulnerable fishing communities. We suggest that combining local knowledge from fishers (Supplementary Table 5) with field observations^49^ and hydrodynamic-based pollution modelling^13^ could be applied more widely to inform a strategic plan to optimise the timing and location of coastal and marine debris cleanup initiatives.

Future research should relate data on highly plastic-polluted rivers^6^ to the location of low-income, fisheries-dependent areas to identify priority locations^33^ where marine plastic collection incentive programmes could be implemented. Based on a rapid, exploratory analysis of currently available datasets, we identified indicative priority locations for Viet Nam and globally (See Supplementary Table 10 for the list of prioritised locations). Two branches of the Mekong River system (Hau River and Vam Co River) were among the top 10 priority candidate rivers for Viet Nam. Rivers in the Philippines and India^6^ were also identified as potential candidates due to their high plastic output and occurrence of lower-income communities. Sustainable solutions must also consider educational campaigns that explain the real cost of plastic litter, the benefits of recycling and other systemic changes. Such systems-change approaches should enhance both the hardware (physical/environmental changes) by increasing solid waste collection rates from upstream to downstream, and the software (societal changes) by altering social norms in solid waste management through legal and societal reinforcement.

## Conclusions

This study highlights the economic and social burdens faced by small-scale nearshore fishing communities in delta front regions of the Mekong and the Red River in Viet Nam. Based on reports from 199 fishers and related stakeholders, the average cost on nearshore fishers was estimated to be 10%, 10.6%, and 18% of the annual revenue of each fishing vessel in the Red River Hai Phong City and Nam Dinh, and the Mekong River Ben Tre provinces, respectively. However, we also found significant inequalities in the economic impacts of marine plastic debris. Nearshore fishers operating close to the Mekong estuaries faced the strongest environmental and social stressors and were likely to experience greater impacts compared to Red River areas. Furthermore, fishers reported higher health and safety risks, particularly during the monsoon season, due to plastic entanglement incidents. Local fishers demonstrated a strong understanding of where and when hotspots of marine plastics may occur and proposed solutions in which they are willing to participate if centrally managed. These findings are corroborated by data from the validation survey with additional groups of fishers and by field-based waste audits on fishing vessels. Fishers emphasised the need for incentive-based marine plastic collection programmes (median price of 0.16 USD kg^−1^ of marine plastic debris), prioritised at socioeconomic hotspots where plastic pollution most severely affects low-income coastal communities. The new results from this study serve as components of a framework for understanding economic impact hotspots and co-designing effective solutions with community participation to tackle marine plastic pollution in coastal areas worldwide.

## Methods

### Location and scope of study

**Fig. 4:**
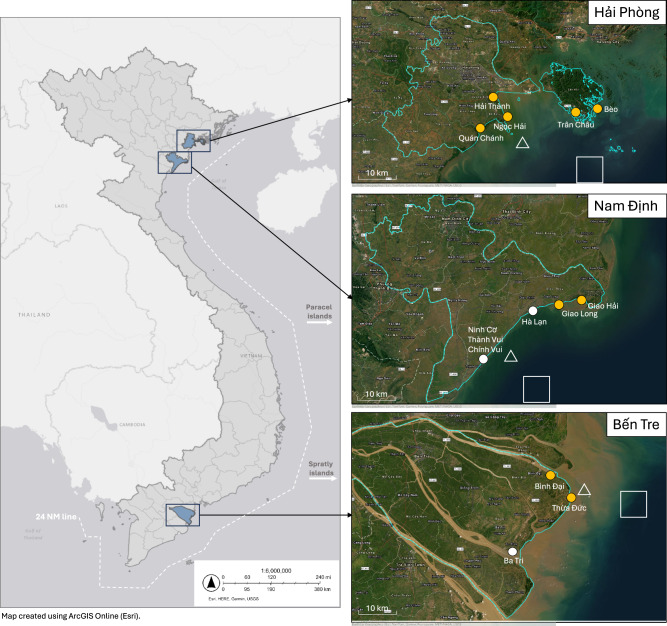
Locations of the study areas in Hai Phong, Nam Dinh, and Ben Tre. Yellow dots are the locations of fishing ports and communities involved in the socioeconomic impacts survey (*n =* 199 fishers); white dots are additional fishing port locations included in the verification survey (*n =* 94 fishers). Triangles show the locations of waste audits on gillnetters (*n =* 92 observations); rectangles indicate the locations of waste audits on trawlers (*n =* 190 observations).

This study focused on small-scale nearshore fishing communities in three regions: Hai Phong City (HP), Nam Dinh Province (ND), and Ben Tre Province (BT) (Fig. 4). These administrative units are reported with their boundaries shown as before the provincial consolidation effective 1 July 2025. These sites were selected for their contrasting geographical and socioeconomic characteristics (Supplementary Table 6). Hai Phong represents a coastal and island-dominated area with high income, important fishing landing sites, maritime shipping industries, and tourism destinations. Nam Dinh and Hai Phong are located at the outlets of the Red River in the north of Viet Nam. Ben Tre is adjacent to the Mekong River in the south of Viet Nam and is characterised by small-scale fisheries, aquaculture, agriculture, and other coastal businesses. Previous studies show that these two rivers are among the top rivers in the world with regard to the discharge of plastic waste into the coastline and ocean^6,50^.

In this study, the term “nearshore” refers to both coastal and inshore fishing zones which are defined by Vietnamese fisheries law to include the area from the shore out to 24 nautical miles. Previous research indicates that globally up to 77% of buoyant ocean plastics are concentrated in the coastal zone^30^. We focused on small-scale fishing vessels owned and operated by local families using trawls and gillnets in nearshore waters that are disproportionately impacted by plastic debris^23,26,51^.

### Data collection

**Fig. 5:**
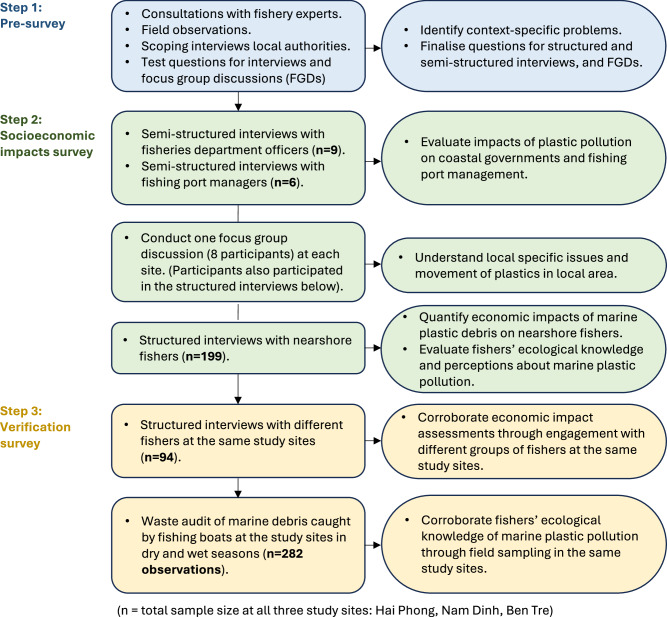
Data collection procedure of the mixed method design followed in each study location.

The study was conducted following a methodology of mixed methods^52^ that were implemented in a consistent sequence at each field site (Fig. 5). Participants were selected through a combination of snowball sampling, initiated with key informants (e.g., governmental officers from the Department of Fisheries or leaders of fishing groups within the commune), and random sampling with fishers at different ports (Fig. 4), allowing us to collect data from a total of 308 individuals. A total of fifteen semi-structured interviews with fisheries governmental officers (*n* = 9) and fishing port managers (*n* = 6), and three focus group discussions with fishers (*n* = 8 participants each, who also participated in the socioeconomic impact survey) were conducted across the three field sites. The socioeconomic impact survey consisted of 199 structured interviews with nearshore fishers (see demographics in Supplementary Table 3) to quantify the economic impacts of marine plastic debris on their livelihoods. A verification survey using structured interviews was conducted with fishers who had not participated in the previous socioeconomic impact survey (*n* = 94) to assess the extent of agreement or disagreement that fishers had with the research findings. In both surveys, we used a combination of quantitative and qualitative interview questions, including Likert-scale measures of agreement to and open-ended questions. When designing the survey, we acknowledged that Likert scale questions primarily capture direction (positive or negative) and to a lesser degree intensity (level of agreement or disagreement). While binary answer questions (“Yes” and “No”) were considered, we wanted to capture the intensity of beliefs especially in relation to perceptions about decreasing fisheries resources, increasing risks to crew, longevity of plastic waste, and motivations to bring plastics back to shore^53^. The 5-point Likert scale was chosen over the 7-point alternative due to its ease of administration and reduced completion time.

A waste audit survey (*n* = 282 observations) was conducted in the same study sites with the socioeconomic impact interviews to corroborate the quantification of marine plastic debris encountered in fishing nets. Detailed data collection procedure, calculation methods, and data limitations are available in the Supplementary Methods.

### Cost estimation

Using the data from the socioeconomic impact survey with 199 fishers, the economic costs were calculated with the following formula:

$${{\rm{Total\,Cost}}}({{\rm{TC}}})= 	 {{\rm{Direct\,cost}}}\,({{\rm{DC}}}={{\rm{DC}}}1+{{\rm{DC}}}2+{{\rm{DC}}}3)\\ 	 +{{\rm{Lost\; opportunitycost}}}\,({{\rm{LC}}}={{\rm{LC}}}1+{{\rm{LC}}}2)$$

Where:

DC1 = Labour cost of crew members’ combined time spent separating plastic debris from fishing gear and the catch

DC2 = Labour cost of crew member’s combined time spent untangling plastic debris from propellers, cooling water inlets etc.

DC3 = Cost of replacing and fixing damaged gears from encountering plastic debris and/or towing vessel to port for repairing if the vessel was disabled due to plastic debris

LC1 = Loss of revenue from reducing fishing efficiency due to plastic debris by tearing nets, blocking nets, and causing nets to float, among other issues.

LC2 = Loss of revenue from total time lost when the fishing vessel could have been fishing

The costs incurred from encounters with marine plastic debris were calculated individually for each vessel, based on fishers’ responses and estimates. Each of the five components to the total cost was calculated based on multiple contributing factors, including: type of plastic-related incidents (non-exhaustive list includes: stuck in fishing nets, mix in the catch, entangle in propeller, block water inlet, damage fishing net, etc.), frequency of each incident (incidents per year), time loss (hours per incident), number of people involved (people per incident), labour cost (USD per working hour), repair cost (USD per incident), estimated reduction in catch efficiency (% reduction), revenue per unit effort (USD per hour fishing), and estimated plastic content in mixed marine debris (% by wet mass). All interviews were conducted in Vietnamese and cost was calculated in local currency before converted to USD, details on the calculation of cost are available in Supplementary Methods.

The direct cost represents expenses that fishers could recall in detail, making them more reliable. The lost opportunity cost uses fishers’ estimation of catch efficiency reduction due to plastic debris and loss of revenue per unit time loss, and therefore is subject to a higher degree of uncertainty and fluctuations^28,35^.

### Waste audit

A waste audit was conducted on board fishing vessels by observers following the method from GESAMP (2019)^54^ (See Supplementary Methods). A total of six small-scale fishing vessels (one trawler and one gillnetter from each of the three study areas) were contracted to participate in the study on the spatiotemporal distribution of marine litter in nearshore waters. In total, we collected 282 observations (190 hauls from trawlers and 92 hauls from gillnetters), split between the three study areas and across both the dry season (17th April to 30th May, 2024) and wet season (1st August to 30th September, 2024). The same vessel and gear were used in both sampling periods to compare seasonal variability. However, the fishing gear in the three locations had slight differences. The bottom trawlers had nets with a reported mesh size of 25−35 mm and operated at a maximum water depth of 10−36 m, while the gillnetters used nets with a mesh size of 45−55 mm and were deployed at a water depth of 5−10 m from the water surface.

### Data analyses

Anonymized interview responses were entered, coded and screened for missing data and invalid answers (i.e. blank and “I don’t know” answers). Audio recordings of the semi-structured interviews and FGDs were transcribed, translated, and coded in NVivo 14 (Version 14.23.2). Data analysis was conducted using IBM SPSS Statistics software (Version 29.0.1). The estimated costs and other data reported by interviewers were non-normally distributed (Shapiro–Wilk test, *P* < 0.001). Therefore, we reported all values as medians (Q1–Q3). Significant differences were tested using Kruskal–Wallis test with the test statistic *H*-value (degree of freedom (df), total samples (*n*)), presented as H_df, n_ and associated *P*-values. Detailed results of data analyses are available in Supplementary Tables in Supplementary Information.

### Ethics approval

Ethics declaration data in this study were collected and protected following the Equality and Privacy Impact Assessment and Data Management Plan approved for the project “Sources, Sinks and Solutions for Impacts of Plastics on Coastal Communities in Viet Nam” (3SIP2C) (NE/V006088/1). The research was subject to full ethics approval through the Heriot-Watt University ethics committee: approval number 2023-3366-7400.

### Reporting summary

Further information on research design is available in the Nature Portfolio Reporting Summary linked to this article.

## Supplementary information

Transparent Peer Review file

Supplmentary information

Reporting Summary

## Data Availability

The data for graphs in this article are publicly available through Figshare at**:** 10.6084/m9.figshare.31882999. The raw datasets used in this study are included in the article, Supplementary Information and are publicly available through PURE at 10.17861/47e741f5-3810-4f40-9b03-fba8cf825913. Any additional information is available upon request to the corresponding author.
