# Supplementary material for: Marine plastic pollution undermines the livelihoods and income of fishing communities in Viet Nam
Source: Commun Earth Environ. 2026 Jun 8;7(1):452. doi: 10.1038/s43247-026-03567-z (PMC13246506; doi:10.1038/s43247-026-03567-z)
Supplement: Supplementary file 2 — Supplmentary information [file 43247_2026_3567_MOESM2_ESM.pdf]

Supplementary Information

Marine plastic pollution undermines the livelihoods and income of fishing communities in Viet Nam

Duc Nguyen[1], Phan Phuong Thanh[2], Heidi L. Burdett[3,4], Inna Yaneva-Toraman[1], Zhiling Liao[1], Qingping Zou[1], Trinh Quang Tu[2], Le Trung Dung[2], Le Thi Thu Huong[2], Ryan Pereira[1], Thomas Wagner[1], Huong Thi Thuy Ngo[5,6], Michel J. Kaiser[1*]

Affiliations:

[1] The Lyell Centre, Heriot-Watt University, Riccarton, Edinburgh, United Kingdom

[2] Research Center of Fisheries Economics and Planning, Vietnam Academy of Fisheries Sciences, No. 10 Nguyen Cong Hoan Street, Giang Vo Ward, Hanoi, Viet Nam

[3] Umeå Marine Sciences Centre, Umeå University, Norrbyn, Sweden

[4] Department of Ecology, Environment and Geoscience, Umeå University, Umeå, Sweden

[5] Environmental Chemistry and Ecotoxicology Lab, Phenikaa University, Duong Noi, Hanoi, Vietnam.

[6] Faculty of Biotechnology, Chemistry and Environmental Engineering, Phenikaa School of Enginerring, Phenikaa University, Duong Noi, Hanoi, Vietnam

*Corresponding author: m.kaiser@hw.ac.uk

Table of content for Supplementary Information

Supplementary Information 1: 3

Supplementary Methods 3

1. Socioeconomic survey steps: 3

2. Method for cost calculation 4

3. Method for waste audit..4

4. Data limitation..5

Supplementary Information 2: 7

Supplementary Tables...7

Supplementary Table 1: Statistical analysis and data for Figure 1.............................7

Supplementary Table 2: Satistical analysis and data for Figure 2...............................9

Supplementary Table 3: Demographic data of fishers who participated in the socioeconomic impact survey. 11

Supplementary Table 4: Frequency of encountering marine plastic debris. 12

Supplementary Table 5: Highest frequency answers in identifying plastic debris hotspots through local ecological knowledge....13

Supplementary Table 6: Socioeconomic, geoeconomic, and fishery features of the three studied locations....14

Supplementary Table 7: Knowledge and attitude of fishers about ALDFG and marine plastic debris. 15

Supplementary Table 8: Socioeconomic impacts of marine plastic debris on fishing vessels in Viet Nam compared to previous research 18

Supplementary Table 9: Results of the verification survey.......................................20

Supplementary Table 10: List of prioritised locations to launch incentive programme to bring back marine plastic debris. 21

Supplementary References 24

Supplementary Methods 25

Interviews and Informed Consent Form 25

Supplementary Information 1:

Supplementary Methods

1. Socioeconomic survey steps:

Data collection procedure in each of the study locations is presented in Figure 5 of the manuscript. A total of fifteen semi-structured interviews with fisheries governmental officers (n=9) and fishing port managers (n=6), and three focus group discussions (FGDs) (n=8 participants each) with fishers were conducted across the three field sites. The qualitative data collected through semi-structured interviews and FGDs provided information about context-specific issues and concerns relevant to the local fisheries and complemented the quantitative data on economic, social, and environmental aspects of fisheries businesses and operations gathered through structured interviews.

In all structured interviews, we used a combination of quantitative and qualitative interview questions, including Likert-scale measures of agreement (1 = Strongly disagree to 5 = Strongly agree to a statement) and open-ended questions (Interview forms include below). The socioeconomic impact survey consisted of 199 structured interviews with small-scale nearshore fishers to quantify the economic impacts of marine plastic debris on their livelihoods. Five researchers carried out these interviews in person across the three field sites between March and July 2023. The sample size allowed for a confidence interval (CI) of 95% and a margin of error (ME) of 7%. Fishers' participation was influenced by seasonal fishing patterns and availability resulting in an uneven distribution among metiers (74% trawlers and 26% gillnetters) (Supplementary Table 3). The structured interviews included the following sections: informed consent, characteristics of fishing vessels and operations, trends in landing yields, details of plastic waste removed from the ocean and subsequently the vessel, current waste management methods used on the vessel, economic impacts of marine plastic debris on fishing operations, knowledge and perceptions about the impact of, and solutions for ALDFG and plastic debris, and demographic and socioeconomic c information. Each interview averaged approximately 45 minutes. While conducting these interviews was time-consuming and required a lot of effort and resources, it provided a better opportunity to build rapport, secure participants' investment, and allow interviewees to ask for clarifications. It also provided a space for participants to offer insights beyond the scope of the interview instrument, that were useful in comprehending the wider context and designing follow-up research activities.

A verification survey using structured interviews (n=94) was conducted in May 2024 to assess the extent of agreement or disagreement that fishers had with the research findings. A total of 94 randomly selected fishers, who operated in all fishing zones (i.e. nearshore and offshore) across all major fishing ports of the three study locations, were interviewed. These were fishers who had not participated in the previous survey, to cross-validate the research findings about the total estimated cost of marine plastic debris on nearshore fishers. [1]

All interviews were conducted by four researchers using a standardised interview protocol. Prior to data collection, all researchers jointly test and revise the interview form to refine question wording and align the interview process (Figure 5 in the main manuscript). This training ensured consistency in delivery and minimised interviewer-induced bias across surveys.

2. Method for cost calculation

Cost calculation detailed equation:

$$TC \quad = (\sum_{i=1}^{2} F_i N_i T_i CL_i P_{\%} + \sum_{i=3}^{5} F_i N_i T_i CL_i P_{\%} + \sum_{i=3}^{5} F_i CR_i P_{\%}) + (\sum_{i=5}^{6} F_i R_i E_i P_{\%} + \sum_{i=1}^{6} T_i R_i E_i P_{\%})$$

In which,

TC = total estimated cost of plastic waste on fishing vessels (*USD/vessel/year*)

i = type of plastic-related incidents reported by fisher, where:

1 = stuck in fishing nets

2 = mix in the catch

3 = entangle in propeller

4 = block water inlet, etc

5 = damage fishing net

6 = reduce catch efficiency from blocking net, etc

F_i = Frequency of the incident (incidents/year)

N_i = number of labour(s) involved in dealing with the incident (person/incident)

T_i = time to deal with the incident (hour/person)

CL_i = labour cost (USD/hour)

CR_i = repair cost (USD/incident)

E_i = reduction in efficiency (% reduction in catch)

Ri = revenue per unit effort (USD/hour)

$P_{\%}$ = estimated average plastic content in encountered marine debris (% by mass).

The cost is calculated in local currency (Vietnamese Dong-VND) and reported in US Dollar (USD) with the conversion rate of 1 USD = 25.564 VND (rate on 1st April 2025 on the website https://www.oanda.com/currency-converter).

A full sensitivity analysis was not conducted, as this study focuses on empirically quantifying observed socioeconomic impacts rather than developing a model-based economic assessment. A rapid one-way sensitivity check indicates that the total labour time lost due to marine plastic interference is the primary driver of total cost estimation, with other variables having comparatively smaller effects.

3. Method for waste audit

Waste categorisation

Only macro-litter items (items >25 mm) [1] were collected for quantification. Rocks, shells, plants, and bycatch were discounted. Since it was difficult to distinguish between anthropogenic and natural sources, both processed woods (e.g. wooden poles, planks, etc) and

unprocessed wood (e.g. bamboo poles, natural fishing aggregated devices- FADS) were included in the waste audit.

Marine debris was quantified following GESAMP guideline[2]. We categorised the marine debris following the UNEP-IOC code (in backets)[2] :

-Fishery-related plastic items were defined as items that were potentially used in fishing or aquaculture, sorted in 8 groups: fishing nets and ropes (PL19-20); monofilament lines (PL18); traps and pods (PL17: can include metal frame, which was included in the mass); foam buoys and fragments (FP03-04); plastic buoys (PL14); trays and buckets (PL03,13), feed bags (PL15-16), other specified fishery-related items.

-Consumer plastic items can include items generated from the fishing vessels and land-based sources, sorted into 7 groups: plastic bags (PL07); plastic packaging and wrappers, foamed cups and food packs (FP02), plastic bottles (PL01-02); single-use plastic items (PL04-06), plastic personal protective equipment and sanitary products (OT02); other specified consumer plastic items.

-Non-plastic items include five groups: Metals (ME), Glass & Ceramic (GC), Rubber (RB), Wood (WD), Textile (CL).

Marine plastic debris in this study is defined as both fishery-related and consumer plastic items. The majority of fishery-related items were made of plastic. Among 282 observations, we found a total of 24 items in the pots and traps category, which could contain metal frames, but were included in the total mass of the item.

Waste audit process

The marine debris was pre-sorted from the catch by the crew members, overseen by the observers, to ensure the collection of all marine debris. The onboard observers then categorised, counted, and measured the wet mass of debris using a standardised digital scale (maximum: 50 kg, resolution: 0.1 kg). Photos of the items were also taken to ensure waste audit quality and to provide a visual representation of the marine debris. The observers were experienced research staff from Research Institute for Marine Fisheries, who were trained on how to conduct a waste audit before field observations to ensure data collection and measurement consistency across locations.

4. Data limitation

This study is subject to several limitations inherent to its scope and methodology. The fisher survey, while targeting small-scale nearshore operations, was disproportionately composed of trawl fishers due to the limited presence of gillnet fishers at the surveyed port. As a result, the results of incurred costs should only be made for participants within the scope of the study. Direct comparisons between gear types and extrapolation to offshore fishing fleets or broader geographic areas should be made with caution and only in reference to the associated confidence intervals and margins of error provided in Supplementary Supplementary Table . This study focused on nearshore trawl and gillnet fishers at major estuaries, who are among the most severely affected by marine debris. Consequently, the cost estimates presented are likely to reflect the upper bound of the national impact in Viet Nam.

Estimates of time loss and plastic-related incidences were derived from fisher recollection and self-reporting, which are susceptible to recall bias. To mitigate this, we applied triangulation

through targeted field sampling and the waste audit. However, due to the scope of the study, this was conducted at a limited location and points in time and did not include a longitudinal sampling design. These constraints highlight the need for future research incorporating broader temporal coverage, multiple sites, and a broader and more balanced representation of fishing vessels.

Supplementary Information 2:

Supplementary Tables

Supplementary Table 1: Statistical analysis and data for Figure 1. The table shows the calculated direct cost, loss opportunity cost, and total cost of marine plastic debris on nearshore trawlers and gillnetters in each study location. Data are presented as median and interquartile range (IQR of Q1 - Q3). Significant difference between the three study locations was calculated for each of the cost components using Kruskal-Wallis test. Significance levels are indicated as *P<0.05, **P<0.01, ***P <0.001. Associated sample size (n), degree of freedom (df), and statistical value (H-value) of the test are reported.

Case	Variable	Ben Tre (Median)	Hai Phong (Median)	Nam Dinh (Median)	df	H-value	P-value	Overall across locations (Median (IQR))
**Fig. 1a Trawlers**	**N (total= 167)**	**51**	**55**	**61**				**167**
	Direct Cost (USD/vessel/year)	762**	366	391	2	12.52	0.002**	518 (188-1027)
	Loss Opportunity Cost (USD/vessel/year)	2816	2305	4130	2	4.74	0.093	2768 (1411-4895)
	Total Cost (USD/vessel/year)	3495	2783	4714	2	4.86	0.088	3356 (1848-5721)
**Fig. 1b Gillnetters**	**N (total= 32)**	**17**	**4**	**11**				**32**
	Direct Cost (USD/vessel/year)	1205	1012	1526	2	1.57	0.457	1164 (576-1655)
	Loss Opportunity Cost (USD/vessel/year)	1565*	4305	4929	2	6.60	0.037*	3286 (1450-5595)
	Total Cost (USD/vessel/year)	2672	6158	6464	2	5.79	0.055	4967 (1759-6910)
**Fig. 1c, d All nearshore fishing vessels**	**N (total = 199)**	**68**	**59**	**72**				**199**
	Direct Cost (USD/vessel/year)	785	399	597	2	12.17	0.002**	594 (222-1174)
	Loss Opportunity Cost (USD/vessel/year)	2817	2313	4274*	2	7.07	0.03*	2816 (1418-4929)

	Total Cost (USD/vessel/year)	3458	2826	4768	2	5.58	0.061	3421 (1848-6159)
	Total Cost as annual revenue of fishing vessel (% of revenue)	17.958***	9.593	10.599	2	26.9	0.000***	12 (7-19)
	Total Cost as annual income of fishing vessel's owner (% of income)	27.708	19.725	27.818	2	5.23	0.073	26 (15-48)
**Cost components (N=199)**	**Total cost =Direct cost (DC1+2+3) + Loss opportunity cost (LC1+2)**	**Ben Tre**	**Hai Phong**	**Nam Dinh**	**Overall across locations**			
	DC1- labour costs to remove plastics from the catch (% of total cost)	10.60%	11.77%	9.49%	11.70%			
	DC2- removing entangled plastics from propellers (% of total cost)	2.43%	4.03%	0.80%	1.04%			
	DC3- fishing gear repair expenses (% of total cost)	2.97%	8.54%	3.01%	4.55%			
	LC1- cost from reduced catch efficiency (% of total cost)	33.39%	31.42%	42.48%	28.35%			
	LC2- lost revenue due to downtime (% of total cost)	50.61%	44.23%	44.22%	54.37%			

Supplementary Table 2: Satistical analysis and data for Figure 2. The table shows waste audit data of marine debris caught by trawl net and gillnets in the same three study sites with socioeconomic impact survey. Data are presented as median. Significant difference between the dry season and wet season in each of the study locations using Kruskal-Wallis test. Significance is indicated with *P<0.05, **P<0.01, ***P <0.001. Associated sample size (N), degree of freedom (df), and statistical value (H-value) of the test are reported.

	Marine debris in catch (kg/haul)	Dry season (Median) (kg/haul)	Wet season (Median) (kg/haul)	N	df	H-Value	P-Value
**Fig. 2a: Trawlers (N=190)**	Ben Tre	2.9	1.1	67	1	20.29	0.000***
	Hai Phong	1.97	4.32	60	1	7.56	0.006**
	Nam Dinh	2.22	1.99	63	1	0.09	0.762
**Fig. 2b: Gillnetters (N=92)**	Ben Tre	0	1	32	1	10.9	0.001***
	Hai Phong	0.305	1.265	30	1	9.42	0.002**
	Nam Dinh	0	0	30	1	0.27	0.605

**Fig. 2c,d: Combination (N=282)**	**Fig. 2c: Composition by mass (% wet mass) (N=282)**			**Fig. 2d: Composition by item count (% item counts) (N=282)**		
Categories of marine debris	Ben Tre (n=99)	Hai Phong (n=90)	Nam Dinh (n=93)	Ben Tre (n=99)	Hai Phong (n=90)	Nam Dinh (n=93)
1.1. Ropes and net	5.0	11.9	13.4	3.5	4.1	6.6
1.2. Fishing lines	1.8	0.1	2.4	3.5	3.1	4.0
1.3. Fishing pods	26	20.1	16.1	2.5	4.0	4.4
1.4. Float-Foam	0	0.5	0	0	3.1	0
1.5. Float-Hard plastics	0	5.8	0	0	3.1	0
1.6. Trays and buckets	3.1	7.7	4.6	2.5	5	4.9
1.7. Animal feed bags	4.4	7.3	2.2	2.7	5.5	6.4
2.1. Plastic bags	12.4	2.2	0.3	29.1	15.3	9.8
2.2. Plastic packaging	6.2	0.8	0.1	17.6	10.3	8.6
2.3. Foamed food packs	1.3	0	0	2.5	0	0
2.4. Plastic bottles	3.1	0.9	0.2	3.6	8.3	7.7
2.5. Single-used plastics	2.5	0.5	0.1	6.9	7.0	5.3
2.6. Personal products	3.3	4.0	1.9	4.8	3.1	6.0

2.7. Other consumer plastics	2.7	6.4	3.9	2.4	3.1	4.0
3.1. Metal debris	5.7	3.8	1.2	3.0	6.3	8.4
3.2. Glass debris	4.6	1.4	0.0	2.8	3.1	0
3.3. Rubber debris	3.4	9.1	29.2	3.0	3.1	4.0
3.4. Wooden debris	12.3	14.8	19.4	7.1	9.4	8.4
3.5. Textile debris	2.2	2.7	5.0	2.5	3.1	11.5

Supplementary Table 3: Demographic data of fishers who participated in the socioeconomic impact survey. Note on sample size representative with total N=199 fishers: a: Confidence Level-CL 90%, Margin of Error-ME 7%; b: CL 90%, ME 15%; c: CL 90%, ME 10%. Data are presented as mean (interquartile range-IQR of Q1–Q3). Respondents were predominantly male fishers in their 40s with secondary education or below and over 20 years of fishing experience. Most owned and captained their vessels, with fishing as their primary household income source.

Parameters	Sub-groups	Value	Unit
**Total answers (N)**		**199**	**answers (100%)**
By fishing gears	Trawl net	167	answers (83.9% [a])
	Gillnet	32	answers (16.1% [b])
By locations	Hai Phong	59	answers (29.6% [c])
	Nam Dinh	72	answers (36.2% [c])
	Ben Tre	68	answers (34.2% [c])
**Demographics**			
Role	Owner and captain	87.5	%
	Captain only	2.0	%
	Crew	4.5	%
	Others (wife, family member)	6.0	%
Gender	Male	90	%
	Female	10	%
Age		46 (IQR 39-52)	Years
Experience in fishing		21 (IQR 12-30)	Years
Highest education level at secondary schools or below		89.4	%
Household size		4 (IQR 3-5)	People
Number of dependents		2(IQR 1-2)	People
Household income	Total income	782 (IQR 589-1173)	USD/month
	Income from fishing	100% (IQR 75-100%)	% of total income
Main news sources	Smartphone and Internet	65	%
	TV and Radio	35	%

Supplementary Table 4: Frequency of encountering marine plastic debris. Data are based on estimates from interviewed nearshore fishers (N = 199). Values are presented as median (interquartile range IQR of Q1–Q3).

	**Ben Tre**	**Hai Phong**	**Nam Dinh**
**Trawlers (n=167)**	**51**	**55**	**61**
Depth (m)	5 (4-7)	10 (7-15)	14 (6-20)
Landing (kg/haul)	50 (30-100)	200 (96-500)	150 (80-300)
Mixed marine debris (kg/haul)	30 (10-50)	10 (4-23)	10 (5-29)
Plastic debris (kg/haul)	2 (1.5-3)	2 (1-5)	2.5 (1-5)
Propeller entanglement incidents (times/year)	4 (0-10)	1 (0-20)	0 (0-1)
Downtime due to plastic debris (hours/vessel/year)	161 (96-420)	107 (54-204)	163 (43-342)
Overall downtime for trawlers across locations (hours/vessel/year)	154 (60-320)		
**Gillnets (n=32)**	**17**	**4**	**11**
Depth (m)	6.5 (4-12.5)	10 (5-15)	18 (6-24)
Landing (kg/haul)	70 (50-150)	30 (30-150)	100 (50-300)
Mixed marine debris (kg/haul)	5 (3-14)	15 (3-27)	8 (6-50)
Plastic debris (kg/haul)	2 (1-4)	2 (0.5-4.5)	2 (1-3)
Propeller entanglement incidents (times/year)	0 (0-3)	7 (1-92)	0 (0-1)
Downtime due to plastic debris (hours/vessel/year)	135 (64-548)	242 (105-423)	432 (90-1080)
Overall downtime for gillnetter across locations (hours/vessel/year)	208 (67-569)		
Total downtime across gear types and locations (hours/vessel/year)	158 (62-360)		

Supplementary Table 5: Highest frequency answers in identifying plastic debris hotspots through local ecological knowledge. Names of reported hotspots are provided in Vietnamese to facilitate location identification.

Interviewees	Locations	Hotspot polluters	Hotspot months	Hotspot seasons	Hotspot locations	Hotspot items
Trawlers (N=167)	Nam Dinh	Upstream people	May-Oct	South wind season	Ba Lạt, Ninh Cơ, Quất Lâm river mouths, Sluice gate Cống No.9	Plastic bags, fabrics.
	Hai Phong	Fishers	May-Oct	Year-round	Hòn Dáu island, Văn Úc river mouth	Plastic bags, feed bags, fabrics.
	Ben Tre	Fishers	55% May-Oct and 45% Nov-Apr	South + East wind season	Cửa Đại, Ba Lai river mouthss	Plastic bags, buckets, ropes.
Gillnetters (N=32)	Nam Dinh	Fishers	May-Oct	Rain/storm, South wind season	Ba Lạt river mouth, Cồn Lu sandbar	Plastic bags, single-use plastics.
	Hai Phong	Fishers	May-Oct	Rain/storm season	Hòn Dáu island, Văn Úc river mouths	Plastic bags, plastic bottles.
	Ben Tre	Fishers	May-Oct	East wind season	Cửa Đại, Ba Lai river mouths	Plastic bags, single-use plastics.

Supplementary Table 6: Socioeconomic, geoeconomic, and fishery features of the three studied locations. Data sources:[3–5]

Groups	Main features	Unit	Hai Phong City	Nam Dinh Province	Ben Tre Province
Socioeconomic	Monthly income	USD/capita	250	215	149
	Gross regional domestic product (GRDP)	USD/capita	7,738	2,189	2,345
Plastic waste management	Plastic waste production rate	kg/capita/year	37	23	34
	Total plastic waste production	Tonnes/day	212	117	122
	Plastics leakage into water environment	Tonnes/year	1.50	1.80	5.40
Geoeconomic	Location		Northern Viet Nam (islands-coast). Red River Delta with estuary of Thai Binh River system. Coastal city with islands in Red River Delta region.	Northern Viet Nam (coast). Red River Delta with 4 estuaries of Red River and Day River system	Southern Viet Nam (coast). Mekong River Delta with 4 estuaries of Mekong River.
	Coastline	km	125	72	65
	Recognised assignations		UNESCO World Natural Heritage Site, MPA, national park in Cat Ba archipelago	Ramsar site, national park in Xuan Thuy	MSC certification for clam fishery in Ben Tre
Fisheries sector	Total fisheries yield	Tonnes/year	162,453	162,703	588,066
	From aquaculture	%	61	69	59
	From fishing	%	39	31	41
Fishing vessels	Total registered	vessels	890	1761	2766
	Coastal zone	%	38	54	31
	Inshore zone	%	27	16	14
	Offshore zone	%	35	30	55
	Total gillnetters	%	29	77	13
	Total trawlers	%	22	20	69
	Total fishing labours	people	4970	1761	-

Supplementary Table 7: Knowledge and attitude of fishers about ALDFG and marine plastic debris. Values are presented as median Likert-scale scores (1: strongly disagree; 2: disagree; 3: neutral; 4: agree; 5: strongly agree) in level of agreement to the statement, unless otherwise specified. The number of responses (n), out of 199 interviews, is reported for each statement. Differences among groups were tested using the Kruskal–Wallis test with tie adjustment; significance is indicated as *P < 0.05, **P < 0.01 and *P < 0.001. Relevant qualitative quotes are provided alongside questions to support the quantitative responses.

		**Quantitative data**						**Qualitative data**
		**Ben Tre**	**Hai Phong**	**Nam Dinh**	**MEDIAN**	**MODE**	**P-value**	**Relevant quotes**
**Themes**	**Question:**<br>**How much are you agree or disagree with the following sentence, on the scale of 1-5**<br>**(where 1: Strongly disagree; 2: Disagree; 3: Neutral; 4: Agree; 5: Strongly agree; Empty: No opinion/not answered):**<br>(In your opinion, ALDFG or marine plastic debris can: [sentence])							
Environmental impacts	Negatively impact the marine environment (n=198)	3.5**	5	4	4	5	0.003**	BT13: "plastic waste that I brought with me or the one that I trawled up from the sea, I throw it all to the sea, because there is no space on vessel... the amount is not much, not affecting the environment...the sea is vast"
	Take a long time to degrade (n=199)	5	5	5	5	5	0.090	ND114:"plastic even for hundreds years it will never degrade"
	Can injure or kill marine animals (n=196)	4	4	5	4	5	0.01**	ND117: "big fish can stuck in lost fishing net under the sea"
	Cause loss of habitat for lives below water (n=199)	4	4	4	4	5	0.408	ND107: "I think lost fishing gear can even be a place for baby fish to stay and breed, it is only dirty but not affect the habitat for fish"

		Quantitative data						Qualitative data
		Ben Tre	Hai Phong	Nam Dinh	MEDIAN	MODE	P-value	Relevant quotes
	Kill fish when they eat plastic waste (n=195)	3	1	3	3	3	<0.001***	HP73:"I don't know if fish eat plastic bags or not, less likely, they are smart"
Economic impacts	Negatively impact my income from fishing (n=198)	4	4	5	4	5	0.033*	HP93: "Before I only need 6-7 hauls to get 2 tons, now I need 20-25 hauls to get that. Before I need only 2 more crews, now I need 3 people to separate out fish and trash"
	Damage my fishing gear and vessel (n=197)	4	4	5	4	5	<0.001**	ND152: "if there is a lot of trash in my trawl net, it will tear faster, and consume a lot of fuel"
	Reduce my total catch (n=191)	5*	4*	5	4	5	0.029*	ND141: "now I have to pick shrimp and fish out of trash, not the other way around" HP102: "plastic in my trawl net will make it float from the sea bed and I cannot catch as much fish"
	Reduce the total fish stock (n=190)	4	4	5	4	5	0.054	BT43: "where there are a lot of plastic waste, there will be no shrimp and fish"
	Increase the risk at sea for me and my crew (n=190)	4	4	4	4	5	0.348	ND165: "here there are a few accidents that people died when jumped down to untangling rope stuck in propeller... Scratches from barnacles is very often, I got it too"

		Quantitative data						Qualitative data
		Ben Tre	Hai Phong	Nam Dinh	MEDIAN	MODE	P-value	Relevant quotes
Attitude towards interventions	Prevention: Fishers need to bring their trash back and stop littering (n=198)	4	5	5	4	5	0.017*	HP95: "If there is a program, I will volunteer to bring trash to shore immediately"
	Punishment: Fishers who litter will receive a fine. (n=194)	4	4	3	3	5	0.247	BT33: "Everyone must do it, if 1 person bring trash back and everyone else still throwing it down then there will be no difference"
	Incentivisation: Fishers who bring plastic waste back will receive monetary reward (n=196)	4.5	5	5	5 [a]	5	0.303	BT34: "It would be great if we can bring trash to shore, like in Singapore that I saw on TV. Money from selling trash will give to the crew members to encourage them"
	Monetary incentive to bring marine debris back to shore (USD/kg marine debris) (n=119)	0.12	0.25***	0.08	0.16 (IQR 0.08 – 0.23)	0.08	0.001*** [b]	HP102: "The buying price for marine plastic must higher than the current price on land, otherwise no one will bring back...just throw it back to the sea to continue another haul"
Current way of dealing with marine plastic debris	Frequency of fishers currently throwing back marine debris caught in their net to ocean (% of n=198)	79.4	69.5	78.9	77			
	Frequency of fishers currently only bring back valuable items back to shore (% of n=198)	10.3	22.0	16.9	18			

Notes: a: Frequency of choice 5 (Very agree) = 56% and Frequency of choice 4 (Agree) = 23%. b: Kruskal-Wallis test, H (df=1, n=119) = 19.9)

Supplementary Table 8: Socioeconomic impacts of marine plastic debris on fishing vessels in Viet Nam compared to previous research. Data are presented as mean ± standard deviation (otherwise specified) to compare with the existing study on socioeconomic impact in the literature.

**Sources**	**This study**	**This study**	Xuan et al. (2022)[6]	Raes et al. (2022)[7]	Llerena et al. (2025)[8]	Savels et al. (2022)[9]	Rodríguez et al. (2020)[10]	Mc Ilgorm et al. (2020)[11]	Deloite (2019)[12]
Data year	2023	2023	2020	2021	2022	2019	2017	2015	2018
Type of gear	Trawl net	Gillnet	Trawl net (65%) and Gillnet (28%)	Gillnet	Mixed	Trammel nets, gillnets, longline, traps	Non-specified professional fishers	Model	Model
Location	Viet Nam (Hai Phong, Nam Dinh, Ben Tre)	Viet Nam (Hai Phong, Nam Dinh, Ben Tre)	Viet Nam (Phu Yen, Khanh Hoa, Ninh Thuan)	Viet Nam (Ba Ria-Vung Tau)	Ecuador and Peru	Cyprus	Azores	Viet Nam	Viet Nam
Sample size (n)	167	32	249	15	1349	88	187	-	-
Proportion by mass of plastic debris in the total catch (% wet mass of total catch)	6.8 ± 6.7	9.0 ± 11.1	24 ± 23% (of mixed marine debris)	2	-	-	-	-	-
Downtime (minutes per incident)	22 ± 7	36 ± 26	-	11-19	-	20	Minutes to 60 days/incident	-	-
Direct cost to each fishing vessel (Mean ± Standard Deviation)	781 ± 938 USD/year	1315 ± 1100 USD/year	1364 ± 1649 USD/year	362 USD/year	-	167 EUR/incident	3502 ± 1750 USD/year (1618 EUR/incident)	-	-

Direct cost to each fishing vessel (Median (IQR))[a]	518 (188-1027) USD/year	1164 (576-1655) USD/year	-	-	-	-	-	-	-
Total cost as % of Gross Domestic Product (GDP)	-		-	-	0.71% and 0.84% fishery GDP of Peru and Ecuador in 2021	-	0.02% national GDP in 2016	0.08% national GDP in 2015	0.02% national GDP in 2018

Note: a: The costs to each fishing vessel across gear types (n=199 fishers) in this study (reported as (Median (IQR)): Total cost = 3421 (1848-6159) USD/year; Direct cost = 594 (222-1174) USD/year; Loss opportunity cost = 2816 (1418-4929) USD/year.

Supplementary Table 9: Results of the verification survey. The validation survey was conducted with additional fishers (n = 94) who did not participate in the initial survey and covered all fishing zones and major ports across the three study locations. Levels of agreement with the socioeconomic findings were coded into three categories based on respondents' answers: 0 (disagree), 1 (agree) and 2 (partially agree, where findings were considered applicable only to certain contexts or groups).

		Fishing zone of interviewees				Relevant quotes
	Details	Coastal	Inshore	Offshore	Total	
Total samples	N	26	38	30	94	
Agreement level to the result of socioeconomic impact assessment	Agree	80%	75%	75%	77%	- Yes, it is correct. I got [marine debris] stuck in propeller a lot. Even have [cases when] fish bit hands off [when fisher untangle propeller].<br>- The older the fishing vessels, the higher the impacts. [We] Cannot reduce the marine debris. The majority of people who go to work [fishing], throw it [trash] to the sea. They mock the one who bring trash back so no one will bring back trash.
	Partially agree	5%	10%	5%	7%	- Plastic debris has impacts on the fishing activities, but it depends on the gear type. My current gear type does not receive much impact.
	Not agree	15%	15%	20%	16%	- [There is] No impact. There were many trash [marine debris], but I throw it all back down [to the sea], so no problem.<br>- Because I fish in the offshore area, with little [marine] debris. I am not impacted directly, only indirectly.
Current way of dealing with plastic waste from vessels	Dump all to ocean	50.0%	42.1%	56.7%	48.4%	
	Bring back recyclables and dump the rest	26.9%	44.7%	40.0%	37.9%	
	Bring back all to shore	23.1%	13.2%	3.3%	12.6%	

Supplementary Table 10: List of prioritised locations to launch incentive programme to bring back marine plastic debris. The coastal and fishing communities located at the outlet of rivers with highest plastic leakage to ocean and lowest income should be prioritised. In this table, data of top 50 rivers contributing to global ocean plastic from Meijer et al. (2021)[13], average GDP per capita by country from World Bank[14], and average income by province by Viet Nam General Statistical Office[3]. Priority ranking is determined by combining two factors: (1) river plastic pollution rank (higher pollution = higher priority) and (2) income rank (lower income = higher priority). These two factors are weighted equally, based on the assumption that pollution level and income level have the same influence on selecting locations for prioritising incentive-based plastic collection systems. The factor for number of registered fishing vessels was excluded due to limitations in administrative data, which do not accurately reflect real-world conditions, as vessels frequently operate across fishing zones and provincial boundaries.

River name (Country)	Share of global plastics emitted to ocean (%) [13]	Average GDP by country (USD per capita)[14]	Rank of Highest River Plastic Pollution	Rank of Lowest Income	Com-bined Rank	Global Rank of Priority
Ulhas (India)	1.327265	2696.7	2	8	10	1
Ganges (India)	0.633003	2696.7	8	8	16	2
Lagos Harbour (Nigeria)	0.408389	806.9457	15	1	16	2
Pasig (Philippines)	6.432129	3984.8	1	18	19	4
Tullahan (Philippines)	1.327265	3984.8	2	18	20	5
Pazundaung Creek (Myamnar)	0.36755	1359.26	18	3	21	6
Meycauayan (Philippines)	1.225167	3984.8	5	18	23	7
Pampanga (Philippines)	0.949505	3984.8	6	18	24	8
Libmanan (Philippines)	0.724891	3984.8	7	18	25	9
Hugli (India)	0.398179	2696.7	17	8	25	9
Msimbazi River (Tanzania)	0.326711	1185.745	23	2	25	9
Rio Grande de Mindanao (Philippines)	0.541116	3984.8	9	18	27	12
Agno (Philippines)	0.469647	3984.8	10	18	28	13
Agusan (Philippines)	0.469647	3984.8	10	18	28	13
Bharathappuzha (India)	0.35734	2696.7	20	8	28	13
Paranaque (Philippines)	0.449228	3984.8	12	18	30	16
Iloilo (Philippines)	0.428809	3984.8	13	18	31	17
Wouri River (Cameroon)	0.285872	1762.368	26	5	31	17
Malad Creek (India)	0.275663	2696.7	29	8	37	19
Ebrie Lagoon / Komoe (Cote d'Ivoire)	0.336921	2709.895	21	17	38	20
Karnaphuli (Bangladesh)	0.265453	2593.416	32	7	39	21
Yangon River (Myamnar)	0.255243	1359.26	36	3	39	21
Imus (Philippines)	0.316502	3984.8	24	18	42	23
Panvel Creek (India)	0.255243	2696.7	36	8	44	24
Klang (Malaysia)	1.327265	11867.3	2	43	45	25
Zapote (Philippines)	0.275663	3984.8	29	18	47	26
Douala Estuary (Cameroon)	0.234824	1762.368	42	5	47	26
Cagayan de Oro (Philippines)	0.265453	3984.8	32	18	50	28
Davao River (Philippines)	0.265453	3984.8	32	18	50	28

Chilyar River (India)	0.234824	2696.7	42	8	50	28
Periyar River (India)	0.234824	2696.7	42	8	50	28
**Soai Rap (Vietnam)**	**0.418599**	**4717.3**	**14**	**38**	**52**	**32**
Malaking Tubig (Philippines)	0.255243	3984.8	36	18	54	33
Tambo, Pasay (Philippines)	0.255243	3984.8	36	18	54	33
Chao Phraya (Thailand)	0.408389	7345.1	15	40	55	35
Mithi River (India)	0.224614	2696.7	47	8	55	35
Cagayan River (Philippines)	0.234824	3984.8	42	18	60	37
Jalaur River (Philippines)	0.234824	3984.8	42	18	60	37
Sarawak (Malaysia)	0.336921	11867.26	21	43	64	39
Cilliwung (Indonesia)	0.285872	4925.43	26	39	65	40
Hamulauon (Philippines)	0.224614	3984.8	47	18	65	40
Huangpu (China)	0.36755	13303.15	18	48	66	42
Kelani (Sri Lanka)	0.275663	4515.568	29	37	66	42
Langat (Malaysia)	0.285872	11867.26	26	43	69	44
Chenzhen River (China)	0.296082	13303.15	25	48	73	45
Rio Pavuna (Brazil)	0.265453	10280.31	32	41	73	45
Kelantan River (Malaysia)	0.255243	11867.26	36	43	79	47
Zhujiang/Canton (China)	0.255243	13303.15	36	48	84	48
Rio Ozama (Dominican Republic)	0.224614	10875.66	47	42	89	49
Sungai Kuantan (Malaysia)	0.224614	11867.26	47	43	90	50

Provinces in Viet Nam	River name (alternative name)	Riverine plastic export to ocean (kg/year)[13]	Average monthly income per capita in 2024 by province (USD/capita)[15]	Rank of Highest plastic polluted rivers in Viet Nam	Rank of lowest monthly income	Viet Nam Rank of Priority
Hà Tĩnh	Sông Cả (Sông Lam)	792900	162.369	4	7	1
Quảng Nam	Sông Thu Bồn	590100	160.3665	8	5	2
Trà Vinh	Sông Hậu*	684000	167.0235	7	16	3
Quảng Ngãi	Sông Trà Khúc	425000	166.2741	15	12	4
Quảng Trị	Sông Thạch Hãn	200100	152.6827	32	2	5
Quảng Ngãi	Sông Trà Bồng	257700	166.2741	24	12	6
Thanh Hoá	Sông Mã	1035100	198.467	2	34	6
Phú Yên	Sông Đà Rằng	187200	154.9089	33	4	8
Cà Mau/Bạc Liêu	Sông Gành Hào	376700	174.1332	17	21	9
Tiền Giang	Sông Vàm Cỏ*	719000	196.4028	5	33	9
Nghệ An	Sông Lạch Vạn	324800	172.9131	20	19	11
Thanh Hoá	Sông Yên	575900	198.467	9	34	12
Bình Định	Sông Côn (Đầm Thị Nại)	328700	185.8688	19	26	13
Quảng Bình	Sông Gianh	180700	163.5366	35	10	13
Cà Mau	Sông Ông Đốc	251900	174.1332	25	21	15
Quảng Ngãi	Sông Ba Liên	157500	166.2741	38	12	16
Thừa Thiên Huế	Sông Hương	483400	200.6028	13	38	17

Thừa Thiên Huế	Sông Ô Lâu (Đầm Tam Giang)	483400	200.6028	13	38	17
Quảng Bình	Sông Nhật Lệ	141900	163.5366	41	10	17
Hà Tĩnh	Sông Rào Cái	128500	162.369	44	7	17
Hà Tĩnh	Sông Nghèn	128500	162.369	44	7	17
Quảng Trị	Sông Bến Hải	120100	152.6827	52	2	22
Ninh Thuận	Sông Dinh/Sông Cái Phan Rang	117400	135.71	54	1	23
Nghệ An	Sông Cấm	171000	172.9131	36	19	23
Bạc Liêu	Kênh Điền Hải	509300	202.25	11	45	25
Thanh Hoá/Ninh Bình	Sông Càn	272100	198.467	22	34	25
Nam Định	Sông Hồng (Ba Lạt)**	797100	231.7795	3	53	25
Bình Định	Đầm Thị Nại (Nam)	206000	185.8688	31	26	28
Kiên Giang	Sông Cái Lớn	361200	201.5721	18	41	29
Thái Bình	Sông Trà Lý**	494100	222.978	12	47	29
Quảng Ngãi	Sông Vệ	124000	166.2741	48	12	31
Hồ Chí Minh	Sông Soài Rạp	4127400	277.9632	1	61	32
Quảng Nam	Sông Trường Giang	106500	160.3665	59	5	33
Hải Phòng	Sông Văn Úc**	709600	275.1717	6	58	33
Bến Tre	Sông Hàm Luông*	122900	171.3715	49	17	35
Khánh Hoà	Sông Cái	137000	176.0972	42	24	35
Bình Định	Sông Lại Giang	150400	185.8688	40	26	35
Bình Thuận	Sông Cà Ty	165300	191.0847	37	30	38
Hải Phòng	Sông Thái Bình**	524500	275.1717	10	58	39
Bà Rịa-Vũng Tàu	Sông Thị Vải	302500	223.1298	21	48	40
Bình Định	Đề Gi	133200	185.8688	43	26	40
Kiên Giang	Kênh Linh Huỳnh (Hà Tiên-Rạch Giá)	217300	201.5721	29	41	42
Kiên Giang	Sông Giang Thạnh	216700	201.5721	30	41	43
Cà Mau	Kênh Bảy Hạp	121500	174.1332	50	21	43
Bạc Liêu	Sông Mỹ Thanh	235700	202.25	26	45	43
Bến Tre	Sông Ba Lai*	110000	171.3715	57	17	46
Ninh Bình	Sông Đáy	272100	230.8547	22	52	46
Hải Phòng	Sông Bạch Đằng**	387200	275.1717	16	58	46
Khánh Hoà	Sông Cầu Lam	120900	176.0972	51	24	49
Thừa Thiên Huế	Sông An Cựu (Đầm Cầu Hai)	152500	200.6028	39	38	50

Note: * Rivers in the Mekong River System; ** Rivers in the Red River System. We use the administrative boundary of provinces before the provincial consolidation in Viet Nam effective on 1 July 2025. We use the Vietnamese characters for name of provinces and rivers to avoid confusion and facilitate the location identification for readers.

Supplementary References

1. Walker, T. R., McGuinty, E. & Hickman, D. Marine debris database development using international best practices: A case study in Vietnam. *Mar. Pollut. Bull.* **173**, 112948 (2021).

2. GESAMP. *Guidelines for the Monitoring and Assessment of Plastic Litter in the Ocean*. *GESAMP* vol. R99 http://www.gesamp.org/publications/guidelines-for-the-monitoring-and-assessment-of-plastic-litter-in-the-ocean (2019).

3. General Statistical Office. Statistical yearbook. https://www.gso.gov.vn (2023).

4. WWF-Viet Nam. *Report on Plastic Waste Generation in 2022*. (Thanh Nien Publishing House, Hanoi, 2023).

5. Department of Fisheries. *Báo Cáo Từ Chi Cục Thủy Sản Tại Nam Định, Hải Phòng, Bến Tre [Reports from Department of Fisheries of Nam Dinh, Hai Phong and Ben Tre]*. (2023).

6. Xuan, B. B., Ngoc, Q. T. K. & Börger, T. Fisher preferences for marine litter interventions in Vietnam. *Ecological Economics* **200**, (2022).

7. Raes, L., Jain, A., Ba, T. N. & Savels, R. The economic impact of marine plastics, including ghost fishing, on fishing boats in Phước Tinh and Loc An, Ba Ria Vung Tau Province, Viet Nam. https://biblio.ugent.be/publication/8764101/file/8764102 (2022).

8. Llerena, P. D. *et al.* Assessing economic losses in artisanal fisheries from marine plastic pollution in coastal Ecuador and Peru. *Mar. Policy* **173**, (2025).

9. Savels, R., Raes, L., Papageorgiou, M. & Speelman, S. Economic assessment of abandoned, lost and otherwise discarded fishing gear (ALDFG) in the fishery sector of the Republic of Cyprus. https://biblio.ugent.be/publication/8757113/file/8757116 (2022).

10. Rodríguez, Y., Ressurreição, A. & Pham, C. K. Socio-economic impacts of marine litter for remote oceanic islands: The case of the Azores. *Mar. Pollut. Bull.* **160**, 111631 (2020).

11. McIlgorm, A., Raubenheimer, K. & McIlgorm, D. E. Update of 2009 Apec Report on Economic Costs of Marine Debris To Apec Economies. *APEC Oceans and Fisheries Working Group, A report to the APEC Ocean and Fisheries Working Group by the Australian National Centre for Ocean Resources and Security (ANCORS)* 79 (2020).

12. Deloitte. *The Price Tag of Plastic Pollution - An Economic Assessment of River Plastic*. *Deloitte* https://www2.deloitte.com/content/dam/Deloitte/nl/Documents/strategy-analytics-and-ma/deloitte-nl-strategy-analytics-and-ma-the-price-tag-of-plastic-pollution.pdf (2019).

13. Meijer, L. J. J., van Emmerik, T., van der Ent, R., Schmidt, C. & Lebreton, L. More than 1000 rivers account for 80% of global riverine plastic emissions into the ocean. *Sci. Adv.* **7**, (2021).

14. World Bank. GDP per capita by country. https://data.worldbank.org/indicator/NY.GDP.PCAP.CD.

Supplementary Methods

Interviews and Informed Consent Form

Mã phiếu:

PHIẾU THU THẬP THÔNG TIN HỘ KHAI THÁC THỦY SẢN
DỰ ÁN "Nguồn phát thải, nơi tích tụ và các giải pháp nhằm giảm thiểu tác động của rác thải nhựa đến cộng đồng ven biển ở Việt Nam"

Mục tiêu của dự án nhằm hiểu rõ hơn về **nguồn** rác thải nhựa đại dương và **đánh giá tác động** của ô nhiễm rác thải nhựa đến các hoạt động **kinh tế xã hội**, chất lượng **môi trường**, hệ **sinh thái** và **sức khoẻ** con người; từ đó đề xuất **chính sách và giải pháp** nhằm giảm thiểu tác động của rác thải nhựa đến cộng đồng ven biển tại Việt Nam.

(Ghi chú: Trước khi bắt đầu, cần cung cấp thông tin cho người được phỏng vấn về dự án và các quyền lợi của họ khi tham gia phỏng vấn và ghi lại sự đồng ý của người tham gia (bằng ghi âm hoặc ký tên))

- Tôi hiểu sự tham gia khảo sát/nghiên cứu này là tự nguyện và tôi có thể từ chối trả lời bất cứ câu hỏi nào và có thể rút khỏi nghiên cứu bất cứ lúc nào, không cần cung cấp lý do.
- Tôi hiểu khảo sát này không thu thập các thông tin cá nhân và các câu trả lời tôi đưa ra sẽ ở dạng ẩn danh trong tất cả các kết quả nghiên cứu và các thông tin tôi cung cấp sẽ chỉ được sử dụng cho mục đích nghiên cứu, xuất bản các bài báo, báo cáo và bản tin.

☐ Tôi hiểu và đồng ý tham gia. Ký tên:...

Tên tỉnh: ……….................……; Tên huyện: ………..…......…; Tên xã: ………….....……
Cảng cá: ………………………….

Phần 1: Thông tin chung

<table>
<tr><td colspan="2">Thông tin về hoạt động khai thác của tàu</td></tr>
<tr><td colspan="2">Q1. Nhóm chiều dài tàu: ☐<12m (1) ☐ 12-15m (2) ☐ >15m (3)
Q2. Số thuyền viên bình quân trên tàu:………., số người làm phân loại cá:………………………</td></tr>
<tr><td rowspan="8">Q3. Nghề khai thác chính:
☐ Lưới kéo (1)
☐ Lưới rê (2)
☐ Khác: ……………</td><td>Q3.1. Số tháng hoạt động trung bình/năm: ……………………...</td></tr>
<tr><td>Q3.2. Số chuyến biển trung bình/tháng:……………………..…..</td></tr>
<tr><td>Q3.3. Số ngày trung bình/chuyến: ……………………………..</td></tr>
<tr><td>Q3.4 Số mẻ lưới/ngày: ………………... mẻ</td></tr>
<tr><td>Q3.5 Số giờ đánh bắt TB/mẻ: …………......... giờ</td></tr>
<tr><td>Q3.6 Kích thước lưới sử dụng: ……………………………………..
…………………………………………………………………</td></tr>
<tr><td>Q3.7 Độ sâu thả lưới: ………… ……………..……………..mét</td></tr>
<tr><td>Q3.8 Ngư trường:…………………………………………………</td></tr>
</table>

Phần 2: Sản lượng và xu hướng sản lượng qua thời gian của hoạt động khai thác.

Q4. Sản lượng đánh bắt TB/chuyến biển (kg):……………………………………………...

Q5. So với 5 năm trước đây sản lượng đánh bắt có giảm hay không?

☐ Có (1) ☐ Không (0)

Q6. Nếu **Có**, nguyên nhân của việc giảm sản lượng đánh bắt là gì? (có thể chọn nhiều đáp án)

☐ Có quá nhiều tàu khai thác (1)

☐ Do có những thay đổi trong quy định về hoạt động đánh bắt (2)

☐ Do thay đổi về khí hậu, thời tiết (3)

☐ Do một số lượng cá đã bị mắc vào các ngư lưới cụ bị mất hoặc vứt lại trên biển (4)

☐ Khác (5): ……………………………………………………………………………...

Phần 3: Phân loại và nguồn gốc rác thải nhựa thường gặp

Q7. Trong mỗi mẻ lưới đánh bắt của tàu thường có rác thải nhựa hay không?<br>☐ Không có (0%) (1)<br>☐ Hiếm khi (10-20%) (2)<br>☐ Thỉnh thoảng (30-50%) (3)<br>☐ Thường xuyên (50-80%) (4)<br>☐ Rất thường xuyên (gần như mẻ nào cũng có, >80%) (5)
Q8. Khối lượng **rác thải nhựa** trung bình trong sản lượng của **mỗi mẻ lưới** là (kg/mẻ): *Ghi chú tổng lượng rác biển và thành phần nhựa*<br>…………………………………………………………………………………………
Q9. Thời gian hay gặp rác thải nhựa nhất trong mỗi mẻ lưới trong năm (ghi rõ âm lịch hay dương lịch): …………………………………………………………………………………
Q10. Khu vực hay gặp phải rác thải nhựa nhất: ☐ Vùng bờ; ☐ Vùng lộng; ☐ Vùng khơi<br>Cụ thể khu vực nào: … ………………………………………………………………………

Q11. Theo ông/bà, ai là nguồn phát thải rác thải nhựa chính ở khu vực của ông/bà? – Sắp xếp theo thứ tự từ nhiều đến ít.

…………………………………………………………………………………………

…………………………………………………………………………………………

…………………………………………………………………………………………

…………………………………………………………………………………………

…………………………………………………………………………………………

(Gợi ý: Các hộ NTTS khác; Tàu cá; Tàu du lịch; Người dân địa phương; Người dân địa phương khác; Khách du lịch; Nhà hàng; Khách sạn;….)

Mã phiếu:

Q12. Anh/chị cho biết số lượng rác thải nhựa trung bình trong **mỗi mẻ lưới** phân theo **loại** ở bảng dưới đây:

**Phân loại**	**Không có (1)**<br>**0%**	**Có ít (2)**<br>**1-5%**	**Vừa phải (3)**<br>**5-10%**	**Nhiều (4)**<br>**10-20%**	**Rất nhiều (5)**<br>**Hơn 20%**
Túi nilion (1)					
Sản phẩm nhựa dùng 1 lần (2)					
Chai nhựa (3)					
Dây câu, lưới, dây giềng (4)					
Khác (5) (liệt kê cụ thể)					

Q13. Cách xử lý lượng rác thải nhựa trong mỗi mẻ lưới………………….......……………………

………………………………………………………………………………………………

Q14. Lượng rác nhựa trong mẻ đánh cá so với 5 năm trước đây?

☐ Tăng lên ☐ Không đổi ☐ Giảm đi

Q15. Anh/chị cho biết số lượng các sản phẩm nhựa dùng 1 lần mang theo mỗi chuyến đi và cách xử lý:

**Sản phẩm nhựa**	**Số lượng mang theo mỗi chuyến biển**<br>(Số lượng và đơn vị)	**Cách xử lý**<br>*Vứt xuống biển (1)*<br>*Mang về bờ vứt (2)*<br>*Tái sử dụng (3)*<br>*Mang về bán tái chế (4)*
Túi nilion		
Chai nước nhỏ (0.5-1.5L)		
Bình nước 20L		
Mì gói		
Bao tải		
Khác:		

Phần 4: Các tác động của rác thải biển

Q16. Anh/chị cho biết **mức độ gặp phải các rủi ro** do rác thải biển gây ra đối với hoạt động khai thác:

	**Mức độ gặp phải rủi ro**					**Các thiệt hại**				
	**Không có (1)**	**Hiếm khi (2)**	**Thỉnh thoảng (3)**	**Thường xuyên (4)**	**Rất thường xuyên (5)**	**Không có (1)**	**Rất ít (2)**	**Vừa phải (3)**	**Nhiều (4)**	**Rất nhiều (5)**
Sản phẩm đánh bắt bị hư hỏng (1)										
Tốn thời gian cho việc xử lý rác (2)										
Ngư lưới cụ bị hư hỏng (3)										
Hệ thống chân vịt bị hư hỏng (4)										
Vỏ tàu bị hư hỏng (5)										
Mất ngư lưới cụ (6)										
Khác: …… (7)										

Q17. Thời gian để xử lý khi gặp rác thải nhựa

	**Thời gian**
- Thời gian gỡ rác nhựa khỏi ngư lưới cụ đánh bắt (1)	……………giờ/mẻ
- Thời gian nhặt rác nhựa ra khỏi phần sản lượng đánh bắt (2)	……………giờ/mẻ
- Thời gian sửa chữa ngư lưới cụ ngay trên biển (3)	……………giờ/lần
- Khác (4)	

Q18. Các thiệt hai do gặp phải rác thải nhựa **trong năm qua**.

**Phân loại**	**Chí phí/năm (triệu đồng)**
- Mua sắm ngư lưới cụ mới thay cho ngư lưới cụ bị mất hoặc hỏng do rác gây ra (1)	
- Chi phí sửa chữa ngư lưới cụ do rác gây ra (2)	
- Chi phí sửa chữa tàu do rác gây ra (3)	
- Chi phí khác (4)	
**Tổng** thiệt hại do rác gây ra năm vừa qua	

Q19. Cách thức mà anh/ chị thực hiện để đối phó với rác thải nhựa?

**Cách thức đối phó**	**Chọn cách thức** (có thể chọn nhiều hơn 1 phương án)	**Ước tính chi phí** (triệu đồng)
Chuyển đổi ngư trường khai thác (1)		
Áp dụng công nghệ/ kỹ thuật mới cho tàu nhằm tránh né rác thải (2)		
Thay đổi nghề khai thác (3)		
Mua bảo hiểm tàu (4)		
Đánh dấu khu vực có rác để tránh cho những lần sau (5)		
Liên kết với các ngư dân khác để cùng chia sẻ thông tin về khu vực có rác (6)		
Sử dụng lưới bằng vật liệu có thể phân hủy sinh học (7)		
Cách khác (8)………….. …………………………………		
Không ứng phó (9)		

Phần 5: Các tác động của rác thải nhựa đại dương

Q20. Anh/chị cho biết ý kiến của anh chị đối với các phát biểu sau:

**Phát biểu**	**Mức độ đồng ý**				
	Hoàn toàn không đồng ý **1**	Nói chung không đồng ý **2**	Trung dung **3**	Nói chung đồng ý **4**	Hoàn toàn đồng ý **5**
1. Ngư lưới cụ bị mất hoặc bị vứt lại trên biển gây ảnh hưởng xấu đến môi trường biển					
2. Ngư lưới cụ còn lại trên biển sẽ mất nhiều thời gian để phân hủy					
3. Ngư lưới cụ bị mất hoặc bị vứt lại trên biển sẽ phá hủy nơi cư trú và sinh sản của cá					
4. Ngư lưới cụ bị mất hoặc vứt lại trên biển sẽ tiếp tục bắt cá và các loài sinh vật biển khác					
5. Ngư lưới cụ bị mất hoặc bị vứt lại trên biển gây thiệt hại về kinh tế cho hoạt động đánh bắt của tôi					
6. Ngư lưới cụ bị mất hoặc bị vứt lại trên biển làm hỏng hóc các thiết bị và ngư lưới cụ của tàu của tôi					

7. Ngư lưới cụ bị mất hoặc bị vứt lại trên biển làm giảm sản lượng đánh bắt của tôi					
8. RTN chìm dưới đáy biển làm mất bãi đẻ của thủy sản bố mẹ, mất nơi sinh sống của con non					
9. RTN chìm dưới đáy biển ảnh hưởng đến nơi sinh sống của thủy sản, môi trường biển					
10. Thủy sản bị chết khi ăn phải RTN					
11. RTN biển làm giảm trữ lượng thủy sản, ảnh hưởng nghiêm trọng đến sản lượng khai thác thủy sản					
12. Rác thải nhựa biển gây rất nhiều rủi ro cho hoạt động khai thác thủy sản và thuyền viên					
13. Ngư dân cần mang rác thải nhựa sinh hoạt của họ về bờ để xử lý, không thải ra biển					
14. Cần có quy định cử phạt như dân không mang rác thải do mình thải vào bờ					
15. Ngư dân sẽ mang rác thải nhựa từ biển về bờ khi được trả một khoản phí xứng đáng					
16. Mức phí mong muốn để mang RTN từ biển về bờ:…………… …………….ngàn đồng/kg					

Phần 7: Thông tin người trả lời phỏng vấn

**Q21. Thông tin nhân khẩu học**	
1. Giới tính:<br>☐ Nam (1); ☐ Nữ (0)<br>☐ Không tiết lộ	5. Vị trí của người trả lời: ☐ Chủ tàu ☐ Thuyền trưởng<br>☐ Thuyền viên ☐ Khác: …<br>6. Số năm đã tham gia hoạt động khai thác: ……… năm<br>7. Số thành viên trong gia đình: ………….. người,<br>Trong đó: Số người phụ thuộc:……………… người
2. Tuổi người được phỏng vấn:<br>……………<br><br>3. Dân tộc:<br><br>Q4. Trình độ học vấn:<br>☐ Không có<br>☐ Cấp 1 (1)<br>☐ Cấp 2 (2)<br>☐ Cấp 3 (3)<br>☐ Trung cấp (4)<br>☐ Cao đẳng, Đại học (5)	8. Phương tiện thông tin **chính** để cập nhật tin tức:<br>☐ Radio<br>☐ Tivi<br>☐ Mạng xã hội (facebook, tiktok,..)<br>☐ Báo điện tử<br>☐ Báo giấy<br>☐ Khác:……………………………………………

☐ Khác:	

Q22. Doanh thu và chi phí của hoạt động khai thác

1. Thu nhập bình quân hộ gia đình/tháng (triệu đồng):…………………………………………..

Thu nhập bình quân từ **hoạt động khai thác** thủy sản/ tháng (triệu đồng): ……………………

2. Doanh thu trung bình/ chuyến (triệu đồng): ……………………………………………….

3. Chi phí TB/chuyến biển (triệu đồng):…………………………………………………….

Trong đó: Chi phí dầu: ………….. ……………………… triệu đồng

4. Cách thức phân chia thu nhập với thuyền viên:

☐ 50% - 50%(1) ☐ 60% - 40%(2) ☐ Khác (3):…

Xin cảm ơn ông/bà!

Người phỏng vấn **Người cung cấp thông tin**

Form ID:

INTERVIEW FORM FOR FISHING HOUSEHOLDS
PROJECT "Sources, Sinks and Solutions for Impacts of Plastics on Coastal Communities in Viet Nam" (3SIP2C)

The objective of the project is to better understand **the sources** of plastic debris found in the ocean and **assess the impacts** of plastic pollution on **socio-economic** activities, **environmental** quality, **ecosystem** and human **health** as a basis to propose **policies and solutions** to minimize the impacts of plastics on coastal communities in Vietnam.

(Note: Before starting an interview, an introduction on the project and the interviewee's rights should be provided and the interviewee's consent should be documented (orally or in writing).

- I understand that my participation in this survey/research is voluntary and that I may refuse to answer any questions and can ask to withdraw from the research at any time without giving a reason.
- I understand this survey does not collect my personal identifiable information and my responses will remain anonymous in all research findings and the information given will be used only for the purpose of research, publication of articles, reporting and newsletters.

☐ I understand and agree to participate in the research. Signature:.........................

Province: ……………; District: ……………………..; Commune: ……………………..
Fish harbor: ……………………

Part 1: General information

**Information about vessel operations**	
Q1. Vessel length: ☐<12m (1) ☐ 12-15m (2) ☐ >15m (3)<br>Q2. Average number of crew members on board:……………., including ………… persons sorting fish.	
Q3. Main fishing methods:<br>☐ Trawling (1)<br>☐ Gillnetting (2)<br>☐ Other: ……………	Q3.1. Average number of active months/year: ……………………...
	Q3.2. Average number of trips/month:……………………..
	Q3.3. Average number of days/trip: ……………………..
	Q3.4 Number of haul/day: ………….. hauls
	Q3.5 Average fishing hours/haul: …………......... hours
	Q3.6 Size of fish nets used: ………………………………….. ……………………………………………………………………
	Q3.7 Water depth in fishing locations: …………….meters
	Q3.8 Fishing grounds:…………………

Part 2: Output and output patterns.

Q4. Average catch/fishing trip (kg): ……………………..

Q5. Compared to 5 years ago, have the catches decreased?

☐ Yes (1) ☐ No (0)

Q6. If **Yes**, what is the cause of the decrease in catches? (You may choose multiple answers)

☐ Too many fishing vessels (1)

☐ Due to changes in fishing regulations (2)

☐ Due to changes in climatic and weather conditions (3)

☐ Due to a certain volume of fish stuck in fishing gear lost or abandoned at sea (4)

☐ Other (5): …………………………………………………………………………...

Part 3: Classification and origin of common plastics

Q7. How often plastics are found in each catch?<br>☐ None (0%) (1)<br>☐ Rarely (10-20%) (2)<br>☐ Occasionally (30-50%) (3)<br>☐ Often (50-80%) (4)<br>☐ Very often (almost found in every catch, >80%) (5)
Q8. Average volume of **plastics in each** catch (kg/catch): *Note on total marine debris and plastic debris*<br>…………………………………………………………………………………………
Q9. Time in a year when plastics is most found in each catch (with indication of either lunar or solar calendar):<br>………………………………………………………………………………………
Q10. Areas where plastics are most found: ☐Coastal areas;☐ Inshore areas; ☐ Offshore areas<br>In which locations specifically: …………………………………………….

Q11. In your opinion, what are the main sources of plastic debris in your neighborhood? - Name them in order of significance.

…………………………………………………………………………………………

…………………………………………………………………………………………

…………………………………………………………………………………………

(Suggestions if no answer: Other aquaculture households; Fishing vessels; Cruise ships; Local people; Other local people; Tourists; Restaurants; Hotels;….)

Form ID:

Q12. Please indicate the average amount of plastics in **each catch** by **category** in the table below:

**Classification**	**None (1) 0%**	**Few (2) 1-5%**	**Moderate (3) 5-10%**	**Many (4) 10-20%**	**Too many (5) Over 20%**
Nylon bags (1)					
Single-use plastic products (2)					
Plastic bottles (3)					
Fishing nets, rope (4)					
Other (5) (please specify)					

Q13. How did you deal with plastics in each catch?……………………..

………………………………………………………………………………………………

Q14. The amount of plastics in each catch compared to 5 years ago?

☐ Increasing ☐ Remaining the same ☐ Decreasing

Q15. The quantity of single-use plastic products brought along on every trip and how are they treated after use:

**Plastic products**	**Quantity of products brought along on fishing trips** (Quantity and unit)	**Forms of disposal** *Thrown into the sea (1)* *Brought back and thrown away onshore (2)* *Reused (3)* *Brought home and sold for recycling (4)*
Nylon bags		
Small water bottles (0.5-1.5L)		
20L water bottles		
Noodles		
Woven PP Sacks		
Other:		

Part 4: Impacts of marine debris

Q16. Please indicate **the level of risks caused by marine debris** to fishing operations:

	**Level of risks**					**Damages**				
	**None (1)**	**Rarely (2)**	**Occasionally (3)**	**Often (4)**	**Very often (5)**	**None (1)**	**Few (2)**	**Moderate (3)**	**Many (4)**	**Too many (5)**
Reduce quality of catches/fish(1)										
Time lost to deal with marine debris (2)										
Damaged fishing gears (3)										
Damaged propellers (4)										
Damaged hull (5)										
Loss of fishing gears (6)										
Other: …… (7)										

Q17. Time required to handle issues caused by plastics

	**Time**
- Time to remove plastics from fishing nets (1)	………………..hour(s)/haul
- Time to remove plastics from caught fish (2)	……………….hour(s)/haul
- Time to repair fishing gears at sea (3)	……………….hours/repair
- Other (4)	

Q18. Damage caused by plastics **in the past year**.

**Classification**	**Annual expenses (Million VND)**
- Buy new fishing gears to replace fishing gears lost or damaged due to plastic debris (1)	
- Repair of fishing gears for damage caused by plastic debris (2)	
- Vessel repair for damage caused by plastic debris (3)	
- Other (4)	
**Total** damage caused by plastic debris last year	

Q19. What actions have been taken to deal with plastics?

Actions	**Mark selected actions** (multiple choices)	**Estimated cost** (VND million)
Change fishing grounds (1)		
Application of new technologies on vessels to avoid debris (2)		
Change in fishing methods (3)		
Procurement of vessel insurance (4)		
Demarcation of areas where debris is often found and where future fishing should be avoided (5)		
Connection with other fishermen to share information about areas where debris is often found (6)		
Use of biodegradable fishing nets (7)		
Other (8)………….. ………………………………		
None action (9)		

Part 5: Impacts of ocean plastics

Q20. Please indicate what you think on the following statements:

**Statements**	**Level of agreement**				
	Strongly disagree **1**	Generally disagree **2**	Undecided/ Neutral **3**	Generally agree **4**	Strongly agree **5**
1. Fishing gear lost or abandoned at sea causes adverse effects on the marine environment					
2. Fishing gear left at sea will take a long time to decompose					
3. Fishing gear lost or abandoned at sea will destroy the habitat and breeding of fish					
4. Fishing gear lost or left at sea will continue to trap fish and other marine life					
5. Fishing gear lost or abandoned at sea causes economic damage to my fishing operations					

6. Fishing gear lost or abandoned at sea damages my vessel's fishing gear and equipment					
7. Fishing gear lost or abandoned at sea reduces my catch					
8. Plastics on ocean floor narrow spawning ground of the parent fish and the habitat of the baby fish					
9. Plastics on ocean floor affect marine environment					
10. Marine animals can die after eating plastic waste					
11. Marine plastics decrease fisheries' resources and seriously affect fishing output					
12. Marine plastics poses many risks to fishing operations and crew members					
13. Fishermen need to bring their plastics back home for disposal without throwing them into the sea					
14. There should be legal penalties on fishermen who fail to bring home their plastic waste					
15. Fishermen will bring home their plastic waste if there is a fair payment to them					
16. Expected payment to bring home their plastic waste:………………….thousands VND/kg					

Part 7: Interviewee information

Q21. Demographic information	
1. Gender:<br>☐ Male (1); ☐ Female (0)<br>☐Undisclosed	5. Respondent's status: ☐ Vessel owner ☐ Captain<br>☐ Crew ☐ Other: …<br>6. Number of years engaged in fishing operations: ……… year(s)<br>7. Number of family members: ………….persons, including: ....dependents
2. Age: ………….	8. **Main** sources of information:

3. Ethnic minority:	☐ Radio<br>☐ Television<br>☐ Social networks (facebook, tiktok,..)<br>☐ Online newspapers<br>☐ Print newspapers<br>☐ Other:…………………………………………
Q4. Level of education:<br>☐ None<br>☐ Primary (1)<br>☐ Lower secondary (2)<br>☐ Upper secondary (3)<br>☐ Associate (4)<br>☐ College, University (5)<br>☐Other:	

Q22. Fishing income and costs

1. Average monthly household income (million VND):……………………………..….

Average monthly income from **fishing operations** (million VND): ……………………

2. Average income/trip (million VND): …………………………………………………

3. Average cost/trip (million VND):………………………………………………….

including: ………….. …………………….. million VND for fuel.

4. How is the income shared with crew members:

☐ 50% - 50%(1) ☐ 60% - 40%(2) ☐ Other (3):…

Thank you for your participation!

Interviewer **Respondent**

Câu hỏi cho khảo sát ngư dân về tác động kinh tế của rác thải nhựa đại dương

(Ghi chú: Các câu hỏi dưới đây là một phần của một phiếu phỏng vấn, chỉ các câu hỏi liên quan đến nghiên cứu này được trình bày dưới đây)

Phần 1: Phiếu đồng ý tham gia và thông tin dự án.

Phần 2: Thông tin nhân khẩu học:

- Vai trò trên tàu cá:
- Nghề đánh bắt chính:
- Vùng đánh bắt chính:
- Địa điểm khảo sát:
- Cảng cá:

Phần 3: Ý kiến cá nhân:

- Theo anh/chị, khu vực biển/ven biển nơi mình đang đánh bắt có bị ô nhiễm rác thải nhựa không?
- Anh/chị có biết về rác thải nhựa và tác động của chúng không?
- Anh/chị có biết về vi nhựa và tác động của chúng không?
- Hiện nay, anh/chị có đang mang rác phát sinh trên tàu về bờ để thu gom xử lý không?
- Sau khi biết về thông tin sau về tác động của rác thải nhựa đến thu nhập của các tàu cá, anh/chị có thay đổi gì với các câu trả lời phía trên không? Anh/chị có ý kiến gì về kết quả nghiên cứu này?

Questions for the validation interview survey

(Note: The questions below are part of an interview questionnaire; only relevant questions to this study are listed below.)

Section 1: Informed consent form and agreement.

Section 2: Demographics:

- Role on fishing vessel:
- Main fishing gear/method:
- Main fishing zone:
- Location of interview:
- Fishing port of interview:

Section 3: Your current opinions:

- In your opinion, is the marine/coastal area where you fish affected by plastic pollution?
- Do you know about plastic debris and their impact?
- Do you know about microplastic debris and their impact?
- Do you currently bring plastic waste generated on board back to shore for collection and treatment?
  - After being provided with information on the impacts of plastic pollution on fishing vessel income, would you like to revise your previous responses? What are your thoughts on these research findings?

INFORMED CONSENT FORM

Project Title: Sources, Sinks and Solutions for the Impact of Plastic on Coastal Communities in Viet Nam (3SIP2C)

Before you agree to participate, make sure you have read and understood the Research Information Sheet and this Consent Form. Then, make sure you mark each box with 'X' to provide your consent and sign below. If you are under 18 years old, you will need to obtain your parent's or legal guardian's signature on this form as well as your own.

If you have any questions or concerns about this form, you should talk to someone you feel comfortable with and feel free to contact the research team through 3sip2c.project@gmail.com

You can also visit our Facebook page – https://www.facebook.com/Sources-Sinks-and-Solutions-for-Impacts-of-Plastics-on-Coast-of-Vietnam-103983809012907/ or our website www.3sip2c.com where you can find information about our research.

If you are happy to participate in the research, please initial each box as appropriate and then sign this form at the end. Leave blank any box for which you prefer not to give consent:

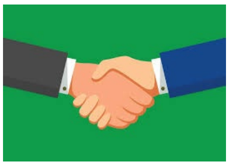

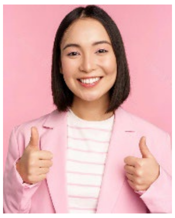

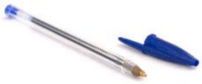

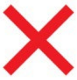

<table>
<tr><td colspan="2">1. The Researcher has shown/given me a copy of the Participant Information Sheet and I have had the opportunity to read and consider the information.</td><td>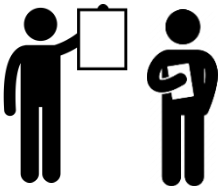</td><td></td></tr>
<tr><td colspan="2">2. I have been given the opportunity to ask any further questions and have had these questions answered to my satisfaction.</td><td>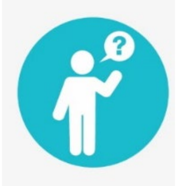</td><td></td></tr>
<tr><td colspan="2">3. I understand that my taking part is voluntary.</td><td>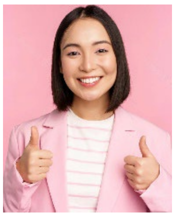</td><td></td></tr>
<tr><td rowspan="3">4. I understand that participating in the research activity involves:</td><td>A) Answering questions either in a questionnaire or interview</td><td>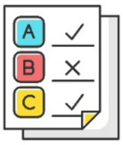

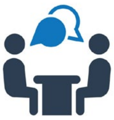</td><td rowspan="3"></td></tr>
<tr><td>B) Participating in a group discussion where I will talk with other Vietnamese coastal people, researchers, and governmental and non-governmental organisations’ workers</td><td>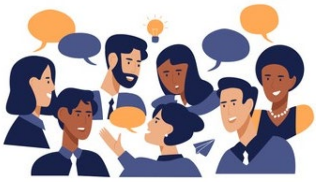</td></tr>
<tr><td>C) Participating in a group discussion where I will talk with other Vietnamese coastal people, researchers, and governmental and non-governmental organisations’ workers</td><td>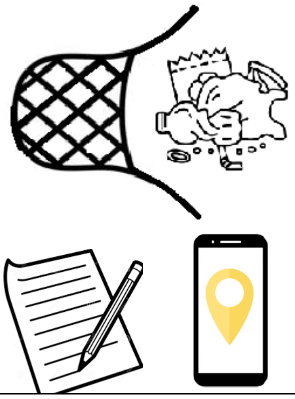</td></tr>
<tr><td colspan="2">5. I understand that the research activity may bee audio/video recorded.</td><td>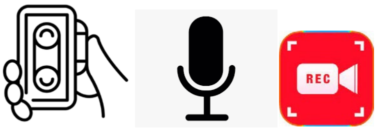
</td><td></td></tr>
</table>

6. I understand that all the personal information gathered about me, such as my name and address will be kept strictly confidential by the research team and will not be included in any public reports.	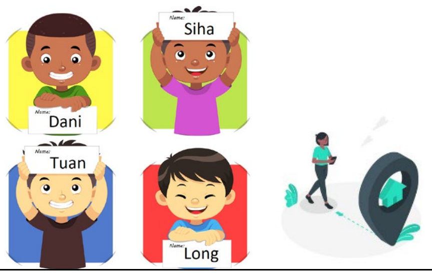	
7. I consent to be contacted by the research team for future research activities on the same project.	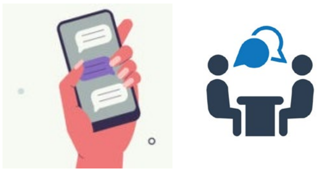	
8. I understand that my name and any information that can identify me will be removed to keep my identity anonymous	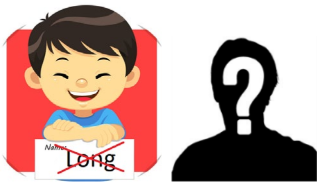	
9. I was informed and understood how my data and recordings will be stored and used during and after the end of the research.	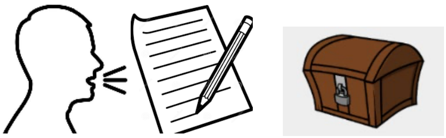	
10. I give my consent for my recordings to be reproduced for non-commercial purposes in reports, presentations, publications, websites and exhibitions connected to the research project.	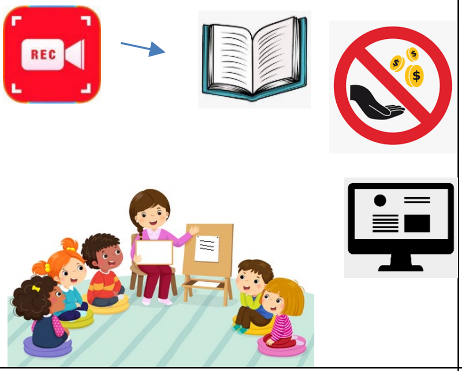	
11. I understand that my words may be quoted in academic publications, articles, books, reports, the project website, and other related project outputs that will be used only for non-commercial purposes.	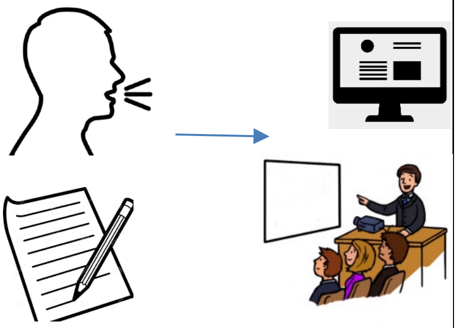	
12. I agree for the anonymised data I provide to be retained in secure storage for their future use on similar and related projects.	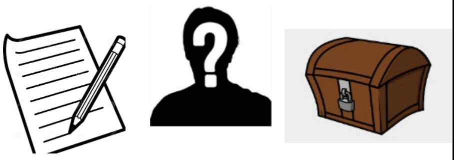	

13. I understand that this Informed Consent Form will be kept by the research team for a period of 3 years.		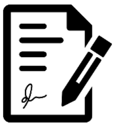 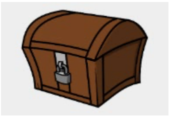	☐
14. I understand that I can ask to:	A) Withdraw from the research project	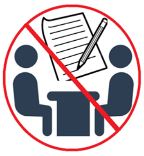 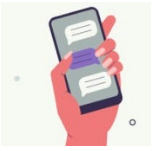	☐
	B) Withdraw my approval to use the recordings	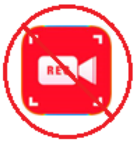 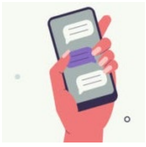	
	C) Remove specific quotes from research outputs	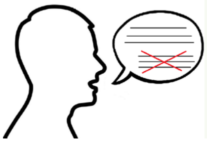 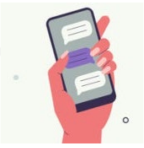	
15. To withdraw from the research I can contact the research team through 3sip2c.project@gmail.com		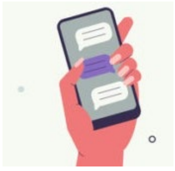	☐

I [name]____agree to take part in this research project.

Signature: ____ Date: ______________________

Name of Parent/Legal guardian (if under 18): ____

Signature: ____ Date: ______________________

Name of Researcher taking consent: ____

Signature: ____ Date: ______________________

PARTICIPANT INFORMATION SHEET

PROJECT TITLE
Sources, Sinks and Solutions for the Impact of Plastic on Coastal Communities in Viet Nam (3SIP2C)

INVITATION

You are invited to take part in a research project. Before you decide whether to take part, it is important to understand why the research is conducted and what it will involve. Please take time to read the following information carefully. Ask questions if anything is not clear or if you would like more information.

WHAT IS THE PURPOSE OF THE PROJECT?

Our project examines the plastic waste that enters the rivers and coasts of Vietnam. We try to understand its sources, pathways, and destinations in the wider environment and marine life. In this way, we explore how it impacts the lives of coastal communities and business activities such as aquaculture, fisheries, and tourism. But we also look at how such industries themselves generate plastic waste and how it may enter the marine system.

We examine plastic waste of different sizes ranging from the very large pieces (plastics larger than 50mm) to those that are difficult to see with the human eye (micro-plastics smaller than 5mm). We conduct experiments with the very small micro-plastics to see how they interact with the environment and various organisms, and how they relate to health and disease threats to both animals and humans. On a larger scale, our group of scientists collects data about seasonal changes, tides, waves, and floods to understand and predict the transportation of plastics into the coastal areas. We also collect large plastic waste from the coasts to understand where it comes from and quantify it. In this way, we try to understand how plastic impacts local businesses and communities and engage them in finding solutions to the plastic problem.

By working with Vietnamese businesses, communities, and governmental and non-governmental organisations as well as with business partners in the UK and international NGOs, we will use a broad set of techniques to increase awareness about plastic pollution and find effective solutions to reduce its occurrence and impacts on society.

WHY HAVE I BEEN INVITED TO PARTICIPATE?

You have been invited to participate because you are either a resident, worker, business owner or a government official in the area where we conduct research. As we try to understand the impacts of plastics on local environments, communities, and businesses your insights are very important, and we would like to learn from your experience and observations. We would also like to work with you to find solutions to the plastic pollution problem in Vietnam.

DO I HAVE TO TAKE PART?

It is up to you to decide whether you wish to take part in the research. If you decide to participate, you will be asked to sign an Informed Consent Form (if you are under 18 years old you will be asked to sign an Informed Assent Form, which also requires a signature from your parent or legal guardian). We will also provide an oral consent option where a researcher will read to you the form and record your consent using an audio recorder. If you decide to participate, you are still free to withdraw from the study at a later date without giving a reason why you no longer wish to take part in the project.

WHAT DOES TAKING PART INVOLVE?
Depending on your age, residence, and the work you do, you will be invited to take part in one or several of the research activities carried out by our team of researchers.

Survey/questionnaire: You will be asked a series of questions about the use and disposal of plastic in your household/community/business/province. This survey does not intend to assess your knowledge but to gain insights into how you experience and view the problem of plastic pollution. You are encouraged to answer all questions but if you feel uncomfortable answering some you can leave them blank. The survey will be available online or on paper, and you may be asked to fill it out yourself or with the help of a research assistant. You will be asked to answer questions about your age, gender, residence, and occupation. These will help us establish a demographic understanding of how your experience is similar or varies within a given group (for example, men fishermen or those aged between 18-25).

Interview: You will be asked a series of questions about the everyday use of plastic materials, products, and tools. Depending on your occupation, the interviewer will ask you about the ways in which plastic plays part in your life and how plastic waste impacts what you do. The questions will be open-ended and try to get insight into your observations and experiences. You will be asked to give examples or recall what you have seen or heard about plastic waste in the region. Interviews will be audio or video recorded.

Key informant consultation: You will be asked questions about plastic waste sources and management in an unstructured interview where your knowledge and experiences will lead the conversation. Your position within the community/business sector/government offers a unique perspective on the plastic waste problem and solution endeavours. Interviews will be audio or video recorded.

Focus group discussions: You will be asked to participate in discussing several questions within a small group (12-15 people). The discussion will be led by a researcher who will ask you about your views and observations of plastic pollution in the rivers and coasts of your region. We would like you to share your experiences about how plastic waste affects your household/community/business/province, and what you think are the sources of this plastic, how they move in the marine system, and why people discard them in such ways. You will also be asked to work together in the group to suggest what could be done to deal with the plastic waste problem. Focus group discussions will be audio and video recorded.

'Fishing for plastic' observations: If you are part of the fishing industry, we would like to learn about your work and how plastic waste affects you. You will be asked to record the quantity and location of any plastic waste or any abandoned, lost, or discarded fishing gear that your trawler has caught and how much time it took to clear your nets. A researcher will also ask you questions about the frequency and locations you observe plastic waste during a particular time of the day/month/season/year or areas of the coast. You will also be asked questions about the fishing gear that you use and the ones that get caught in your nets. 'Fishing for plastic' observations and conversations carried with you may be audio or video recorded.

Workshop participation: You will be introduced to a series of issues created by the plastic waste flowing into the rivers and coasts of Vietnam. The workshop facilitators will ask you to participate in several activities ranging from group discussion to playing games designed by the researchers that will help to communicate the effects of plastic on the environment and your community/business/province. You will be asked to work in groups and discuss possible solutions to the plastic problem. These workshops will provide a platform for you and your community to express how plastic waste affects you and to be heard by decision- and policy-makers. Workshops may be video recorded.

ARE THERE ANY POSSIBLE RISKS OR DISADVANTAGES TO TAKING PART?

Participating in this research will not put you at any new risks or disadvantages greater than those you may be normally exposed to in your daily life.

WHAT ARE THE POSSIBLE BENEFITS OF TAKING PART?

Your contribution to this research and the co-development of solutions to the plastic waste problem will directly feed back into your community and help tackle many of the economic and health and safety issues caused by plastic pollution.

WILL I BE REIMBURSED FOR ANY EXPENSES OR FOR MY TIME?

Depending on the type of research activity you are asked to participate in, you may be offered compensation for your time and assistance in the form of gifts or money.

WHAT IF I WANT TO WITHDRAW FROM THE RESEARCH?

Agreeing to participate in this project does not oblige you to remain in the study or to have any further obligations to the research project or team. If at any stage you no longer want to be part of the research, you can withdraw by contacting the Principal Investigator Prof. Michel Kaiser or Co-Investigator Dr. Ngo Thi Thuy Huong.

Because your contributions and the data collected from the surveys and workshops will be used in other formal research outputs (e.g. journal articles, books, reports, policy briefs, conference papers, websites, exhibitions) you are advised to contact the research team at the earliest opportunity should you wish to withdraw from the research. *You can withdraw from other research outputs within* ***two (2) months*** *after your participation in the research activity.*

If you withdraw from the research all of your identifiable data, answers, audio and/or video recordings will be destroyed. We will continue to use the anonymised data collected up to your withdrawal, but we will remove any specific sections (answers, stories, etc.) that you do not want to appear. We will also remove your name from the project files and will not contact you again.

HOW WILL MY DATA BE LOOKED AFTER DURING THE RESEARCH?

All your data will be processed and stored in accordance with the General Data Protection Regulation (GDPR) along with the Data Protection Act 2018 (DPA). The project will also be guided by and adhere to Heriot-Watt University's data protection guidelines and regulations.

Digital research data such as recordings, interview notes, or surveys will be uploaded as soon as possible to secure password-protected folders on Dropbox and SharePoint, and on three encrypted and password-protected devices held by researchers at Heriot-Watt and Phenikaa universities. They will remain there for the duration of the project and will only be accessible to the research team. Your Informed Consent Form (or Informed Assent Form) or oral consent recording will be stored separately from your responses for three years after the research activity. Physical data such as notes and paper questionnaires will be kept in a locked safe or on-person whilst in the field and in transit. Once the data is recorded digitally, the physical copies will be destroyed.

Your contact details (names, addresses, phone numbers, email addresses) will be kept separately from your answers and strictly confidential within the research team. If you agree to be contacted by the research team for further research activities on the same project, we will store this information in password-protected folders on Dropbox and SharePoint until the end of the research project (31 December 2024). After this period, all of this information will be destroyed.

[in case Covid-19 tracing is instantiated again during the research period: The contact details of all participants who take part in an in-person research activity will be stored for 14 days. This information will be kept separately from your answers and strictly confidential within the research team. They will be stored in secure password-protected folders on Dropbox and SharePoint. After 14 days they will be destroyed. If a research facilitator or participant exhibits COVID-19 symptoms within 14 days, you will be contacted. In accordance with public safety and COVID-19 testing and tracing regulations in your province, we may give your contact details to local health authorities.]

WHERE WILL YOU KEEP MY DATA AFTER THE END OF THE RESEARCH?

After the end of the research, all anonymised data will be deposited into two repositories in the UK (UK Data Centre and British Oceanographic Data Centre). Copies will be kept by Prof. Michel Kaiser and Dr. Ngo Thi Thuy Huong on encrypted and password-protected devices and folders in Dropbox.

WHAT WILL YOU DO WITH THE DATA YOU GET FROM ME?

Data collected as part of this research will be used in other research output such as journal articles, books, reports, policy briefs, conference papers, websites, and exhibitions.

WHO IS ORGANISING AND FUNDING THE RESEARCH?

The research is funded by the UKRI Global Challenges Research Fund.

It is conducted by a multidisciplinary team from 8 research institutions and 7 partners in Vietnam covering all regions of the coastline, and Heriot-Watt University in the UK. The team is led by Prof. Michel Kaiser who is a staff member in the Lyell Centre at Heriot-Watt University and by Dr. Ngo Thi Thuy Huong who is a staff member in the Faculty of Biotechnology, Chemistry & Environment at Phenikaa University. The project has been co-developed with Co-Investigators at Heriot-Watt University (Profs Thomas Wagner and Qingping Zou, and Drs Ryan Pereira, Heidi Burdett, Tony Gutierrez, Ingrid Kelling), Phenikaa University (Dr. Nguyen Thi Hanh Tien), Vietnam Academy of Science & Technology (Dr. Mai Huong), ISPONRE (Dr Nguyen Linh), Vietnam National University Hanoi (Dr. Vu Kim Chi), Vietnam National University Ho Chi Minh (Dr. Trinh Bao Son), Vietnam Inst. of Fisheries Econ. & Planning (Mr. Trinh Quang Tu), Fisheries and Technical Economic College (Dr Truong Van Thuong). The research team also includes 4 postdoctoral research associates based at Heriot-Watt University (Drs Priscilla Carrillo-Barragán, Zhiling Liao, Duc Nguyen, and Inna Yaneva-Toraman) and research assistants.

Our partners include UK retailers (The Cooperative, UK; Sainsbury's), major seafood importers and processors (Labeyrie Fine Foods - Lyons Seafood) and a Vietnamese business consortium (IDH-Vietnam) and Vietnamese NGOs Centre for Marine Life, Conservation & Community Development (MCD), and global NGOs the Global Ghost Gear Initiative (GGGI) and Global Aquaculture Alliance (GAA).

WHO HAS APPROVED THIS PROJECT?

This research project has been approved through the research ethics review process of Heriot-Watt University.

CONTACT FOR FURTHER INFORMATION

If you have any further questions about this project, please contact the research team through 3sip2c.project@gmail.com and/or Facebook page – https://www.facebook.com/3sip2c

If you have any concerns about the way in which the project has been conducted, or you wish to make a complaint, you can contact Heriot-Watt University Ethics Committee – Phil Rowsby phil.rowsby@hw.ac.uk or (+44) 131 451 4365

For general information about how Heriot-Watt University looks after research data go to:

https://www.hw.ac.uk/uk/services/docs/information-governance/PrivacyNoticeResearch-V4Finalversion.pdf

If you want more information about this you can also contact the Heriot-Watt University Data Protection Officer at dataprotection@hw.ac.uk

THANK YOU!

Phiếu đồng ý tham gia dự án

Tên dự án: *Nguồn phát thải, nơi tích tụ và các giải pháp nhằm giảm thiểu tác động của rác thải nhựa đến cộng đồng ven biển ở Việt Nam* (3SIP2C)

Trước khi bạn đồng ý tham gia, hãy đảm bảo rằng bạn đã đọc và hiểu Bảng thông tin nghiên cứu và Phiếu đồng ý tham gia dự án này. Sau đó, hãy đảm bảo bạn đánh dấu 'X' vào mỗi ô để thể hiện sự đồng ý của bạn và ký tên vào bên dưới.

Nếu bạn có bất kỳ câu hỏi hoặc thắc mắc nào về biểu mẫu này, bạn nên nói chuyện với người mà bạn cảm thấy thoải mái và vui lòng liên hệ với nhóm nghiên cứu qua Email: 3sip2c.project@gmail.com. Bạn cũng có thể theo dõi trang Facebook của dự án tại https://www.facebook.com/3sip2c và website www.3sip2c.com

Nếu bạn vui lòng tham gia vào nghiên cứu, vui lòng đánh dấu vào từng ô phù hợp và sau đó ký vào biểu mẫu này ở cuối. Để trống bất kỳ ô nào bạn không muốn đồng ý:

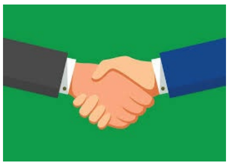

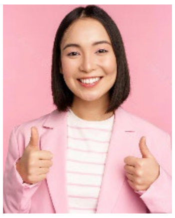

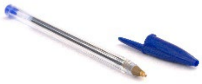

1. Nhóm nghiên cứu đã cho tôi xem / đưa cho tôi một bản sao của Bảng thông tin cho người tham gia và tôi đã có cơ hội đọc và xem xét thông tin.		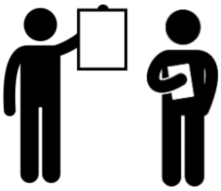	☐
2. Tôi đã có cơ hội để hỏi thêm bất kỳ câu hỏi nào và đã được trả lời những câu hỏi này khiến tôi hài lòng.		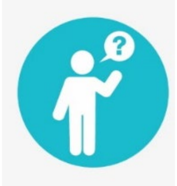	☐
3. Tôi hiểu rằng việc tham gia của tôi là tự nguyện.		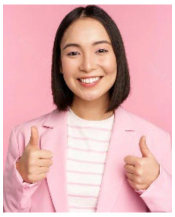	☐
4. Tôi hiểu rằng việc tham gia vào hoạt động nghiên cứu bao gồm:	A) Trả lời câu hỏi trong bảng câu hỏi hoặc phỏng vấn	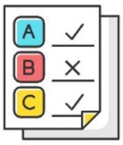 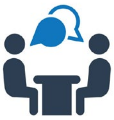	
	B) Tham gia vào một cuộc thảo luận nhóm, nơi tôi sẽ nói chuyện với những người dân ven biển Việt Nam khác, các nhà nghiên cứu và nhân viên của các tổ chức chính phủ và phi chính phủ	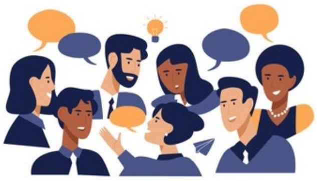	☐
	C) Tham gia đếm rác thải nhựa mắc vào lưới đánh cá và ghi lại tọa độ của nó trên giấy hoặc trên ứng dụng điện thoại	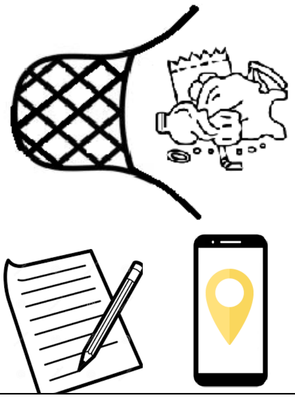	
5. Tôi hiểu rằng hoạt động nghiên cứu sẽ được ghi lại âm thanh / hình ảnh/ quay phim.		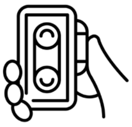 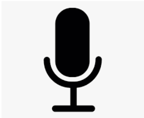 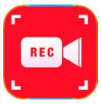	☐

<table>
<tr><td>6. Tôi hiểu rằng tất cả thông tin cá nhân thu thập được về tôi, chẳng hạn như tên và địa chỉ của tôi sẽ được nhóm nghiên cứu giữ bí mật tuyệt đối và sẽ không được đưa vào bất kỳ báo cáo công khai nào.</td><td>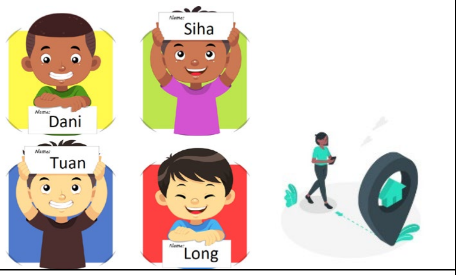
</td><td>☐</td></tr>
<tr><td>7. Tôi đồng ý được nhóm nghiên cứu liên hệ cho các hoạt động nghiên cứu trong tương lai về cùng một dự án.</td><td>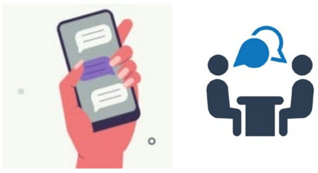</td><td>☐</td></tr>
<tr><td>8. Tôi hiểu rằng tên của tôi và bất kỳ thông tin nào có thể nhận dạng tôi sẽ bị xóa để giữ cho danh tính của tôi ẩn danh</td><td>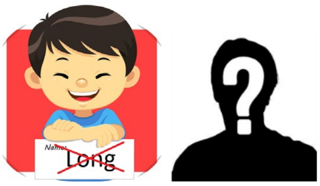
</td><td>☐</td></tr>
<tr><td>9. Tôi đã được thông báo và hiểu cách dữ liệu và bản ghi của tôi sẽ được lưu trữ và sử dụng trong và sau khi kết thúc nghiên cứu.</td><td>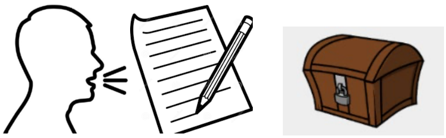</td><td>☐</td></tr>
<tr><td>10. Tôi đồng ý để các bản ghi âm, ghi hình của tôi được sao chép lại với mục đích phi thương mại trong các báo cáo, bài thuyết trình, ấn phẩm, trang web và triển lãm liên quan đến dự án nghiên cứu.

Tôi đồng ý để khuôn mặt của mình xuất hiện đầy đủ trong đoạn phim và / hoặc bức ảnh.</td><td>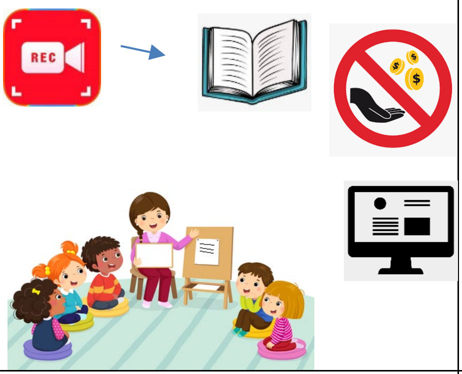
</td><td>☐</td></tr>
<tr><td>11. Tôi hiểu rằng lời nói của tôi có thể được trích dẫn trong các ấn phẩm học thuật, bài báo, sách, báo cáo, trang web của dự án và các kết quả đầu ra liên quan khác của dự án sẽ chỉ được sử dụng cho các mục đích phi thương mại.</td><td>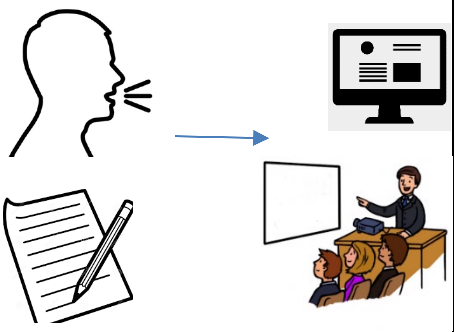</td><td>☐</td></tr>
<tr><td>12. Tôi đồng ý để dữ liệu ẩn danh mà tôi cung cấp sẽ được lưu giữ trong bộ nhớ an toàn để sử dụng trong tương lai cho các dự án tương tự và có liên quan.</td><td>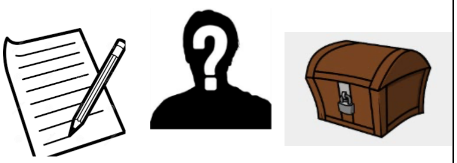</td><td>☐</td></tr>
</table>

13. Tôi hiểu rằng Mẫu đồng ý được cung cấp thông tin này sẽ được nhóm nghiên cứu lưu giữ trong thời gian 3 năm.		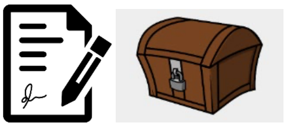	
14. Tôi hiểu rằng tôi có thể yêu cầu:	A) Rút khỏi dự án nghiên cứu	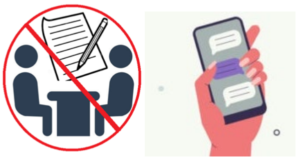	
	B) Rút lại sự chấp thuận của tôi để sử dụng các bản ghi âm, ghi hình	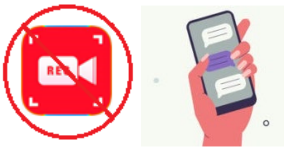	
	C) Xóa các trích dẫn cụ thể khỏi kết quả nghiên cứu	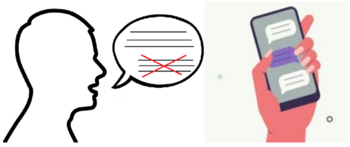	
15. Bằng cách liên hệ với nhóm nghiên cứu nhóm nghiên cứu qua 3sip2c.project@gmail.com		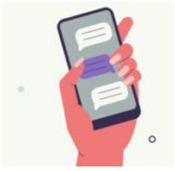	

Tôi tên là ____ đồng ý tham gia dự án nghiên cứu này.

Chữ ký : ____ Ngày: __________________________

Tên người lấy phiếu đồng ý tham gia ____

Chữ ký : ____ Ngày: __________________________

THÔNG TIN VỀ DỰ ÁN CHO NGƯỜI THAM GIA

Tên dự án
Nguồn phát thải, nơi tích tụ và các giải pháp nhằm giảm thiểu tác động của rác thải nhựa đến cộng đồng ven biển ở Việt Nam (3SIP2C)

Thư mời

Bạn được mời tham gia vào một dự án nghiên cứu. Trước khi bạn quyết định có tham gia hay không, điều quan trọng là phải hiểu tại sao nghiên cứu này được tiến hành và nó sẽ liên quan đến những gì. Xin vui lòng dành thời gian để đọc các thông tin sau một cách cẩn thận. Đặt câu hỏi nếu có bất cứ điều gì không rõ ràng hoặc nếu bạn muốn biết thêm thông tin.

Mục đích của dự án là gì?
Dự án của chúng tôi nghiên cứu về rác thải nhựa đi vào các con sông và bờ biển của Việt Nam. Chúng tôi cố gắng hiểu nguồn gốc, con đường đi và đích đến của nó trong môi trường và sinh vật biển. Bằng cách này, chúng tôi khám phá cách rác nhựa tác động đến cuộc sống của các cộng đồng ven biển và các hoạt động kinh doanh như nuôi trồng đánh bắt thủy hải sản và du lịch. Nhưng chúng tôi cũng xem xét bản thân các ngành công nghiệp đó tạo ra rác nhựa như thế nào và liệu chúng có thể đi vào môi trường biển như thế nào.
Chúng tôi nghiên cứu nhựa với các kích cỡ khác nhau, từ những mảnh rất lớn (nhựa lớn hơn 50 mm) đến những loại khó nhìn thấy bằng mắt thường (vi nhựa nhỏ hơn 5 mm). Chúng tôi tiến hành các thí nghiệm với vi nhựa rất nhỏ để xem cách chúng tương tác với môi trường và các sinh vật khác nhau, cũng như cách chúng liên quan đến các mối đe dọa về sức khỏe và bệnh tật đối với cả động vật và con người. Ở quy mô lớn hơn, nhóm các nhà khoa học của chúng tôi thu thập dữ liệu về sự thay đổi theo mùa, thủy triều, sóng và lũ lụt để hiểu và dự đoán quá trình vận chuyển rác nhựa vào các khu vực ven biển. Chúng tôi cũng thu gom rác thải nhựa lớn từ các bờ biển để tìm hiểu xem chúng đến từ đâu và định lượng chúng. Bằng cách này, chúng tôi cố gắng hiểu tác động của nhựa đến các doanh nghiệp và cộng đồng địa phương và thu hút họ tìm giải pháp cho vấn đề nhựa.
Bằng cách hợp tác với các doanh nghiệp, cộng đồng và các tổ chức chính phủ và phi chính phủ Việt Nam cũng như với các đối tác kinh doanh ở Vương quốc Anh và các tổ chức phi chính phủ quốc tế, chúng tôi sẽ sử dụng một loạt các kỹ thuật để nâng cao nhận thức về ô nhiễm nhựa và tìm ra các giải pháp hiệu quả để giảm thiểu sự xuất hiện và sự tác động của chúng đến xã hội.

Tại sao tôi được mời tham gia?

Bạn được mời tham gia vì bạn là cư dân, công nhân, chủ doanh nghiệp hoặc cán bộ trong khu vực chúng tôi tiến hành nghiên cứu. Khi chúng tôi cố gắng tìm hiểu tác động của nhựa đối với môi trường, cộng đồng và doanh nghiệp địa phương, hiểu biết của bạn rất quan trọng và chúng tôi muốn học hỏi từ kinh nghiệm và quan sát của bạn. Chúng tôi cũng muốn hợp tác với bạn để tìm giải pháp cho vấn đề ô nhiễm nhựa tại Việt Nam.

Tôi có phải tham gia không?

Bạn có quyền quyết định xem bạn có muốn tham gia vào nghiên cứu hay không. Nếu bạn quyết định tham gia, bạn sẽ được yêu cầu ký vào Mẫu chấp thuận tham gia dự án (nếu bạn dưới 18 tuổi, bạn sẽ được yêu cầu ký vào Mẫu chấp thuận bảo lãnh, mẫu này cũng yêu cầu chữ ký của cha mẹ hoặc người giám hộ hợp pháp của bạn). Chúng tôi cũng sẽ cung cấp tùy chọn đồng ý bằng miệng trong đó nhà nghiên cứu sẽ đọc biểu mẫu cho bạn nghe và ghi lại sự đồng ý của bạn bằng máy ghi âm. Nếu bạn quyết định tham gia, bạn vẫn có quyền rút khỏi nghiên cứu vào một ngày sau đó mà không cần đưa ra lý do tại sao bạn không còn muốn tham gia dự án nữa.

Tham gia bao gồm những gì?
Tùy thuộc vào độ tuổi, nơi cư trú và công việc bạn làm, bạn sẽ được mời tham gia vào một hoặc một số hoạt động nghiên cứu do nhóm các nhà nghiên cứu của chúng tôi thực hiện.

Khảo sát/bảng câu hỏi: Bạn sẽ được hỏi một loạt câu hỏi về việc sử dụng và thải bỏ nhựa trong hộ gia đình/cộng đồng/doanh nghiệp/tỉnh của bạn. Cuộc khảo sát này không nhằm mục đích đánh giá kiến thức của bạn mà để hiểu rõ hơn về cách bạn trải nghiệm và nhìn nhận vấn đề ô nhiễm nhựa. Bạn được khuyến khích trả lời tất cả các câu hỏi nhưng nếu bạn cảm thấy không thoải mái khi trả lời một số câu hỏi, bạn có thể để trống. Bản khảo sát sẽ có sẵn trực tuyến hoặc trên giấy và bạn có thể được yêu cầu tự điền vào hoặc với sự trợ giúp của trợ lý nghiên cứu. Bạn sẽ được yêu cầu trả lời các câu hỏi về tuổi tác, giới tính, nơi cư trú và nghề nghiệp của bạn. Những điều này sẽ giúp chúng tôi thiết lập sự hiểu biết về nhân khẩu học về trải nghiệm của bạn tương tự hoặc khác nhau như thế nào trong một nhóm nhất định (ví dụ: nam ngư dân hoặc những người trong độ tuổi từ 18-25).

Phỏng vấn: Bạn sẽ được hỏi một loạt câu hỏi về việc sử dụng hàng ngày các vật liệu, sản phẩm và công cụ bằng nhựa. Tùy thuộc vào nghề nghiệp của bạn, người phỏng vấn sẽ hỏi bạn về vai trò của nhựa trong cuộc sống của bạn và rác thải nhựa ảnh hưởng đến công việc của bạn như thế nào. Các câu hỏi sẽ có kết thúc mở và cố gắng hiểu sâu hơn về những quan sát và kinh nghiệm của bạn. Bạn sẽ được yêu cầu đưa ra các ví dụ hoặc nhớ lại những gì bạn đã thấy hoặc nghe về rác thải nhựa trong khu vực. Các cuộc phỏng vấn sẽ được ghi lại bằng âm thanh hoặc video.

Tham vấn người cung cấp thông tin chính: Bạn sẽ được hỏi các câu hỏi về nguồn và quản lý chất thải nhựa trong một cuộc phỏng vấn không có cấu trúc sẵn, trong đó kiến thức và kinh nghiệm của bạn sẽ dẫn dắt cuộc trò chuyện. Vị trí của bạn trong cộng đồng/lĩnh vực kinh doanh/phòng ban chức năng mang đến góc nhìn độc đáo về vấn đề rác thải nhựa và các nỗ lực giải quyết. Các cuộc phỏng vấn sẽ được ghi lại bằng âm thanh hoặc video.

Thảo luận nhóm tập trung: Bạn sẽ được yêu cầu tham gia thảo luận một số câu hỏi trong một nhóm nhỏ (12-15 người). Cuộc thảo luận sẽ được dẫn dắt bởi một nhà nghiên cứu, người sẽ hỏi bạn về quan điểm và quan sát của bạn về ô nhiễm nhựa ở các con sông và bờ biển trong khu vực của bạn. Chúng tôi muốn bạn chia sẻ kinh nghiệm của mình về việc rác thải nhựa ảnh hưởng đến hộ gia đình/cộng đồng/doanh nghiệp/tỉnh của bạn như thế nào và bạn nghĩ nguồn gốc của loại nhựa này là gì, cách chúng di chuyển trong hệ thống biển và tại sao mọi người lại vứt bỏ chúng theo những cách như vậy. Bạn cũng sẽ được yêu cầu làm việc cùng nhau trong nhóm để đề xuất những gì có thể được thực hiện để giải quyết vấn đề rác thải nhựa. Các cuộc thảo luận nhóm tập trung sẽ được ghi lại bằng âm thanh và video.

Các quan sát về 'thu gom rác thải nhựa bằng tàu cá': Nếu bạn làm trong ngành đánh bắt cá, chúng tôi muốn tìm hiểu về công việc của bạn và rác thải nhựa ảnh hưởng đến bạn như thế nào. Bạn sẽ được yêu cầu ghi lại số lượng và vị trí của bất kỳ chất thải nhựa nào hoặc bất kỳ ngư cụ bị bỏ rơi, bị mất hoặc bị loại bỏ nào mà tàu đánh cá của bạn đã thu được trong lưới và mất bao nhiêu thời gian để vệ sinh lưới của bạn. Một nghiên cứu viên cũng sẽ đặt câu hỏi cho bạn về tần suất và địa điểm bạn quan sát thấy rác thải nhựa trong một thời điểm cụ thể trong ngày/tháng/mùa/năm hoặc các khu vực trên bờ biển. Bạn cũng sẽ được hỏi những câu hỏi về ngư cụ mà bạn sử dụng và những ngư cụ bị mắc vào lưới của bạn. Các quan sát và cuộc trò chuyện về hoạt động 'thu gom rác thải nhựa bằng tàu cá' có thể được ghi lại bằng âm thanh hoặc video.

Tham gia hội thảo: Bạn sẽ được giới thiệu về một loạt các vấn đề do rác thải nhựa đổ ra sông và bờ biển Việt Nam. Những người điều hành hội thảo sẽ yêu cầu bạn tham gia vào một số hoạt động, từ thảo luận nhóm đến chơi trò chơi do các nhà nghiên cứu thiết kế để giúp truyền đạt về tác động của nhựa đối với môi trường và cộng đồng/doanh nghiệp/tỉnh của bạn. Bạn sẽ được yêu cầu làm việc theo nhóm và thảo luận các giải pháp khả thi cho vấn đề nhựa. Những hội thảo này sẽ cung cấp một nền tảng để bạn và cộng đồng của bạn bày tỏ mức độ ảnh hưởng của rác thải nhựa đến bạn và được các nhà hoạch định chính sách và ra quyết định lắng nghe. Hội thảo có thể được quay video.

có bất kỳ rủi ro hoặc bất lợi nào có thể xảy ra khi tham gia không?

Việc tham gia vào nghiên cứu này sẽ không đặt bạn vào bất kỳ rủi ro hoặc bất lợi mới nào lớn hơn những rủi ro hoặc bất lợi mà bạn có thể gặp phải trong cuộc sống hàng ngày.

Những lợi ích có thể có của việc tham gia là gì?

Đóng góp của bạn cho nghiên cứu này và việc đồng phát triển các giải pháp cho vấn đề rác thải nhựa sẽ trực tiếp phản hồi lại cộng đồng của bạn và giúp giải quyết nhiều vấn đề về kinh tế, sức khỏe và an toàn do ô nhiễm nhựa gây ra.

Tôi sẽ được hoàn trả cho bất kỳ chi phí hoặc thời gian của tôi?

Tùy thuộc vào loại hoạt động nghiên cứu mà bạn được yêu cầu tham gia, bạn có thể được đền bù cho thời gian và sự hỗ trợ của mình dưới dạng quà tặng hoặc tiền bạc.

Nếu tôi muốn rút khỏi nghiên cứu thì sao?

Việc đồng ý tham gia vào dự án này không bắt buộc bạn phải tiếp tục tham gia nghiên cứu hoặc có bất kỳ nghĩa vụ nào khác đối với dự án hoặc nhóm nghiên cứu. Nếu ở bất kỳ giai đoạn nào bạn không còn muốn tham gia nghiên cứu nữa, bạn có thể rút lui bằng cách liên hệ với nhóm nghiên cứu tại 3sip2c.project@gmail.com hoặc bạn cũng có thể truy cập trang Facebook của chúng tôi – https://www.facebook.com/3sip2c hoặc trang web của chúng tôi www.3sip2c.com nơi bạn có thể tìm thấy thông tin về nghiên cứu của chúng tôi.

Vì đóng góp của bạn và dữ liệu thu thập được từ các cuộc khảo sát và hội thảo sẽ được sử dụng trong các kết quả nghiên cứu chính thức khác (ví dụ: bài báo, sách, báo cáo, tóm tắt chính sách, tài liệu hội nghị, trang web, triển lãm), bạn nên liên hệ với nhóm nghiên cứu sớm nhất cơ hội nếu bạn muốn rút khỏi nghiên cứu. *Mọi tài liệu xuất bản sẽ bị gỡ xuống hoặc chỉnh sửa theo yêu cầu của bạn nếu bạn muốn rút lại sự đồng ý của mình.*

Nếu bạn rút khỏi nghiên cứu, tất cả dữ liệu nhận dạng, câu trả lời, bản ghi âm và/hoặc video của bạn sẽ bị hủy. Chúng tôi sẽ tiếp tục sử dụng dữ liệu ẩn danh được thu thập cho đến khi bạn rút khỏi nghiên cứu, nhưng chúng tôi sẽ xóa bất kỳ phần cụ thể nào (câu trả lời, câu chuyện, v.v.) mà bạn không muốn xuất hiện. Chúng tôi cũng sẽ xóa tên của bạn khỏi các văn bản của dự án và sẽ không liên hệ lại với bạn.

Dữ liệu của tôi sẽ được quản lý như thế nào trong quá trình nghiên cứu?

Tất cả dữ liệu của bạn sẽ được xử lý và lưu trữ theo Quy định chung về bảo vệ dữ liệu (GDPR) cùng với Đạo luật bảo vệ dữ liệu 2018 (DPA). Dự án cũng sẽ được hướng dẫn và tuân thủ các nguyên tắc và quy định bảo vệ dữ liệu của Đại học Heriot-Watt.

Dữ liệu nghiên cứu kỹ thuật số như bản ghi, ghi chú phỏng vấn hoặc khảo sát sẽ được tải lên ngay khi có thể vào các thư mục được bảo vệ bằng mật khẩu trên Dropbox và SharePoint, cũng như trên ba thiết bị được mã hóa và bảo vệ bằng mật khẩu do các nhà nghiên cứu tại các trường đại học Heriot-Watt và Phenikaa nắm giữ . Chúng sẽ ở đó trong suốt thời gian của dự án và chỉ nhóm nghiên cứu mới có thể truy cập được. Biểu mẫu đồng ý tham gia dự án (hoặc Biểu mẫu đồng ý có sự bảo lãnh) hoặc bản ghi âm lại sự đồng ý bằng miệng sẽ được lưu trữ tách biệt với các câu trả lời của bạn trong ba năm sau hoạt động nghiên cứu. Dữ liệu vật lý như ghi chú và bảng câu hỏi trên giấy sẽ được lưu giữ trong két an toàn có khóa hoặc ở bên người khi ở hiện trường và trên đường vận chuyển. Sau khi dữ liệu được ghi lại bằng kỹ thuật số, các bản sao vật lý sẽ bị hủy.

Chi tiết liên hệ của bạn (tên, địa chỉ, số điện thoại, địa chỉ email) sẽ được giữ riêng biệt với câu trả lời của bạn và được bảo mật nghiêm ngặt trong nhóm nghiên cứu. Nếu bạn đồng ý để nhóm nghiên cứu liên hệ về các hoạt động nghiên cứu tiếp theo trong cùng một dự án, chúng tôi sẽ lưu trữ thông tin này trong các thư mục được bảo vệ bằng mật khẩu trên Dropbox và SharePoint cho đến khi kết thúc dự án nghiên cứu (ngày 31 tháng 12 năm 2024). Sau khoảng thời gian này, tất cả các thông tin này sẽ bị hủy.
[Trong trường hợp truy tìm người nhiễm Covid-19 được bắt đầu lại trong thời gian nghiên cứu: Chi tiết liên hệ của tất cả những người tham gia hoạt động nghiên cứu trực tiếp sẽ được lưu trữ trong 14 ngày. Thông tin này sẽ được giữ riêng biệt với câu trả lời của bạn và được bảo mật nghiêm ngặt trong nhóm nghiên cứu. Chúng sẽ được lưu trữ trong các thư mục được bảo vệ bằng mật khẩu an toàn trên Dropbox và SharePoint. Sau 14 ngày chúng sẽ bị xóa. Nếu người hướng dẫn nghiên cứu hoặc người tham gia có các triệu chứng COVID-19 trong vòng 14 ngày, chúng tôi sẽ liên hệ với bạn. Theo các quy định về an toàn công cộng cũng như xét nghiệm và truy tìm COVID-19 tại tỉnh của bạn, chúng tôi có thể cung cấp chi tiết liên hệ của bạn cho các cơ quan y tế địa phương.]

Bạn sẽ giữ dữ liệu của tôi ở đâu sau khi kết thúc nghiên cứu?

Sau khi kết thúc nghiên cứu, tất cả dữ liệu được ẩn danh sẽ được gửi vào hai kho lưu trữ tại Vương quốc Anh (Trung tâm Dữ liệu Vương quốc Anh và Trung tâm Dữ liệu Hải dương học Anh) và một kho lưu trữ tại Việt Nam. Các bản sao sẽ được giữ bởi Giáo sư Michel Kaiser và Tiến sĩ. Ngô Thị Thùy Hương trên các thiết bị và thư mục được mã hóa và bảo vệ bằng mật khẩu trong Dropbox.

Bạn sẽ làm gì với dữ liệu bạn nhận được từ tôi?

Dữ liệu được thu thập như một phần của nghiên cứu này sẽ được sử dụng trong các kết quả nghiên cứu khác như bài báo, sách, báo cáo, tóm tắt chính sách, tài liệu hội nghị, trang web và triển lãm.

Ai đang tổ chức và tài trợ cho nghiên cứu?

Nghiên cứu được tài trợ bởi Quỹ Nghiên cứu Thách thức Toàn cầu của UKRI.

Dự án được thực hiện bởi một nhóm đa ngành từ 8 tổ chức nghiên cứu và 7 đối tác tại Việt Nam và Đại học Heriot-Watt ở Vương quốc Anh. Nhóm do Giáo sư Michel Kaiser, cán bộ Trung tâm Lyell, Đại học Heriot-Watt làm trưởng nhóm và TS Ngô Thị Thùy Hương, cán bộ Khoa Công nghệ Sinh học, Hóa học & Môi trường , Đại học Phenikaa . Dự án được đồng phát triển với các Đồng điều tra viên tại Đại học Heriot-Watt (Giáo sư Thomas Wagner và Qingping Zou, và Tiến sĩ Ryan Pereira, Heidi Burdett, Tony Gutierrez, Ingrid Kelling), Đại học Phenikaa (Tiến sĩ Nguyễn Thị Hạnh Tiên), Đại học Khoa học và Công nghệ Hà Nội (USTH) (TS Mai Hương), ISPONRE (TS Nguyễn Sỹ Linh), Đại học Quốc gia Hà Nội (TS Vũ Kim Chi), Viện Kinh tế & Quy hoạch Thủy sản (Trịnh Quang Tú), và Trường Cao đẳng Kinh tế Kỹ thuật Thủy sản (TS Trương Văn Thượng). Nhóm nghiên cứu cũng bao gồm các cộng sự nghiên cứu sau tiến sĩ tại Đại học Heriot-Watt (Tiến sĩ Priscilla Carrillo- Barragán , Zhiling Liao, Duc Nguyen và Inna Yaneva-Toraman), ĐHQGHN (TS Giang Văn Trọng , Phạm Văn Mạnh , Đặng Kinh Bắc), Đại học Phenikaa (TS Lê Thanh Thảo), USTH (TS Lê Phương Thu); trợ lý nghiên cứu (Đỗ Thị Nhung, Phan Phương Thanh, Nguyễn Thị Hoài Thương , Trần Thị Hòa , Nguyễn Danh Thiện , Vũ Hoàng Thùy Dương, Lê Nam, Chu Trung Tiến, Vũ Thị Thu Hiền, Đặng Hoàng Hà, Vũ Khánh Chi) và các kỹ thuật viên phòng thí nghiệm (Dr Juliane Bischoff và Ngô Thị Thảo Nhi) .
Các đối tác ngoài học thuật của chúng tôi bao gồm các nhà bán lẻ tại Vương quốc Anh (The Cooperative, UK; Sainsbury's), các nhà nhập khẩu và chế biến thủy sản lớn (Labeyrie Fine Foods - Lyons Seafood) và một tập đoàn kinh doanh Việt Nam (IDH-Vietnam) và các tổ chức phi chính phủ Việt Nam Trung tâm Sinh vật biển, Bảo tồn & Phát triển Cộng đồng (MCD) và các tổ chức phi chính phủ toàn cầu Sáng kiến Lưới ma Toàn cầu (GGGI) và Liên minh Nuôi trồng Thủy sản Toàn cầu (GAA).

Ai đã phê duyệt dự án này?
Dự án nghiên cứu này đã được phê duyệt thông qua quy trình xem xét đạo đức nghiên cứu của Đại học Heriot-Watt.

Liên hệ để biết thêm thông tin
Nếu bạn có thêm bất kỳ câu hỏi nào về dự án này, vui lòng liên hệ với nhóm nghiên cứu qua 3sip2c.project@gmail.com

Bạn cũng có thể truy cập trang Facebook của chúng tôi – https://www.facebook.com/Sources-Sinks-and-Solutions-for-Impacts-of-Plastics-on-Coast-of-Vietnam-103983809012907/ hoặc trang web của chúng tôi www.3sip2c.com nơi bạn có thể tìm thấy thông tin về nghiên cứu của chúng tôi.
Nếu bạn có bất kỳ lo ngại nào về cách thức thực hiện dự án hoặc bạn muốn khiếu nại, bạn có thể liên hệ với Ủy ban Đạo đức của Đại học Heriot-Watt – Phil Rowsby phil.rowsby@hw.ac.uk hoặc (+44) 131 451 4365

Để biết thông tin chung về cách Đại học Heriot-Watt xử lý dữ liệu nghiên cứu, hãy truy cập:

https://www.hw.ac.uk/uk/services/docs/information-governance/PrivacyNoticeResearch-V4Finalversion.pdf

Nếu bạn muốn biết thêm thông tin về điều này, bạn cũng có thể liên hệ với Nhân viên bảo vệ dữ liệu của Đại học Heriot-Watt tại dataprotection@hw.ac.uk

Cảm ơn bạn!
