# Supplementary material for: Marine plastic pollution undermines the livelihoods and income of fishing communities in Viet Nam
Source: Commun Earth Environ. 2026 Jun 8;7(1):452. doi: 10.1038/s43247-026-03567-z (PMC13246506; doi:10.1038/s43247-026-03567-z)
Supplement: Supplementary file 3 — Reporting Summary [file 43247_2026_3567_MOESM3_ESM.pdf]

nature portfolio

Corresponding author(s): Michel Kaiser

Last updated by author(s): 30 Mar 2026

Reporting Summary

Nature Portfolio wishes to improve the reproducibility of the work that we publish. This form provides structure for consistency and transparency in reporting. For further information on Nature Portfolio policies, see our Editorial Policies and the Editorial Policy Checklist.

Statistics

For all statistical analyses, confirm that the following items are present in the figure legend, table legend, main text, or Methods section.

n/a	Confirmed	
☐	☒	The exact sample size (*n*) for each experimental group/condition, given as a discrete number and unit of measurement
☐	☒	A statement on whether measurements were taken from distinct samples or whether the same sample was measured repeatedly
☐	☒	The statistical test(s) used AND whether they are one- or two-sided<br>*Only common tests should be described solely by name; describe more complex techniques in the Methods section.*
☐	☒	A description of all covariates tested
☐	☒	A description of any assumptions or corrections, such as tests of normality and adjustment for multiple comparisons
☐	☒	A full description of the statistical parameters including central tendency (e.g. means) or other basic estimates (e.g. regression coefficient) AND variation (e.g. standard deviation) or associated estimates of uncertainty (e.g. confidence intervals)
☐	☒	For null hypothesis testing, the test statistic (e.g. *F*, *t*, *r*) with confidence intervals, effect sizes, degrees of freedom and *P* value noted<br>*Give P values as exact values whenever suitable.*
☐	☒	For Bayesian analysis, information on the choice of priors and Markov chain Monte Carlo settings
☐	☒	For hierarchical and complex designs, identification of the appropriate level for tests and full reporting of outcomes
☐	☒	Estimates of effect sizes (e.g. Cohen's *d*, Pearson's *r*), indicating how they were calculated

Our web collection on statistics for biologists contains articles on many of the points above.

Software and code

Policy information about availability of computer code

Data collection	No software was used for data collection
Data analysis	Data analysis was conducted using IBM SPSS Statistics software (Version 29.0.1.0 (171))

For manuscripts utilizing custom algorithms or software that are central to the research but not yet described in published literature, software must be made available to editors and reviewers. We strongly encourage code deposition in a community repository (e.g. GitHub). See the Nature Portfolio guidelines for submitting code & software for further information.

Data

Policy information about availability of data

All manuscripts must include a data availability statement. This statement should provide the following information, where applicable:

- Accession codes, unique identifiers, or web links for publicly available datasets
- A description of any restrictions on data availability
- For clinical datasets or third party data, please ensure that the statement adheres to our policy

The data for graphs in this article are publicly available through Figshare at: https://doi.org/10.6084/m9.figshare.31882999. The raw datasets used in this study are included in the article, supplementary information and are publicly available through PURE at https://doi.org/10.17861/47e741f5-3810-4f40-9b03-fba8cf825913 . Any additional information is available upon request to the corresponding author.

Research involving human participants, their data, or biological material

Policy information about studies with human participants or human data. See also policy information about sex, gender (identity/presentation), and sexual orientation and race, ethnicity and racism.

Reporting on sex and gender	Gender of participants was self-reported and reported in the demographics of interviewees. Analyses did not stratify by sex or gender, as this was not central to the research questions.
Reporting on race, ethnicity, or other socially relevant groupings	Information on ethnicity, or other socially relevant groupings was self-reported where relevant and used only to describe the study population. These variables were not used as proxies for socioeconomic status or other factors.
Population characteristics	The study population comprised of active nearshore fishers in study sites in Vietnam.
Recruitment	Participants were recruited through a combination of snowball sampling, initiated with key informants (e.g., governmental officers from the Department of Fisheries or leaders of fishing groups within the commune), and followed by convenient sampling with fishers at different ports.
Ethics oversight	The research was subject to full ethics approval through the Heriot-Watt University ethics committee: approval number 2023-3366-7400. All participants provided informed consent, and data were anonymised prior to analysis.

Note that full information on the approval of the study protocol must also be provided in the manuscript.

Field-specific reporting

Please select the one below that is the best fit for your research. If you are not sure, read the appropriate sections before making your selection.

☐ Life sciences ☒ Behavioural & social sciences ☐ Ecological, evolutionary & environmental sciences

For a reference copy of the document with all sections, see nature.com/documents/nr-reporting-summary-flat.pdf

Behavioural & social sciences study design

All studies must disclose on these points even when the disclosure is negative.

Study description	Mixed-method study with nearshore fishers
Research sample	We used a mixed methods design to quantify impacts of marine plastics based on structured interviews with nearshore fishers (n = 199) and subsequently verified these findings through additional fisher interviews (n = 94) and waste audits of marine plastics caught in fishing nets (n = 282) in the same study areas.
Sampling strategy	Participants were recruited through a combination of snowball sampling, initiated with key informants (e.g., governmental officers from the Department of Fisheries or leaders of fishing groups within the commune), and followed by convenient sampling with fishers at different ports.
Data collection	Data were collected by researchers in pen and paper through in-person structured interview forms with fishers. Data from waste audit of marine debris were collected by sea observer following standard method.
Timing	Structured Interview data with 199 fishers was collected from March to July 2023; and the validation interview with 94 fishers was conducted in May 2024.<br>Waste audit data were collected during two seasons (Dry season: 17th April to 30th May, 2024; Wet season: 1st August to 30th September, 2024)
Data exclusions	No data was excluded from the analyses.
Non-participation	No participant was dropped out.
Randomization	Participants were not allocated into experimental groups

Reporting for specific materials, systems and methods

We require information from authors about some types of materials, experimental systems and methods used in many studies. Here, indicate whether each material, system or method listed is relevant to your study. If you are not sure if a list item applies to your research, read the appropriate section before selecting a response.

Materials & experimental systems

n/a	Involved in the study
☒	☐ Antibodies
☒	☐ Eukaryotic cell lines
☒	☐ Palaeontology and archaeology
☒	☐ Animals and other organisms
☒	☐ Clinical data
☒	☐ Dual use research of concern
☒	☐ Plants

Methods

n/a	Involved in the study
☒	☐ ChIP-seq
☒	☐ Flow cytometry
☒	☐ MRI-based neuroimaging

Plants

Seed stocks

Report on the source of all seed stocks or other plant material used. If applicable, state the seed stock centre and catalogue number. If plant specimens were collected from the field, describe the collection location, date and sampling procedures.

Novel plant genotypes

Describe the methods by which all novel plant genotypes were produced. This includes those generated by transgenic approaches, gene editing, chemical/radiation-based mutagenesis and hybridization. For transgenic lines, describe the transformation method, the number of independent lines analyzed and the generation upon which experiments were performed. For gene-edited lines, describe the editor used, the endogenous sequence targeted for editing, the targeting guide RNA sequence (if applicable) and how the editor was applied.

Authentication

Describe any authentication procedures for each seed stock used or novel genotype generated. Describe any experiments used to assess the effect of a mutation and, where applicable, how potential secondary effects (e.g. second site T-DNA insertions, mosiacism, off-target gene editing) were examined.
