# Supplementary material for: Marine plastic pollution undermines the livelihoods and income of fishing communities in Viet Nam
Source: Commun Earth Environ. 2026 Jun 8;7(1):452. doi: 10.1038/s43247-026-03567-z (PMC13246506; doi:10.1038/s43247-026-03567-z)
Supplement: Supplementary file 1 — Transparent Peer Review file [file 43247_2026_3567_MOESM1_ESM.pdf]

nature portfolio

Peer Review File

Marine plastic pollution undermines the livelihoods and income of fishing communities in Vietnam

Corresponding Author: Professor Michel Kaiser

This manuscript has been previously reviewed at another journal. This document only contains information relating to versions considered at Communications Earth & Environment.

This file contains all editorial decision letters in order by version, followed by all author rebuttals in order by version.

Attachments originally included by the reviewers as part of their assessment can be found at the end of this file.

Version 0:

Decision Letter:

** Please ensure you delete the link to your author home page in this e-mail if you wish to forward it to your coauthors **

Dear Professor Kaiser,

Your manuscript titled "Marine plastic pollution undermines the livelihoods and income of fishing communities in Vietnam" has now been seen by 3 reviewers, whose comments are appended below. You will see that they find your work of some potential interest. However, they have raised substantial concerns that must be addressed. In light of these comments, extensive revisions will be required before we can further consider the manuscript for publication. We would, however, be interested in considering a revised version that fully addresses these serious concerns.

We hope you will find the reviewers' comments useful as you decide how to proceed. If additional work allows you to either incorporate or refute these criticisms, we will be happy to look at a substantially revised manuscript. If you choose to take up this option, please either highlight all changes in the manuscript text file, or provide a list of the changes to the manuscript with your responses to the reviewers.

To be publishable in Communications Earth and Environment the following editorial thresholds need to be addressed.

*Provide compelling novel insight into the definition of the marine plastic pollution highlighting the cost burden from fishing vessels and income factors.

* Transparently report the research gap and improve the presentation of the main conclusions by properly connecting the text, figures and supplementary information.

When resubmitting, please provide a point-by-point response to the reviewers' comments. Please submit your responses as a separate file, distinct from your cover letter where you can add responses to the Editors' comments that you do not want to be made available to the reviewers. Word files are preferred. We recommend that any figures, tables or graphs that are included in the response to reviewers are also included in the main article or Supplementary Information.

Please bear in mind that we will be reluctant to approach the reviewers again in the absence of substantial revisions.

If the revision process takes significantly longer than three months, we will be happy to reconsider your paper at a later date, as long as nothing similar has been accepted for publication at Communications Earth & Environment or published elsewhere in the meantime.

We are committed to providing a fair and constructive peer-review process. Please do not hesitate to contact us if you wish to discuss the revision in more detail.

Please use the following link to submit your revised manuscript, point-by-point response to the reviewers' comments with a

list of your changes to the manuscript text (which should be in a separate document to any cover letter), a tracked-changes version of the manuscript (as a PDF file) and any completed checklist:

Link Redacted

** This url links to your confidential home page and associated information about manuscripts you may have submitted or be reviewing for us. If you wish to forward this email to co-authors, please delete the link to your homepage first **

Please do not hesitate to contact us if you have any questions or would like to discuss the required revisions further. Thank you for the opportunity to review your work.

Best regards,

Nandita Basu, PhD
Consulting Editor, Communications Earth & Environment
Associate Editor, Communications Sustainability
Nature Portfolio

EDITORIAL POLICIES AND FORMAT

If you decide to resubmit your paper, please ensure that your manuscript complies with our editorial policies and complete and upload the checklist below as a Related Manuscript file type with the revised article:

For Manuscripts that fall into the following fields:
• Behavioural and social science
• Ecological, evolutionary & environmental sciences
• Life sciences
An updated and completed version of our Reporting Summary must be uploaded with the revised manuscript
You can download the form here:
https://www.nature.com/documents/nr-reporting-summary.pdf

For your information, you can find some guidance regarding format requirements summarized on the following checklist: (https://www.nature.com/documents/commsj-phys-style-formatting-checklist-article.pdf) and formatting guide (https://www.nature.com/documents/commsj-phys-style-formatting-guide-accept.pdf).

REVIEWER COMMENTS:

Reviewer #1 (Remarks to the Author):

The manuscript titled" Marine plastic pollution undermines the livelihoods and income of fishing communities in Vietnam" reports on the impact of plastic pollution on fisheries near two main rivers in Vietnam. The research focuses on the manatery cost, but also considers other impacts. The location utilises fishing communities that operate close to the estuaries of two very polluted rivers, the Mekong and Red Rivers. My understanding of the amount of work covered within this paper continued to grow as I read the paper. In addition to the above-named focus, the paper also includes waste audits on marine litter collected by fishers from the sea, and understanding from fishers on temporal and special hotspots.
I would like to highlight the importance of the work undertaken by the authors. They have focused on two primary gaps. 1) They have captured the socioeconomic impact of marine plastic pollution, with a quantifiable monetary value. I have been looking for such references for years, and would like to commend the authors for tackling this gap. Additionally, research within the global South and on the impact of plastic pollution is also an important area of work. Their supplementary work on litter collected by fishers at sea and fishers' understanding of hotspots is also interesting and fills similar gaps.
Having tried to find references on the measured cost of marine litter (further than cleanup costs), I consider this research to cover a very important gap, with interest from a wide range of readers and stakeholders. And I commend the Authors in their work on this. However, some additional work on the manuscript itself is required, particularly due to the layout and general readership of the chosen Journal. I need to be able to read and understand the paper before even getting to the methods. The abstract/intro, etc., is well-written, but it does not fully capture what has been done – I discovered new areas of inquiry in the results, such as the audits.
I think the presentation of the results needs to be revisited, e.g Figure 1. The written focus does not quite align with the figures. E.g. Figure 1d is the main result, but then the text focuses on other costs or the breakdown of costs rather than the differences between the fishing types (shown in Figure 1a-c). So the focus splits. The authors should consider revisiting the paper in light of their key results and focus. Additionally, the qualitative results (quotes) are highlighted as valuable but are not fully visible for full utilisation of the current form – I have given detailed comments on this within the results and discussion section.
Overall, the paper could benefit from restructuring to follow the focus of the paper. I have placed a detailed comment on this within the introduction section and then throughout.
I want to emphasise that the amount of work invested in this manuscript is truly phenomenal. Not just the use of a mixed-

method survey approach, but also the additional waste audit. I would really like to see this work published. Hence, I encourage the authors to approach the comments in the supportive manner they are intended.
Please see the attachment for my detailed comments.

Reviewer #2 (Remarks to the Author):

Comments on the manuscript by Nguyen et al.:

This study was conducted in three distinct areas of Viet Nam, where fisherman were interviewed for their perception and experiences regarding plastic waste pollution during their fishing activities. The authors also conducted a waste survey in these regions to determine plastic waste composition. In my opinion, the authors highlight essential aspects related to plastic pollution and its effects on coastal livelihoods by interviewing local fishermen and related authorities, which are often disconnected from traditional field surveys in scientific publications.

Overall, I found this work is relevant to the study field and that authors make a significant contribution, and that the level of detail provided is enough for this work to be reproduced and verified by other researchers.

I would like the authors to revisit their statement at the end of their introduction where they say they aim to “advance marine plastics research by focusing on three key areas”, and check whether these have been fully addressed in their discussion and conclusion. It really benefits the overall understanding of a manuscript to have such messages being recalled throughout the manuscript and backed up by your own evidence or by relevant literature as appropriate.

I found that the claim presented in the conclusions of how plastic unproportionally affects coastal communities like fishermen is very important. However, I believe the authors could make more use of similar previous work to place their findings in a wider regional or even global context. For example, I found very interesting that the authors saw that “the relative cost in terms of percentage of revenue loss was significantly higher for fishers in the Mekong Ben Tre province” than other study sites, which probably relates to their lower annual revenue when compared to the other two studied provinces. Is this observed in other parts of the world? Is there any indication in the literature of how strong the income factor is when defining effects of pollution, if compared to other socio-economic or environmental factors?

For another claim in their conclusions, the authors indicate that fishermen have a strong understanding of plastic waste pollution in their region and that this knowledge should be better explored in designing solutions. Again I find this an essential and very important finding that was could be more explored and strengthened by looking at and deeply comparing with previously published literature on the same subject.

Despite my confidence in commenting on peer work regarding plastic pollution, I highlight that I am not familiar with the methods of estimating economic impacts of plastic pollution and therefore cannot commented on their appropriateness. I leave that to other reviewers and editors who might be more familiar with those.

Other targeted comments from me are:
- I believe the overall text flow could benefit from eventual reminders that this study focussed on small scale fisheries, which is not made fully clear until the reader gets to the discussion of methods. This is specially important if authors believe that small and larger fishery vessels can have significantly different contribution to plastic pollution in their study region.
- I suggest the number of interviewees corresponding to the data is indicated in each figure.
- I saw from the results on the fisher knowledge that their findings on plastic waste is also a mix of fishery-related and consumer plastic items, as were the findings from the waste audit. I would like to see this distinction more evident throughout the manuscript as to indicate that not all plastic waste affecting fishermen, and maybe not even most of it, comes from fishery activities.
- I found the results indicating a potential intervention being a “fishing for litter” programme very valuable, i.e., “fishers indicated that they would need to be paid a median buyback price of 0.16 (0.08 - 0.23) USD kg-1 of marine plastic debris”. This type of information can be directly used towards mitigation measures, which are often hard to track. I suggest this finding is highlighted again in your conclusions.
- It was not clear to me whether the cost burden from fishing vessels in the different study sites was from perceived or actual cost burden. To me it would be useful to define this as readers will be more able to compare values with other studies.
- I found the reports of accidents related to marine plastic very alarming. Are there any reports of accidents in other regions of the world? I believe it is very important to emphasize that these accidents happen and are not isolated cases, and bringing other examples would really help.
- In the discussion, the authors say “The direct cost estimated in this study is 0.6 to 3.6 times different from previous estimates for other provinces in Viet Nam (See Supplementary Information T8). The variability in estimates from elsewhere is associated with local demographics and environmental context. In addition, previous studies often only accounted for direct costs (i.e., gear damage, repair cost, time spent sorting catch from plastic) and did not include lost opportunity cost 32, which we do account for (i.e. potential revenue from time lost that could otherwise be spent fishing, reduction in catch due to interference with gear).” Could the authors give more detail on other studies regarding their environmental and socioeconomical aspects that can differ from the present work? I think the reader needs to have more detail on the main manuscript, rather than having to go to supplementary table 8 to be convinced of this. Similarly, could the authors please elaborate a bit more on why the inclusion of this factor onto cost calculation is important?

I hope the authors find these comments helpful. My overall aim was to instigate critical thinking in order to better place this work in a wider context and enhance visibility and relevance once it gets published.

Best regards,

Lara Pinheiro PhD

Reviewer #3 (Remarks to the Author):

The manuscript, entitled “Marine plastic pollution undermines the livelihoods and income of fishing communities in Vietnam,” focuses on quantifying macro plastic pollution along the coast from the perspective of the affected fishing communities in three major fishing areas in Vietnam (Nam Dinh, Ben Tre, and Hai Phong).

The topic is fair. However, based on this consideration, the manuscript contains some findings that may contribute to the knowledge in the field. However, the manuscript quality is average; it contains some unclear points that the authors must address. The results and discussion section requires improvement, particularly in terms of a strong or deep interpretation of the results. Although the manuscript includes adequate sample sizes and distinct sampling groups, the authors did not conduct the analyses for these groups. This omission limits the depth and robustness of the findings, as subgroup-specific insights could have provided a clearer understanding of differences among the fishers. Some examples are below.
- Update figures (legends) for clarity and add essential figures or tables into the context for robustness checks on the results (for example: Figure 2)
The data descriptions are sometimes unclear, or the supplementary data is presented in a confusing way that makes it hard to understand.
- The data presentation also needs revision.
- Please check data line 94
- Please clarify lines 143-156:
- Please clarify lines 164-179: there are some mysterious numbers (didn't see from supplementary data T7).
- Unused data: TableS3

It is for this reason that I recommend this manuscript for a major revision.

** Visit Nature Portfolio's author and referees' website at www.nature.com/authors for information about policies, services and author benefits**

Communications Earth & Environment is committed to improving transparency in authorship. As part of our efforts in this direction, we are now requesting that all authors identified as ‘corresponding author’ create and link their Open Researcher and Contributor Identifier (ORCID) with their account on the Manuscript Tracking System prior to acceptance. ORCID helps the scientific community achieve unambiguous attribution of all scholarly contributions. You can create and link your ORCID from the home page of the Manuscript Tracking System by clicking on ‘Modify my Springer Nature account’ and following the instructions in the link below. Please also inform all co-authors that they can add their ORCIDs to their accounts and that they must do so prior to acceptance.
https://www.springernature.com/gp/researchers/orcid/orcid-for-nature-research

For more information please visit http://www.springernature.com/orcid

If you experience problems in linking your ORCID, please contact the Platform Support Helpdesk.

Version 1:

Decision Letter:

<*** REMEMBER TO ATTACH REVISIONS CHECKLIST (WORD)***>

** Please ensure you delete the link to your author home page in this e-mail if you wish to forward it to your coauthors **

Dear Professor Kaiser,

Your manuscript titled "Marine plastic pollution undermines the livelihoods and income of fishing communities in Vietnam" has now been seen by our reviewers, whose comments appear below. In light of their advice we are delighted to say that we are happy, in principle, to publish a suitably revised version in Communications Earth & Environment.

We therefore invite you to revise your paper one last time to address the remaining concerns of our reviewers. At the same time we ask that you edit your manuscript to comply with our format requirements and to maximise the accessibility and therefore the impact of your work.

EDITORIAL REQUESTS:

Please review our specific editorial comments and requests regarding your manuscript in the attached "Editorial Requests Table".

*****Please take care to match our formatting and policy requirements. We will check revised manuscript and return manuscripts that do not comply. Such requests will lead to delays. *****

Please outline your response to each request in the right hand column. Please upload the completed table with your manuscript files as a Related Manuscript file.

If you have any questions or concerns about any of our requests, please do not hesitate to contact me.

SUBMISSION INFORMATION:

In order to accept your paper, we require the files listed at the end of the Editorial Requests Table; the list of required files is also available at https://www.nature.com/documents/commsj-file-checklist.pdf .

OPEN ACCESS:

Communications Earth & Environment is a fully open access journal. Articles are made freely accessible on publication. For further information about article processing charges, open access funding, and advice and support from Nature Portfolio, please visit https://www.nature.com/commsenv/open-access

At acceptance, you will be provided with instructions for completing the open access licence agreement on behalf of all authors. This grants us the necessary permissions to publish your paper. Additionally, you will be asked to declare that all required third party permissions have been obtained, and to provide billing information in order to pay the article-processing charge (APC).

Please use the following link to submit the above items:
Link Redacted
** This url links to your confidential home page and associated information about manuscripts you may have submitted or be reviewing for us. If you wish to forward this email to co-authors, please delete the link to your homepage first **

We hope to hear from you within two weeks; please let us know if you need more time.

Best regards,

Nandita Basu, PhD
Consulting Editor, Communications Earth & Environment
Associate Editor, Communications Sustainability
Nature Portfolio

REVIEWERS' COMMENTS:

Reviewer #1 (Remarks to the Author):

This report is a second round review by Reviewer 1. The reviewer was also tasked with considering whether Reviewer 2's comments were adequately addressed. Thus, this review considers both reviewer comments.
Reviewer 1 comments
Please find attached a revised copy of the paper with minor grammatical corrections – these were things I noticed while reading it. It was easier to make the change directly in the track than to write it out for such small changes. Please note these are minor changes.
Please note you also switch between when to use the "-", e.g. mixed-method or mixed methods. You need to check this and other such consistencies throughout the paper.
Abstract:
Line 21/22, please put in the type of interview.
• i.e., your response in green below the names of the survey – this should be in the abstract as well. Keep the words

“structured and post-hoc”, but add “the Economic cost survey” and the “validation survey” wording.
• I also think it is important to include that both surveys are quantitative and qualitative.
“We revised throughout the manuscript on how we refer to the two surveys period as “The economic cost survey” and “The validation survey”. Both surveys are structured interviews, where we meet the fishers in person and interview them for about 45 minutes based on a set of interview questions (available in SI).
These interview questions consisted of both questions to collect quantitative data and qualitative data.
Quantitative data are defined as data that can be measured in numbers; for example, on average how much time do you spent cleaning marine debris from fishing net in each haul; how many people were typically involved in that process; and Likert-scale questions: what is your level of agreement to the following statements (5 options ranging from 1-very disagree to 5-very agree)). “

Highlights
Point 2”- Consequences of marine plastic litter cost each fishing vessel >10% of the annual revenue and 25% of annual income for vessel owners.”
• My understanding from the abstract is that this is annual revenue for individual fishers. From here and the paper, that it’s >10 for annual revenue per boat for the fishers. Do you mean each fisher's individual income or the annual income of the vessel? Can you just make sure this is clear here and throughout the paper? Currently is appears at odds with the abstract. Please check the wording and clarify in text where relevant. I think the issue is really in the abstract wording.
• Additionally, this isn’t quite right. Neither here nor in the abstract, your results show a 7-18% range between the different sites. I would put either the range, the 12% or both into the abstract and highlights rather than 10%. I prefer both. But I do not like the use of ~ given that you have 12% and a range. I would apply the same change to the annual income for vessel owners. Apologies for not identifying this in round 1. Essentially, I am asking you to change the abstract and highlights to what you have in the main paper, i.e., “This amount is equivalent to 12 (7 – 18) % of their annual revenue or 25 (15 - 48) % of the fishing vessel owner’s income”

Results
It is still important to clarify whether your weight is dry or wet weight. Please clarify in the main text. I know you have it in the SI.
A note for your own future research – we really do need the clean and dry weight values. I know it's an added logistical and personnel issue, but we do need it. I think, in this case, because it also affects a fisherman's ability to haul and land litter, your use of wet weight is arguably acceptable. But for comparisons with other marine litter data, the clean and dry is becoming more important.

Methods
I note that you do not include the reference to the Likert Scale for the quantitative scale in your methods. Reference to it needs to be in the main paper.

Under data collection:
It is important to clarify that both surveys used a mixture of quantitative and qualitative questions, including Likert-scale and open-ended questions ect. Please include.
Under waste audit: “A waste audit was conducted on board fishing vessels by observers following the method adapted from GESAMP, 201954.”
What adaptations did you make? This is easily answered by adding a reference to SI 1.3
SI
SI 1.1.
Again, please include a sentence on the Likert scale used in qualitative research. Currently, you only have it in the table lower down.
Please clarify how many researchers conducted your interviews. If more than one, mention training/discussion to prevent influencing the interview through different conductors etc.
Your questions are not in the SI – please add them. I see they are with the raw data, but they need to be in the SI. I am presuming this is because the SI is currently in a word doc and you will combine the documents when they are in PDF form - but your contents table in the word document does not make this explicit. Thank you for including both languages.
Comments on reading the rebuttal:
Thank you for adding uncertainty ranges.
Thank you for expanding the SI.
Thank you for adding the raw dataset as well – it is always good to have that publicly available.
I respect your decision not to include the grouped qualitative results table in the main paper. Despite my request. I can see why you have made this choice. However, I am noting that you are underutilising this data set in this paper. I work mostly quantitatively with some qualitative work, and I can tell you that if I were my qualitative colleagues, they would not allow this through review. However, as you have so much data, and have used key quotes, I respect your decision. Thanks for adding a partial thematic table to the SI.
I appreciate the difficulty of balancing such a wide range of datasets and results between the main and SI, and though you disagreed with some of my recommendations in round 1. I think the changes you have made are good.
“This paper focuses on fishers’ perspectives and knowledge of marine plastic pollution impacts, rather than technical comparisons of waste audit data, which will be addressed in a separated forthcoming paper.” – I look forward to seeing that paper, I will look out for it.

While injuries and fatalities related to marine plastic pollution do occur in practice, such incidents were not widely or

frequently reported by fishers in our study (total 3 out of 199 fishers interviewed). – this is still a high number. Just a comment too your comment. No change required.

Reviewer 2 comments

I agree that the authors have strengthened the paper in response to Reviewer 2's points. I am only commenting on the points which may not have arguably been 100% covered by the authors.
2.4 – I agree with the authors that the literature is limited to support this request, and believe that they have covered it adequately, given what is available and the gap they are addressing.
2.9 – To be honest, I could not see where in the method section this had been clarified. However, I think it is clear now, throughout the manuscript, and in other changes that this is a calculated cost. Thus, I believe this comment is adequately covered.
2.10 – I believe the reported references are adequate, and agree that you do not need to add the news article, as you have the journal references. However, as the authors identified, the references cover other nations, not Viet Nam. Given that, I probably would have included the news reference out of interest. However, I believe the authors have adequately addressed the point.
2.11 – The authors have expanded on this. The initial reviewer may potentially have been thinking of a core comprehensive expansion; however, I believe the succinct expansion provided is good.

Reviewer #3 (Remarks to the Author):

I am satisfied with the authors' responses to the questions and issues raised in my initial review.
The revised manuscript is clearer and easier to follow after incorporating the reviewer's comments.

Some minor comments
1. The minor issue relates to grammatical check.
2. Why fishers may overestimate debris quantities? (Line166)
>> Please provide the further explain
3. Clarification of whether sensitivity analysis was conducted to evaluate uncertainty of the cost estimation analysis?
4. Figure captions are too detailed but could be shortened for readability.

** Visit Nature Portfolio's author and referees' website at www.nature.com/authors for information about policies, services and author benefits**

Open Access This Peer Review File is licensed under a Creative Commons Attribution 4.0 International License, which permits use, sharing, adaptation, distribution and reproduction in any medium or format, as long as you give appropriate credit to the original author(s) and the source, provide a link to the Creative Commons license, and indicate if changes were made.

In cases where reviewers are anonymous, credit should be given to 'Anonymous Referee' and the source.
The images or other third party material in this Peer Review File are included in the article's Creative Commons license, unless indicated otherwise in a credit line to the material. If material is not included in the article's Creative Commons license and your intended use is not permitted by statutory regulation or exceeds the permitted use, you will need to obtain permission directly from the copyright holder.
To view a copy of this license, visit https://creativecommons.org/licenses/by/4.0/

Point-by-Point response to reviewers

1. Reviewer #1:

We sincerely thank the reviewer for their detailed and constructive feedback, and for highlighting the novelty and importance of our findings that fill the important knowledge gap on quantifiable socioeconomic impact of plastic pollution.
We appreciate that the reviewer has raised a set of interconnected comments relating to narrative focus, method clarity, and result interpretation. In response, we have grouped them into thematic responses, each of which addresses several comments together. This allows us to summarise our response more clearly and avoids repetition, while ensuring that every comment is acknowledged and fully addressed. Please find our responses below with the track changes in the revised manuscript.

Overview Response to Main Thematic Comments:

1.1. **Refine research gap statements and highlight contribution to the economic dimension of plastic pollution (orig. 1,10,19,20,24,33,36,37,41,47,48):**
Response: We have revised the Abstract and Introduction to more clearly articulate the study's novelty in quantifying the economic burden of marine plastic pollution on small-scale fishers. The research gap is now presented through concise, focused statements that lead directly to the study objectives. We have also added uncertainty ranges and clarified sources of variability in the economic estimates to strengthen the robustness and transparency of the findings.
1.2. **Improve presentation coherence between figures, tables, and text (orig. 12,13,25–28,31,33,36,37,43):**
Response: We have restructured the Results section so that the narrative follows the sequence of key findings in order and analytical logic of the figures and tables. All Figures, Tables, and Supplementary Information are now explicitly introduced, interpreted, and directly linked to the text, ensuring that figures support the written argument and that key results are clearly supported by detail data. All key findings and quantitative results are presented in the main manuscript. The Supporting Tables in the Supplementary Information provide additional detail, including statistical test results and breakdowns by location and gear type. Where significant differences among locations or gear types were identified, these are highlighted in the main text; otherwise, the results are reported in the Supplementary Information. This approach streamlines the manuscript and maintains focus without overloading the text with detailed data.
1.3. **Clarify methodological transparency and data collection design, especially waste audit (orig. 13,15,16,26,27,31,32,43,45,50,52,53,55,59-62):**
Response: The Methods section has been substantially expanded to clarify the survey design, sampling strategy, vessel types, and multi-stage data collection process (Figure 5). We have included the full interview forms and raw dataset in the Supplementary Information to improve reproducibility and data transparency. A short

methodological recap has been added to abstract and at the beginning of the Results section. The waste audit is now introduced earlier, summarized more clearly, and supported by visual representation to integrate it fully with the study's core aims.

1.4. **Integrate qualitative evidence more systematically (orig. 14,17,21,22,29,30,42): Response:** Qualitative quotes are now grouped thematically in Supplementary Table 7 and integrated within the Discussion to directly support interpretation of the quantitative results. We have selectively added qualitative insights and discussion to complement with the quantitative data to highlight the safety risks reported by participants and perceptions of fishers on solutions for marine plastic pollution.

1.5. **Strengthen structure and logical flow of Results and Discussion (orig. 10,25–27,31,36,47,48):**

Response: The Results and Discussion sections have been reorganised to follow a clearer analytical pathway, ensuring that conclusions are directly supported by the presented evidence. Minor revisions to the Introduction were made to align with this new structure.

Detail point by point responses:

Original comments:

The manuscript titled" Marine plastic pollution undermines the livelihoods and income of fishing communities in Vietnam" reports on the impact of plastic pollution on fisheries near two main rivers in Vietnam. The research focuses on the manatery cost, but also considers other impacts. The location utilises fishing communities that operate close to the estuaries of two very polluted rivers, the Mekong and Red Rivers. My understanding of the amount of work covered within this paper continued to grow as I read the paper. In addition to the above-named focus, the paper also includes waste audits on marine litter collected by fishers from the sea, and understanding from fishers on temporal and special hotspots.

I would like to highlight the importance of the work undertaken by the authors. They have focused on two primary gaps. 1) They have captured the socioeconomic impact of marine plastic pollution, with a quantifiable monetary value. I have been looking for such references for years, and would like to commend the authors for tackling this gap. Additionally, research within the global South and on the impact of plastic pollution is also an important area of work. Their supplementary work on litter collected by fishers at sea and fishers' understanding of hotspots is also interesting and fills similar gaps.

Having tried to find references on the measured cost of marine litter (further than cleanup costs), I consider this research to cover a very important gap, with interest from a wide range of readers and stakeholders. And I commend the Authors in their work on this. However, some additional work on the manuscript itself is required, particularly due to the layout and general readership of the chosen Journal.

1. Overall

I need to be able to read and understand the paper before even getting to the methods. The abstract/intro, etc., is well-written, but it does not fully capture what has been done – I discovered new areas of inquiry in the results, such as the audits. I think the presentation of the results needs to be revisited, e.g Figure 1. The written focus does not quite align with the figures. E.g. Figure 1d is the main result, but then the text focuses on other costs or the breakdown of costs rather than the differences between the fishing types (shown in Figure 1a-c). So the focus splits. The authors should consider revisiting the paper in light of their key results and focus. Additionally, the qualitative results (quotes) are highlighted as valuable but are not fully visible for full utilisation of the current form – I have given detailed comments on this within the results and discussion section. Overall, the paper could benefit from restructuring to follow the focus of the paper. I have placed a detailed comment on this within the introduction section and then throughout. I want to emphasise that the amount of work invested in this manuscript is truly phenomenal. Not just the use of a mixed-method survey approach, but also the additional waste audit. I would really like to see this work published. Hence, I encourage the authors to approach the comments in the supportive manner they are intended. Please see the attachment for my detailed comments.

Overall focus

I give further details below. Please note that my approach is to write my detailed comments in order of my thoughts, like a flow of consciousness. This means that I might raise a point earlier that you deal with later. If this happens, I think you should consider moving the latter section earlier or mentioning it briefly and indicating that it is dealt with in detail later.

Detailed comments:

- Title: I like the title – it's to the point and includes all the necessary information.

Response: Thank you very much for your thoughtful and constructive feedback. We have carefully refined and restructured the flow of the manuscript to reflect the focus of the study. Based on your constructive comments, we have highlighted the waste audit integration, key findings from mixed-method survey, visibility of qualitative data to improve the clarity and flow of the manuscript. Detailed point-by-point responses to the comments are included below and highlighted in the revised manuscript.

2. Abstract

The abstract is well written. It captures the key points of the paper. I appreciated both the monetary value and how you then put it into the framework of the overall revenue and income. Honestly, one of the best abstracts I have read in a while.

Small changes:

-You speak of 2 rivers and 3 study regions. From the abstract alone, it's unclear whether you conducted two study regions near one river or one study region by each river, along with a control further away.

-Line 20: "We found that the total incurred costs" Please change to "we calculated that…" I initially read it that way, as the fishers were telling you that cost, rather than what you calculated.

Returning to the abstract and highlights after reading the paper, I still really like your abstract and highlights. BUT, and this is important, you make no mention of the waste audits in either. You are unlikely to have the words available for the abstract, so please work the waste audit work into your highlights. You may be able to include the waste audit in the second line of your abstract – have a look.

Response:

- We conducted the survey at the outlets of the 2 rivers. According to the governance structure in Vietnam, the study locations were in three provinces/cities (Hai Phong City and Nam Dinh Province at outlets of the Red River; and Ben Tre Province at outlet of the Mekong River) We have removed the word “three” in “three study regions” to avoid confusion.
- We changed to “we calculated that” as suggested.
- We added a sentence to clarify our methods and including the validation survey with 94 fishers and 282 waste audit observation: “We used a mixed methods design to quantify impacts of marine plastics based on structured interviews with nearshore

fishers (n = 199) and subsequently verified these findings through additional fisher interviews (n = 94) and waste audits of marine plastics caught in fishing nets (n = 282) in the same study areas”

3. Keywords: Please check the keyword requirements of the paper. I think you have too many. I use the rule of not using a keyword if that word is in my title

Response: Thank you for the suggestion, we have updated our keywords according to the journal’s requirements.

4. Highlights: Not as strong as your abstract – but fine. I would revisit once you have restructured the paper to really highlight your key takeaways.

Response: Thank you, our highlights summarised the key findings of our research.

5. Introduction: Overall, your introduction is succinct. However, it could improve with slightly more information – especially given the general audience of the Journal you have chosen. I recommend bringing in a few lines on the socioeconomic impacts we do know on marine litter (globally and regionally, before you consider locally). You touched on the local impacts in theory slightly in line 61 as a gap. However, I would also include other socioeconomic aspects that we know or expect to be impacted by marine litter, and then highlight the significant monetary gap affecting local fishers. I know that much of the socioeconomic impacts of marine litter have been more theoretically conceptualised for years rather than measured outcomes (yours is a measured outcome), and I really think you can highlight that gap that you fill within the greater socioeconomic space.

Response:

We added a few background and global data on socioeconomic impacts of marine litter to provide context and highlight the gap in existing theoretical/assumption based estimation models at global scale.

We added to the first paragraph of the introduction the following sentences: “*The lifetime socio-economic cost of marine plastic pollution in 2019 was estimated at 3.7 trillion USD and is projected to exceed 7.1 trillion USD by 2040 [15]. These costs were modelled based on quantifiable costs from greenhouse gases, waste management, and impacts of mismanaged waste on marine ecosystem. Globally, the direct economic impact of plastic pollution on tourism, fisheries, and governance along coastlines and waterways was 2 USD per capita per year, with 82% attributed to cleanup costs and 18% to revenue loss[16]. Nevertheless, we currently lack an understanding of the socioeconomic impacts of this plastic pollution on coastal communities with is primarily informed through modelling that do not accurately quantify the unseen impacts on communities and their well-being [17,18]. Here, we address the key knowledge gap in existing macro-scale estimation models by providing empirical based costs of marine plastic debris on the incomes and livelihoods of fishing communities.*”

6. Line 48 -50 – I know and appreciate the local issues that drive plastic pollution rates in Asian countries. However, you need to include the global issues surrounding

the plastic waste trade in the region, which is driven mainly by Western countries. It's an important contribution, and needs to be added here.

Response:

Thank you for the recommendation. To provide context of this global plastic challenge while still keep the introduction short, we added reference and changed this sentence to: *"Even though the plastic pollution is a global issue, the plastic waste trade[14] to Asian countries with high coastal population density and the associated plastic leakage rates render this region as the one that bears the highest "price tag" of plastic pollution which is estimated to be 11 billion USD per year[15]."* Additional discussions on plastic pollution inequities are also available in the discussion section below.

7. Line 56 – I found it interesting that you used Vietnam in the title and Viet Nam in the main text. And to be fully honest, I read into the difference and I learnt something new here. I actually appreciate the point you have made by using Viet Nam. And can see why you still utilised the English form in the title. It could be argued that you should either use Vietnam or Viet Nam throughout. Personally, I respect the choice you have made – and I presume a Vietnamese author led the decision.

Response: We revised to consistently use the name "Viet Nam" throughout the manuscript.

8. Line 64 – please remove "(See Supplementary Information F1 and methods)" – make sure it's in the methods, but do not have it in the introduction.

Response: We removed "(See Supplementary Information F1 and methods)".

9. Line 69 – 71. Your introduction perfectly supports your point 1. Your points 2 and 3, however, are not. You need to make adjustments for this. However, I will come back to this after reading the whole paper.

Response: We revised the sentence accordingly to: "(2) *limitations of macroscopic economic models that reply on assumptions and overlook empirical data from impacted communities.*"

The sentence in the introduction at the end of the first paragraph in the Introduction: *"Nevertheless, we currently lack an understanding of the socioeconomic impacts of this plastic pollution on coastal communities with is primarily informed through modelling that do not accurately quantify the unseen impacts on communities and their well-being [18,1918,19] . Here, we address the key knowledge gap in existing macro-scale estimation models by providing empirical-based costs of marine plastic debris on the incomes and livelihoods of fishing communities."* supports this knowledge gap mentioned in point 2 and 3.

10. Comment after reading the full paper: I am not happy with your introduction after reading the whole paper. And I think this comes from the fact that you have collected so much information (a tremendous amount), but then in the different sections

(abstract/intro/results/discussion), you have focused on different sections of information. Even between your figures and written text, there is a disparity. You need to decide what your primary and secondary results are. I would like to see you report it all; however, I need you to restructure each section carefully to show the move from primary to secondary results. Or to word it another way, list the order of your results in order of priority (most important at the top) and then restructure your entire paper to reflect that. Then, return to your introduction and include the necessary background information for each piece of information you have collected.

Response: We have restructured and revised the manuscript to enhance coherence between sections and between the figures and text. The abstract and introduction were revised accordingly to reflect the method and highlight the key results in order of priority (see the Highlights).

We also summarise the key results for this research paper as:

- Based on interviews with 199 fishers, we calculated that the total incurred costs of plastic pollution in the study regions were ~3400 USD per vessel per year, which is equivalent to more than ten percent of the annual revenue and approximately a quarter of the income of the vessel owners. These estimates do not include reported injuries and fatalities when dealing with plastic entanglement incidents at sea.
- Our results highlighted the disproportionate economic burdens faced by lower-income nearshore fishers in the Mekong Delta compared to those in the Red River Delta.
- We found that fishers have a high level of knowledge of marine plastic hotspots and a strong willingness to take collective action to mitigate marine plastic pollution.
- These findings were corroborated by additional interviews (with 77% of 94 newly interviewed fishers agreeing with the economic assessment), and by waste audits (n = 282), which showed plastic weights caught in nets were only three times lower than fishers' estimates in the same study areas.

11. Line 69 -71 "(2) limitations of macroscopic models that rely on assumptions and lack empirical data from the impacted communities, and (3) potential of local perceptions to enhance such models, a factor that is often overlooked." I would roll this under one point

Response:

We revised the sentence to: "(2) limitations of macroscopic economic models that rely on assumptions and overlook empirical data from impacted communities."

Results

You have some truly fascinating results. But you need to work on some recurring issues:

12. There is a misalignment between your focus on your figures and the focus on the text. This needs to be addressed.

Response: We revised to increase the alignment between the figures and text throughout the revised manuscript.

13. Much of your main results figures/tables are in the SI. This is an issue.

Response: Supplementary Tables in the SI are intended to provide detailed data and statistical analyses for the main results presented in the manuscript. The key findings are visualised in Figures 1 and 2 and highlighted in the written text. Due to the large dataset from mixed method study design, we deliberately focus the main manuscript on a limited number of core results, while presenting additional detail, nuance, and supporting analyses in the SI and accompanying dataset to maintain clarity and readability.

14. Although you have included standalone quotes within the results, this approach somewhat diminishes the impact of your qualitative findings. I would recommend including them all (in text and in SI in a table divided into themes). For this, I recommend looking up the latest socioeconomic work on microplastics – specifically papers that have utilised both quantitative and qualitative methods, which provide examples of such tables.

Response: We have a collection of quotes categorised into themes in Supplementary Information Table 7. Additional quotes were added in the SI-Table 7 and in the text to better highlight the quantitative results of our study.

15. You haven't fully captured the requirements of the layout of the Journal here. I would include a brief summary of your approach here at the start of the results (1-2 lines) before going into the results. I see examples from the latest articles vary in this approach. But I recommend it, e.g. https://www.nature.com/articles/s43247-025-02670-x . Remember, the layout of this Journal requires me to be able to read the results without a detailed understanding of the methods (I only read them later). Thus, I need to know the differences between the sites, and what your values mean.

Response: We moved Figure S1 from the Supplementary Information to the main manuscript (Figure 5 in revised manuscript) to provide a clear illustration of the survey process.

We also added brief summary of the Method before the Results section to align with the layout of the Journal and improve readability for readers: *"This study used a mixed-methods approach, including structured interviews with fishers (n=199), focus group discussions (n=3 with 8 participants each), and semi-structured interviews with port managers (n=6) and fisheries officers (n=9) across three nearshore regions of Hai Phong, Nam Dinh, and Ben Tre, at the outlets of the Red and Mekong Rivers in Viet Nam (Figure 4 and 5). Reported data were analysed to estimate the economic and societal costs of marine plastic debris on small-scale nearshore fisheries. The socio-economic impact assessment and fishers' ecological knowledge of marine plastic pollution were further complemented by validation surveys with different fishers (n=94) and waste audits of debris caught in fishing nets (n=282) in the same study areas."*

16. Line 76 (See Methods and Supplementary Information for methodological details). In line with the above comment, I would remove this. The reason this becomes an important point is that I initially thought your first results paragraph was discussing the amount of money the fishers claimed it was costing them. But I can see from the SI that this is not the case. Instead, it is the total cost you calculated – that's important, and it needs to be clear here.

Response: We have removed “(See Methods and Supplementary Information for methodological details)”.

17. You have a good blend of quantitative and qualitative, and have incorporated the quotes nicely. However, as you have utilised the qualitative, you could take this further. I have written elsewhere how. Further to my remarks on this point, please consider if you can **take the qualitative results still further**.

Response: Thank you for your comments. Due to limitation on word count and to keep the focus of the paper, we decided to selectively include quotes and qualitative results that enhance the study findings in the main manuscript. We added relevant qualitative results in the SI.

18. "Error!source not found" in the PDF but not the Word version – so an error in journal processing. Just be aware.

Response: We checked the PDF of the revised manuscript has been correctly processed without this type of error. Please note that this type of error could appear when the submission system automatically converts the Word file to PDF file.

19. Line 83- 92 – need to name this figure/graph where these numbers come from. I think the detail here is important. Additionally, it appears that these values are not derived from figures in the paper. They are the main results, and so the figure/table should be in the main paper.

Response:

Thank you for this comment. The values reported in Lines 83–92 represent intermediate results used to contextualise and provide additional detail for the cost estimations presented above, rather than standalone primary outcomes. To maintain readability of the main manuscript and avoid presenting extensive numerical detail in the text and again in the figures/tables, we report only the key summary values here and the detail data are presented in Supplementary Information.

To ensure transparency and traceability, we have now explicitly indicated their data source in the manuscript (“See data in Supplementary Table 1 and 4” in this section). This approach allows interested readers to access the full dataset while keeping the main paper focused and concise.

20. Line 95 – So here you report these results to be in the supplementary table- as the results are in the results section, I would argue these results should also be in a figure/table in the main paper. Results from supplementary graphs/tables should not really be focused on in the results, but should be used if mentioned only as supporting information in the discussion.

Response: In the main manuscript, the written text focuses on the key results and average data. The Supplementary Tables provide additional detailed results grouped by location and gear to offer further contextual information. As no significant differences were found among locations or gear types, the data are presented in Supplementary Table 4.

21. Line 99 – 104. Given this is a socioeconomic study utilising both quantitative and qualitative results, please consider creating a table of quotes supporting different themes. Then use quotes such as this in the text within the discussion.

Response: We have additional quotes supporting different themes in Supplementary Table 7.

22. Line 99-104. These are very good, expressive quotes. They do truly capture the human cost and tragedies involved. This could not have been easy work to explore, capture and report. I would like to express my respect for the resilience of both the fishers and researchers involved with this work. Also, again, showing the human cost here is a key result which is often only expressed in theory within the scientific literature. I think you can take the qualitative work further. It's a key part of this work.

Response: Thank you for your comment. We selectively include relevant qualitative data to keep the focus of the study.

23. Line 99-120 – I think it would be good to revisit your highlights to include some of the qualitative results captured here. You have it in the abstract, but I would highlight as well.

Response: Thank you for your comment. We selectively include relevant qualitative data to keep the focus of the study.

24. Line 105- 111. This is an interesting result in line with your quantitative work.

Response: We added "*These results also align with the findings from the quantification of …*"

25. Figure 1. The figure is a good figure. However, the written focus does not quite align with the figure. E.g. Figure 1c/d is a main result, but then the text focuses on other costs or the breakdown of costs rather than differences between the fishing types (shown in Figure 1a-b). So the focus splits. The authors should consider revisiting the paper in light of their key results and focus.

Response: Thank you for this helpful comment. The purpose of Figure 1 is to provide supporting detail on cost breakdowns by gear type and location, while the main text

focuses on overall patterns across locations. We have revised the text to better align the narrative with the core results shown in the figure, particularly by clarifying how cost differences vary across fishing types, and have streamlined the discussion of cost components so they support rather than distract from this central comparison. To maintain conciseness, we avoid repeating interpretation of all figure elements in the main text.

26. Line 120 –"Plastic debris composition based on fisher knowledge and waste audits" I got very excited when I saw this section, but I didn't know from your abstract/highlights nor intro that you did this. It is a problem that this came as a surprise.

Response: We have revised to include it in the abstract and beginning of the result section

27. Line 123 – I know you have limitations on figures within the Journal. But I really do not like that you refer to the supplementary results for what you present as key numbers. You need to revisit this.

Response: The Supplementary Tables in the SI provide detailed data broken down by location and gear type. As this section focuses on overall patterns, detailed breakdowns are presented in the Supplementary Tables.

28. Figure 2: This is a good figure. But again, you have data in here not mentioned in the text, which is interesting, ie the gill net vs trawl. Also, Figure2c - is it dry or wet? I presume dry. (Theres a link here with a comment in the methods – please see that comment)

Response: Similar to Figure 1, Figure 2 is intended to provide a detailed breakdown of marine debris caught in fishing nets across locations, gear types, and seasons. To maintain focus, we avoid reinterpreting all data shown in the figures again in the written text. We also cannot make direct comparison between trawl nets and gillnets because differences in fishing techniques result in non-comparable standardised catch-per-unit-effort metrics. This paper focuses on fishers' perspectives and knowledge of marine plastic pollution impacts, rather than technical comparisons of waste audit data, which will be addressed in a separated forthcoming paper.

Figure 2c and 2d present seasonal averages across two seasons, which is now clarified in the figure caption. Detailed data underlying Figure 2 are provided in Supplementary Table 2.

In the revised Figure 2 and Supplementary Table 2: We changed the position of data chart for Trawler to Fig 2a and Gillnetters to Fig 2b to be consistent with the order in Figure 1.

29. Line165 – 171: These numbers do not come from SIT7 – where do they come from? I would like to see the relevant quotes in T7 incorporated into the main paper. Also, do you have more than one quote per point? It would be helpful to include.

Line 172: I would like to see you take the quote usage further – include a table in the main paper showing the key quotes under the different themes. I.e., take all the standalone quotes and the quotes in the SI (and any others you have, so that more than one supports each point), and combine them into one table under the different points/concepts/themes.

Response: Relevant quotes for each theme are provided in Supplementary Table 7, with key quotes presented in the main text. To maintain focus in the main manuscript, we selectively include quotes most relevant to the topic. In response to the comment, we have added several additional quotes.

30. Line 174 – interesting but again…I don't like that the table is in the SI. You need to consider a full overhaul of your figures/tables between the main paper and SI.

Response: The Supplementary Table in the Supplementary Information provides results broken down by location and gear type to support the key findings presented in the main text. We have revised the manuscript to present key results as figures in the main manuscript, with detailed supplementary data provided in tables in the Supplementary Information and raw data files.

31. Line 181: Both the initial and follow-up surveys – presuming the first was a quantitative with the follow-up qualitative – so a convergent design. Not clear at this point. Additionally, this paragraph should be supported with numbers and quotes.

Response: Our mixed-methods study was designed as a sequential process rather than a convergent design. We have revised the Methods section to clearly describe the sequence of data collection and analysis and moved the methodological illustration to Figure 5 in the revised manuscript to improve clarity. The follow-up survey was intended to assess fishers' agreement with the socioeconomic cost estimation findings; to maintain focus and improve clarity of the Results section, we have removed this paragraph from the revised manuscript.

Discussion

32. Line 190 "Our findings demonstrate the substantial negative impact of marine plastic on the income, livelihoods and safety of nearshore trawl and gillnet fishers." I agree. But I also think this under-sells your results. Maybe I am still reeling from your quotes regarding the injuries and fatalities. Honestly, I think you can make this sentence stronger. And I would. "Safety" is a very safe word to use. Also, I would bring in a summary line on the site difference here – that is significant.

Response:

We have extensively revised the Discussion accordingly. While injuries and fatalities related to marine plastic pollution do occur in practice, such incidents were not widely or frequently reported by fishers in our study (total 3 out of 199 fishers interviewed). Fishing is widely recognised as a high-risk occupation with a high baseline level of accidents and fatalities, both with and without the involvement of marine plastic pollution. Therefore, we take a conservative approach in our conclusions and focus on the economic losses experienced by fishers until more robust evidence on incident frequency becomes available.

33. Line 191 "the cost": start a new paragraph.

Response: We made the edit accordingly.

34. Line 193: refer to figure

Response: we have added “(Figure 1d)” at the end of this sentence.

35. Line 203: You have discussed regional differences first. But your opening discussion line 190 does not highlight this as the most key result. I have in fact asked you to include a summary of the regional difference within your first lines before suggesting breaking into a second paragraph. This highlights to me that you agree – the regional differences are interesting but are not the key results. I recommend you reshuffle your results with this in mind. You have two potential choices: either incorporate regional differences into each discussion part or move this section down.

Response: We have revised this section to include a comparison of economic costs between Vietnam and other regions, as well as across different gear types and locations.

36. Line 217: So I see the economic costs you have defined, and this result is your key result.
Line 219: "often did not witness the incidents directly" interesting. But some did right? These are not urban myths. I read this as a few have directly witnessed these events. But be Careful of your wording here, as you cast doubt on that understanding. Be exact in what you mean.

Response: we changed ‘Although marine plastic-related accidents are rare and fishers often did not witness the incidents directly …’ to ‘Although marine plastic-related accidents are rare and not all fishers directly witnessed the incidents…’.

37. Line 232 Table T8. Thanks for this summary table. It is useful. And I agree with its placement in the SI.

Response: Thank you for the comment.

38. Line 229 – This is an euphemism. I laughed at how carefully you worded that, especially given the size of the gaps in the field. Not a comment to make a change.

Feel free to make the sentence stronger if you'd like to emphasise the importance of your work.

Response: We changed Lines 229-232 in the previous version to 'The current research on the socioeconomic impacts of marine plastic in Viet Nam and elsewhere is scarce and lacks standardized data collection approaches[30]. In addition, previous studies often only accounted for direct costs (i.e., gear damage, repair cost, time spent sorting catch from plastic) and did not include lost opportunity cost[32], which we do account for (i.e. potential revenue from time lost that could otherwise be spent fishing, reduction in catch due to interference with gear). These gaps highlight the important value of the estimates generated from this study.'

39. Line 235; "In addition, previous studies often only accounted for direct costs…and did not include lost opportunity cost32, which we do account for", I think this is a really important part of your paper. I think you should emphasise this point more in the introduction. Also, I would actually move this point up (to line 229), opening this whole section with this point. It gets lost here. But it's important.

Response: We have addressed this comment accordingly in the revised manuscript in the section "Lack of comparable data among socioeconomic impact assessment studies" (line 290-303 in revised manuscript).

40. Line 243-244: I agree. But I would like you to take your qualitative work further. In addition to the table of quotes I have suggested for your results section, I would then move all the standalone quotes you currently have in the results and weave them into the relevant discussion sections. You have done this slightly with line 172. But I would like to see it done with the others as well. I.e., I believe you can include all the quotes (without exceeding the word count, as they will be presented in a table) and then use the key ones you identified within the results to support your discussion itself.
Response: We added the Quotes by thematic topics in the Supplementary Table 7. A few key quotes were also added to the main manuscript where appropriated.

41. Line246-251 are important, and I like how you have written it. But I would argue this is an aside, and not a main focus of your results…. the socioeconomics is a main result and a key result. The regional differences are interesting and important…and then you bring in the waste Audit work – which I love, BUT I wasn't expecting from your abstract/highlight/intro. If you fix this in your intro, that should solve the issue of line 246-251 not truly being in focus.

Response: The paragraph from line 245-251: " Identifying spatiotemporal hotspots … bridge these knowledge gaps and enhance hotspot detection." provides context to our discussion on the solution pathway to address the marine plastic pollution problem: by

combining local knowledge in hotspots detection and actively engaging and supporting coastal communities in taking actions (for example, through the “fishing for plastic” initiative. We have revised our Abstract, Highlights and Introduction to provide context to this results and discussion section.

42. Line 249 – LEK? I think the full is only in the SI? Please double-check and fix if needed.

Response: LEK stands for ‘local ecological knowledge’ (line 138 in the submitted version).

43. Line 254 – again…you have such a wealth of knowledge in here – I didn't expect this result to creep in as something you looked at. I would greatly appreciate it if you could write a concise summary of your methods at the beginning of your results. This wouldn't be needed in a journal that places the methods at the end. But it is needed here.

Response: We have added the summarise of the method at the beginning of the Results section. Please see response to comment 1.15.

44. Line 257 "fishers’ knowledge of plastic distribution reflects reality”. I would argue it's the other way round – that the predictions reflect reality because they are supported by the fisher's knowledge. In fact, I feel strongly about that. Please change.

Response: Thank you for your suggestion. We changed this sentence to: “Results from the waste audit (Figure 2) and surveys with fishing communities (See Supplementary Table 5) were aligned, indicating that local knowledge was a valuable and reliable source of information to identify different plastic pollution hotspots of locations, seasons, items and potential polluters.The scientific reliability and accuracy of fishers’ knowledge of plastic distribution is also reflected by the fact that the reported hotspots (See Supplementary Information T5) agreed with the predictions of previous numerical simulation of transport of macroplastic exported from Ba Lat River of the Red River[13].”

45. Line 245-264. As it stands, I am unhappy with this section. Not with the section itself. The section itself is valuable, BUT you need to reword your introduction if you want to include these results/dicussion as well.

Response: We have revised our introduction for these results and discussion.

46. Line 277-284. I would take this further. Based on your results where should it be? Or do you need more information, if so,, what information?

Response: This is a suggestion for future study and further information (for example, the density of marine debris accumulation, distance and depth from shore, income and other social-economic status of the coastal communities in that area, etc.) is needed to identify

these plastic cost hotspots. We suggested that the prioritised locations would be the areas with the high marine debris accumulation rate and low-income coastal communities.

Conclusion:

47. Line296 Remove “Plastic pollution of coastal and marine environments is a persistent, pervasive, and costly challenge 296 impacting nearshore fishers and their local communities the most.” I don’t think it's the place or adds value. Combine the rest into one paragraph to remove repetition. And then readdress your conclusion once you have reworked your intro, results and discussion, focusing on your key focus, which is slightly unclear at this stage.

Response: We have revised the conclusion accordingly.

Methods

48. Bear in mind. I have read this section last, and am going into it with preconceived ideas. Now, one of the issues I have till this point is that you did not provide a summary of what you did earlier. But I know your results, so that gives me an idea of your methods. Going in, I think you ran convergent surveys rather than a parallel design. I know already that I need that confirmed. And I know I need you to include the full survey questions for both the quantitative and qualitative in the SI – you do not currently have that. So please include both surveys questions in the SI.

Response: We have revised the Method to make it clear for reader. Full survey questionnaire and raw data is attached.

49. Line 320 – where in the SI.

Response: we have changed ‘(Supplementary Information)’ to ‘(Table S6, Supplementary Information)’.

50. Figure 4 – nice maps, thanks. The time the figure appears, you have not explained what you mean by “main…post-survey.” I presume you mean the first was quantitative and the second qualitative. That’s fine if that’s your wording – but where have you mentioned the two surveys like that? Not yet. You need to mention in text before the figure.

Response: We have updated the terminology throughout the manuscript, renaming the two surveys as the **“socio-economic impact survey”** and the **“verification survey,”** alongside the waste audit surveys.

51. Line 331 so you actually did the waste audits. That wasn’t clear until now – I had assumed you requested the fishers to provide a breakdown. Could you please include this in the summary at the beginning of your results? You really did do an inordinate amount of work for this paper.

Response: We have included the method summary at the beginning of result section, as well as revision of Method section

52. Line 241 – before participation selection, please say how the surveys were developed. Even if you give a summary on the lines of: 2 surveys (quantitative and qualitative OR structured and semi-structured interviews OR first and second– whichever wording you settle on) were developed in an online design (details in SI1). You have some nice information in SI1 but don't refer to it – I would here. Please note that you frequently change the terminology used to refer to the surveys. Please select a consistent term for each, provide a clear explanation, and use it consistently throughout. Please do not settle on the terms "first" and "second" that tells us very little about each survey.

Response:

We revised throughout the manuscript on how we refer to the two surveys period as "The economic cost survey" and "The validation survey". Both surveys are structured interviews, where we meet the fishers in person and interview them for about 45 minutes based on a set of interview questions (available in SI).

These interview questions consisted of both questions to collect quantitative data and qualitative data.

Quantitative data are defined as data that can be measured in numbers; for example, on average how much time do you spent cleaning marine debris from fishing net in each haul; how many people were typically involved in that process; and Likert-scale questions: what is your level of agreement to the following statements (5 options ranging from 1-very disagree to 5-very agree)).

Qualitative data are data defined as non-numerical data, for example: open-ended answers to the questions: when do you encountered the most marine plastic debris, where do you encountered it most frequently, what did you do with it…

We added further details about survey protocols in the SI and refer it in the main manuscript. The full questionnaire form was also attached.

53. What language was this all carried out in? If a language other than English, please include the original language questions in the SI along with the English translations – and make it clear in the SI that a translation occurred and how. Are the English versions direct translations or not. That's all important

Response: All interviews were conducted in Vietnamese. Interview data were collected in Vietnamese, anonymised, coded, directly translated into English, and securely stored in spreadsheets for further analysis. Audio recordings from semi-structured interviews and focus group discussions were transcribed, translated, and coded using NVivo 14 (version 14.23.2 (46)).

We have included the original interview questions (in Vietnamese) and their English translations in the Supplementary Information and deposited them alongside the other raw data in PURE with DOI: 10.17861/47e741f5-3810-4f40-9b03-fba8cf825913

54. Line 384:"from 46" please say from "Gesamp, 2019 46"

Response: we changed "from [46]" to "from GESAMP, 2019[46]"

55. Line 384 - OH THIS IS IMPORTANT. I missed this. On your figure 2 I read it as wet and dry weight. Not wet and dry season. I know you clarified that in the figure caption. But please make that clear on the actual figures ie say "wet season" "dry season" as any scientist working on marine litter will immediately read that as wet vs dry waste. Also, that makes my above comment on how did you get the composition by weight very important – is that during the wet or dry season? Or averaged across all. You need to fix that in the figure as well.

Response: We have revised Figure 2 to explicitly label **Season**: "Wet" and "Dry" on the figure itself, in addition to the figure caption, to avoid ambiguity. We have also clarified in the figure and accompanying text that the waste composition by weight refers to seasonal data (wet and dry seasons),

SI methods

56. Can you include a table of contents for your SI or an opening paragraph highlighting what is in the SI – either will work

Response: We have included the table of content in the revised SI.

57. Please include the survey questions from each survey, with my language comment taken into account

Response: We have included the full survey question form and raw data in the SI.

58. Figure S1 – ok I need a summary of this figure written out and included in your method section – and then refer to this for detail.

Response: We have moved this Figure to the Figure 5 in main manuscript.

59. Missing – did you only do the structured questions with the fishers. I.e., did they only get quantitative questions or were they also open-ended? Actually that's a good point – were they quantitative or open ended? You need that info in the summary in the methods and you really need to provide the survey questions so that I wouldn't need to ask.

Response: We have included the full survey question form and raw data in the SI. We asked both quantitative and open-ended questions. We also revise the method to make it clear.

60. Line 77 – 79. That was important. Thank you. Please mention it was converted somewhere in the main paper as well.

Response: We have mentioned it in the revised manuscript. It now reads "All interviews were conducted in Vietnamese and cost was calculated in local currency before converted to USD. Details on the calculation of cost is available in Supplementary Information 1."

61. Line 82. I thought you followed GESAMP – did you vary in the methodology? Is that why you have provided details here? Or did you give the details here for ease? Please make that clear.

Response: We made clear here, since the GESAMP guideline provide a few option for categorising waste.

62. Line 108 - 110 – thank you, I actually wondered that in the main paper. I would summarise this and add it to the main as well.

Response: We have revised the manuscript accordingly with key information included.

63. Line 112-127 I am not convinced this should be in the main paper. If you leave it here due to space, I would refer to it. You do not currently.

Response: Data limitation was mentioned in the original manuscript before Figure 5 and refer to SI.

2. Reviewer #2 (Remarks to the Author):

Comments on the manuscript by Nguyen et al.:

2.1. This study was conducted in three distinct areas of Viet Nam, where fisherman were interviewed for their perception and experiences regarding plastic waste pollution during their fishing activities. The authors also conducted a waste survey in these regions to determine plastic waste composition. In my opinion, the authors highlight essential aspects related to plastic pollution and its effects on coastal livelihoods by interviewing local fishermen and related authorities, which are often disconnected from traditional field surveys in scientific publications.

Overall, I found this work is relevant to the study field and that authors make a significant contribution, and that the level of detail provided is enough for this work to be reproduced and verified by other researchers.

Response: Thank you for recognising the contribution and relevance of the study.

2.2. I would like the authors to revisit their statement at the end of their introduction where they say they aim to “advance marine plastics research by focusing on three key areas”, and check whether these have been fully addressed in their discussion and conclusion. It really benefits the overall understanding of a manuscript to have such messages being recalled throughout the manuscript and backed up by your own evidence or by relevant literature as appropriate.

Response: We have added in the conclusion and discussion sections details on new findings from this study that helps advance marine plastic research. These key findings are:

- Provide empirical data about the monetary costs and risks from marine plastic debris on fishers.
- Reveal the disproportional economic burdens on communities with lower income and located at outlet of major plastic-polluted rivers, which have significant regional and global implications.

2.3. I found that the claim presented in the conclusions of how plastic unproportionally affects coastal communities like fishermen is very important. However, I believe the authors could make more use of similar previous work to place their findings in a **wider regional or even global context. Is this observed in other parts of the world? Is there any indication in the literature of how strong the income factor is when defining effects of pollution, if compared to other socio-economic or environmental factors?**

Response: We have added a discussion referencing existing literature on environmental injustice, highlighting how the burden of plastic pollution is unevenly distributed among nations and income levels. These references were added to the discussion section about Inequities in the impacts of plastic pollution in the revised manuscript.

2.4. For another claim in their conclusions, the authors indicate that fishermen have a strong understanding of plastic waste pollution in their region and that this knowledge should be better explored in designing solutions. Again I find this an essential and very important finding that was could be more explored and strengthened by looking at and deeply comparing with previously published literature on the same subject.

Response:

Thank you for your suggestion. We have revised the section "Using local knowledge to identify and mitigate plastic pollution hotspots" extensively that discuss about this topic. We have added further comparison with previous published literature in this subject in this section. We would also like to note that the existing literature on this topic is quite limited and hence the goal of our paper is to address this knowledge gap.

Other targeted comments from me are:

2.5. I believe the overall text flow could benefit from eventual reminders that this study **focussed on small scale fisheries**, which is not made fully clear until the reader gets to the discussion of methods. This is specially important if authors believe that small and larger fishery vessels can have significantly different contribution to plastic pollution in their study region.

Response: We have revised the manuscript to more clearly and consistently state that this study focuses on small scale nearshore fishers, including adding explicit references in the Introduction and Results sections to improve clarity for readers. As definitions of small-scale versus large-scale fisheries can vary, we have opted to use the term "nearshore", as defined in the Methods section.

2.6. I suggest the **number of** interviewees corresponding to the data is indicated in each figure.

Response: We have the number of interviewees (n) indicated in Fig.1 and revised to include n in the revised Fig 2.

2.7. I saw from the results on the fisher knowledge that their findings on plastic waste is also a mix of fishery-related and consumer plastic items, as were the findings from the waste audit. I would like to see this distinction more evident throughout the manuscript as to indicate that not all plastic waste affecting fishermen, and maybe not even most of it, comes from fishery activities.

Response: We have revised the manuscript to more clearly distinguish between fishery-related plastics and consumer-derived plastics identified by both fishers'

knowledge (Supplementary Table 5) and the waste audit (Figure 2). This distinction is now consistently highlighted across the Results and Discussion to clarify that a substantial proportion of plastic waste affecting fishers originates from consumer plastic debris, reinforcing the need for solutions that address both fisheries practices and broader land-based waste management systems.

2.8. I found the results indicating a potential intervention being a “fishing for litter” programme very valuable, i.e., “fishers indicated that they would need to be paid a median buyback price of 0.16 (0.08 - 0.23) USD kg-1 of marine plastic debris”. This type of information can be directly used towards mitigation measures, which are often hard to track. **I suggest this finding is highlighted again in your conclusions.**

Response: Thank you for the suggestion. We have revised accordingly in the conclusion.

2.9. It was not clear to me whether the cost burden from fishing vessels in the different study sites **was from perceived or actual cost burden**. To me it would be useful to define this as readers will be more able to compare values with other studies.

Response: The cost was calculated based on actual cost that was calculated based on information reported by the fishers, such as time lost to cleanup marine plastic debris from the catch, labour cost of crew members, etc.
The details of how the cost was calculated was presented in section Cost Estimation (line 354-380 of main manuscript) and SI (line 55-79 of SI).
We added the clarification in the n the Method section of the revised manuscript.

2.10. I found the reports of accidents related to marine plastic very alarming. Are there any reports of accidents in other regions of the world? I believe it is very important to emphasize that these accidents happen and are not isolated cases, and bringing other examples would really help.

Response: Reports of accidents led to crew’s injuries, casualties or missing caused by marine debris was reported in peer-reviewed journals including South Korea (Ref 31. Cited in line 246), USA and other international water (Aretoulaki et al., 2021). We added these two references to the revised manuscript.

Several accidents related to marine debris were briefly mentioned in local news in other location in Vietnam but not included in peer-reviewed paper. We decided not to include these news in the revised manuscript.

We revised the sentence to provide more context on the marine plastic debris related accidents: “Our study also revealed that marine debris results in serious accidents, especially when crew members must untangle fishing nets and ropes from the propeller. Although marine plastic-related accidents are rare and not all

fishers directly witnessed the incidents (making it difficult to verify whether the entangled ropes originated from their own fishing vessels or from ALDFG) the accidents reported emphasise the potential risks to nearshore fishers' health and safety. Reports of accidents led to crew's injuries, casualties or missing caused by marine debris was previously reported in other countries[18,31]."

2.11. In the discussion, the authors say "The direct cost estimated in this study is 0.6 to 3.6 times different from previous estimates for other provinces in Viet Nam (See Supplementary Information T8). The variability in estimates from elsewhere is associated with local demographics and environmental context. In addition, previous studies often only accounted for direct costs (i.e., gear damage, repair cost, time spent sorting catch from plastic) and did not include lost opportunity cost 32, which we do account for (i.e. potential revenue from time lost that could otherwise be spent fishing, reduction in catch due to interference with gear)."

Could the authors give more detail on other studies regarding their environmental and socioeconomical aspects that can differ from the present work? I think the reader needs to have more detail on the main manuscript, rather than having to go to supplementary table 8 to be convinced of this. Similarly, could the authors please elaborate a bit more on why the inclusion of this factor onto cost calculation is important? I hope the authors find these comments helpful. My overall aim was to instigate critical thinking in order to better place this work in a wider context and enhance visibility and relevance once it gets published

Response: We thank the reviewer for this constructive and helpful comment. We agree that clearer contextualisation in the main text strengthens the manuscript and reduces reliance on SI.
In the revised manuscript, we have added further details and context of related studies for a fair comparison. A concise comparative synthesis has now been added to the Discussion (lines 229-242 and 292-313 in the revised manuscript) to highlight why these contextual factors influence observed patterns of plastic pollution and responses, while Supplementary Table 8 is retained for detailed reference. We have also clarified why inclusion of these factors is critical for cost calculations, as omitting them can underestimate true costs and misrepresent feasibility, particularly in small-scale and coastal fisheries. These revisions better situate our work within the wider literature and enhance its relevance for policy and practice.

3. ## Reviewer #3 (Remarks to the Author):

3.1. The manuscript, entitled “Marine plastic pollution undermines the livelihoods and income of fishing communities in Vietnam,” focuses on quantifying macro plastic pollution along the coast from the perspective of the affected fishing communities in three major fishing areas in Vietnam (Nam Dinh, Ben Tre, and Hai Phong).

The topic is fair. However, based on this consideration, the manuscript contains some findings that may contribute to the knowledge in the field. However, the manuscript quality is average; it contains some unclear points that the authors must address. The results and discussion section requires improvement, particularly in terms of a strong or deep interpretation of the results. Although the manuscript includes adequate sample sizes and distinct sampling groups, the authors did not conduct the analyses for these groups. This omission limits the depth and robustness of the findings, as subgroup-specific insights could have provided a clearer understanding of differences among the fishers. Some examples are below.

Response:

Thank you for this thoughtful and constructive comment. We acknowledge that while the manuscript presents relevant findings and adequate sample sizes, the interpretation and analytical depth required further strengthening.

In response, we have substantially revised the Results and Discussion sections to improve clarity, analytical rigor, and depth of interpretation. Specifically:

- We have expanded the data analyses to include subgroup-specific analyses (e.g. fishing gear type, study location, and season). Beside quantitative analyses with statistical tests, we have also corroborated the data with descriptive and qualitative comparison to strengthening the discussion, especially where subgroup comparison were not statistically justified due to sample size and distributional constraints. For example, the fishers participated in the survey was disproportionately composed of trawl fishers (n=167) due to the limited presence of gillnet fishers (n=32) at the surveyed port. This also represent the actual distribution of nearshore fishing gear used in the study locations. We have also include the Data Limitation in Supplementary Method in the SI.
- To enhance robustness and transparency, additional statistical results supporting subgroup analyses have been included in the Supplementary Information, and raw data are now included with the manuscript.

3.2. Update figures (legends) for clarity and add essential figures or tables into the context for robustness checks on the results (for example: Figure 2) The data descriptions are sometimes unclear, or the supplementary data is presented in a confusing way that makes it hard to understand.

Response: We have substantially revised the legends of Figures to clarify the data. In the legends, we have mentioned the datasets used (gear type, sample size,

study locations, and seasons). The descriptions of the box and whisker plots (including mean, medians, interquartile ranges, whisker definitions) were included according to the journal format. The full statistical test and breakdown data were also included in Supplementary information.
The data for Figures 1 and 2 are included in Supplementary Table 1 and 2 in the SI, respectively. We have also included the raw data and data for figures in the attached Supplementary Information.

3.3. The data presentation also needs revision. Please check data line 94

Response: Line 94: "45% of interviewees (n=90) reported having 4 (1 – 9) incidents per year of propeller entanglement": This sentence means that there were 90 fishers (out of a total of 199 interviewed fisher) that reported they experienced propeller entanglement with the median frequency of 4 times per year (Q1-Q3 =1-9 times per year). We have revised the sentence to avoid confusion for reader, the sentence now read: "*Among the 199 fishers interviewed, 45% (n = 90 fishers) reported propeller entanglement with discarded or lost ropes and nets, with a median of 4 (1–9) incidents per year.*"

3.4. Please clarify lines 143-156:

Response: Line143-156 is the caption for Figure 2. The description and explanation of data, box and whisker plot, and statistical test are provided according to guidance of Nature Portfolio reporting. Further clarification was added in Methods and Supplementary Table 1)

3.5. Please clarify lines 164-179: there are some mysterious numbers (didn't see from supplementary data T7).

Response:

- We have revise to include "(See Supplementary Information T7) in the correct place in main manuscript. We have changed all reference of "T7" to "Supplementary Table 7", to align with the journal's format.
- Lines 164–179 of the original manuscript summarise the perceptions of 199 interviewed fishers regarding the current marine pollution situation and their willingness to participate in the incentivised "Fishing for Litter" programme. Some data were previously presented only in the main manuscript and not in Supplementary Table 7 to avoid duplication. However, following the reviewer's comment, we have revised Supplementary Table 7 to include all data reported in the main text. Specifically:

+ Current perception and practice of dealing with marine plastic: "*57% of fishers felt unable to take action to reduce the impact of marine plastics. The majority (77%) continued to discard marine debris caught in their nets back into the ocean, with 18% only retaining valuable*": We added these data to the last two rows of Supplementary Table 7 in the Revised SI.

+ *"The majority of fishers were interested in participating in a "fishing for litter" programme (n=196: 56% strongly agree and 23% agree)*": The frequency of fishers who Strongly Agree and Agree with the statement was updated to the Supplementary Table 7 (4th row from the bottom now read: *"(Frequency of choice 5 (Very agree) = 56%*
Frequency of choice 4 (Agree) = 23%)" to match the data presented in the main manuscript.

+ *"Fishers indicated that they would need to be paid a median buyback price of 0.16 (0.08 - 0.23),… fishers in Hai Phong requested a higher marine litter buy-back price ($H_{1, 119}$=19.9, p<0.001)*": We added the Interquartile Range of the median buy back price (0.16 (0.08 - 0.23) USD kg^{-1}) and the statistical value of Kruskal-Wallis test in the third row from the bottom of the Supplementary Table 7: it now read: "*Median = 0.16*
(IQR 0.08 – 0.23) ; Kruskal-Wallis test, H (df=1, n=119) = 19.9)"
The non-significant statistical test between gillnet and trawl fishers with $H_{1, 119}$=0.34, p=0.6 was only mentioned in the text of the main manuscript and not in this table.

3.6. Unused data: Table S3. It is for this reason that I recommend this manuscript for a major revision.

Response: Table S3 (Supplementary Table 3) is demographic data of participating fishers in the socio-economic impact survey. We believe that these data provide a further context for readers about the demographics of fishers in the study areas. However, these data are considered supplementary results and suitable in SI.

We added a summary sentence of the Supplementary Table 3 in the main manuscript: *"These regional differences are linked to a number of demographic factors (Supplementary Table 3 and 6)."*

We added a cross citation to the Supplementary Table 3 of the revised manuscript, section Methods: *"The impact assessment survey consisted of 199 structured interviews with nearshore fishers (see demographics in Supplementary Table 3) to quantify the economic impacts of marine plastic debris on their livelihoods"*

RESPONSES TO REVIEWERS' COMMENTS

Reviewer #1 (Remarks to the Author):

This report is a second round review by Reviewer 1. The reviewer was also tasked with considering whether Reviewer 2's comments were adequately addressed. Thus, this review considers both reviewer comments.
Reviewer 1 comments
Please find attached a revised copy of the paper with minor grammatical corrections – these were things I noticed while reading it. It was easier to make the change directly in the track than to write it out for such small changes. Please note these are minor changes.
Please note you also switch between when to use the "-", e.g. mixed-method or mixed methods. You need to check this and other such consistencies throughout the paper.

Response: Thank you for your revision and comments. We have revised the manuscript to consistently use the term "mixed method" throughout, and have made necessary grammatical corrections.

Abstract:
Line 21/22, please put in the type of interview.
• i.e., your response in green below the names of the survey – this should be in the abstract as well. Keep the words "structured and post-hoc", but add "the Economic cost survey" and the "validation survey" wording.

Response: Thank you for your comment. We have edited accordingly in the abstract.

• I also think it is important to include that both surveys are quantitative and qualitative.
"We revised throughout the manuscript on how we refer to the two surveys period as "The economic cost survey" and "The validation survey". Both surveys are structured interviews, where we meet the fishers in person and interview them for about 45 minutes based on a set of interview questions (available in SI).
These interview questions consisted of both questions to collect quantitative data and qualitative data.
Quantitative data are defined as data that can be measured in numbers; for example, on average how much time do you spent cleaning marine debris from fishing net in each haul; how many people were typically involved in that process; and Likert-scale questions: what is your level of agreement to the following statements (5 options ranging from 1-very disagree to 5-very agree)). "

Response: Thank you for your comment, we have edited accordingly to mention that both surveys are both quantitative and qualitative in the Method section. We cannot mention this in the abstract due to 150 words limit of the abstract.

Highlights
Point 2"- Consequences of marine plastic litter cost each fishing vessel >10% of the annual revenue and 25% of annual income for vessel owners."
• My understanding from the abstract is that this is annual revenue for individual fishers. From here and the paper, that it's >10 for annual revenue per boat for the fishers. Do you mean each fisher's individual income or the annual income of the vessel? Can you just make sure this is clear here and throughout the paper? Currently is appears at odds with the abstract. Please check the wording and clarify in text where relevant. I think the issue is really in the abstract wording.

Response: Thank you for your comment. We have clarified the wording throughout the manuscript, particularly in the Abstract, to ensure consistency.

Specifically, the estimated costs represent approximately 10% of the total annual revenue generated per fishing vessel, and around 25% of the vessel owner's annual income (i.e. net profit after expenses).

To align with the journal format, we have also removed the Highlights section in the final manuscript.

• Additionally, this isn't quite right. Neither here nor in the abstract, your results show a 7-18% range between the different sites. I would put either the range, the 12% or both into the abstract and highlights rather than 10%. I prefer both. But I do not like the use of ~ given that you have 12% and a range. I would apply the same change to the annual income for vessel owners. Apologies for not identifying this in round 1. Essentially, I am asking you to change the abstract and highlights to what you have in the main paper, i.e., "This amount is equivalent to 12 (7 – 18) % of their annual revenue or 25 (15 - 48) % of the fishing vessel owner's income"

Response: Thank you for your comment. We agree and change to "equivalent to 12% of the annual vessel revenue and 25% of the owner's income" in the abstract and throughout the manuscript.

Results
It is still important to clarify whether your weight is dry or wet weight. Please clarify in the main text. I know you have it in the SI.
A note for your own future research – we really do need the clean and dry weight values. I know it's an added logistical and personnel issue, but we do need it. I think, in this case, because it also affects a fisherman's ability to haul and land litter, your use of wet weight is arguably acceptable. But for comparisons with other marine litter data, the clean and dry is becoming more important.

Response: Thank you for this valuable comment. We have clarified in the main text that marine debris mass was measured on a wet basis after draining excess water, following standard marine debris audit protocols. A detailed description of the method is provided in the Supplementary Methods section.

While dry mass measurements offer greater accuracy under laboratory conditions, they were not feasible in our field measurement setting. Moreover, wet mass

provides practical relevance for waste management operations and incentive-based marine plastic debris collection programmes. Therefore, the use of wet mass is standard in waste audit of marine debris.

We have now explicitly noted this in the Methods section. In addition, we have revised the manuscript and figures to consistently use the term “mass” instead of “weight” to align with journal reporting standards.

Methods
I note that you do not include the reference to the Likert Scale for the quantitative scale in your methods. Reference to it needs to be in the main paper.

Under data collection:
It is important to clarify that both surveys used a mixture of quantitative and qualitative questions, including Likert-scale and open-ended questions ect. Please include.

Response: Thank you for this comment. We have added reference to the Likert scale and this sentence in the Method section of the main manuscript: “In both surveys, we used a combination of quantitative and qualitative interview questions, including Likert-scale measures of agreement to and open-ended questions.”. The full interview question forms were also included in the uploaded data repository as well as in the Supplementary Information.

We have also added the following detail in the Method section to further clarify : “In both surveys, we used a combination of quantitative and qualitative interview questions, including Likert-scale measures of agreement to and open-ended questions. When designing the survey, we acknowledged that Likert scale questions primarily capture direction (positive or negative) and to a lesser degree intensity (level of agreement or disagreement). While binary answer questions (“yes” and “no”) were considered, we wanted to capture the intensity of beliefs especially in relation to perceptions about decreasing fisheries resources, increasing risks to crew, longevity of plastic waste, and motivations to bring plastics back to shore [53]. The 5-point Likert scale was chosen over the 7-point alternative due to its ease of administration and reduced completion time.”

Under waste audit: “A waste audit was conducted on board fishing vessels by observers following the method adapted from GESAMP, 201954.”
What adaptations did you make? This is easily answered by adding a reference to SI 1.3

Response: Thank you for this comment. The adaptation relates to the categorisation of marine debris. As the GESAMP guidelines provide multiple classification approaches, we adopted the UNEP–IOC scheme for this study. We have clarified this choice and added the relevant reference in Supplementary Information 1 (Section: Waste audit methods).

SI
SI 1.1.
Again, please include a sentence on the Likert scale used in qualitative research. Currently, you only have it in the table lower down.
Please clarify how many researchers conducted your interviews. If more than one, mention training/discussion to prevent influencing the interview through different conductors etc.
Your questions are not in the SI – please add them. I see they are with the raw data, but they need to be in the SI. I am presuming this is because the SI is currently in a word doc and you will combine the documents when they are in PDF form -but your contents table in the word document does not make this explicit. Thank you for including both languages.

Response: Thank you for this comment. We have added sentences and interview forms in the Supplementary Information accordingly.

Specifically, we have added: "In all structured interviews, we used a combination of quantitative and qualitative interview questions, including Likert-scale measures of agreement (1=strongly disagree to 5=strongly agree) and open-ended questions" and "All interviews were conducted by four researchers using a standardised interview protocol. Prior to data collection, all researchers jointly test and revise the interview form to refine question wording and align the interview process (Figure 5 in the main manuscript). This training ensured consistency in delivery and minimised interviewer-induced bias across surveys."

The interview forms were added to the end of the pdf of Supplementary Information.

Comments on reading the rebuttal:
Thank you for adding uncertainty ranges.
Thank you for expanding the SI.
Thank you for adding the raw dataset as well – it is always good to have that publicly available.
I respect your decision not to include the grouped qualitative results table in the main paper. Despite my request. I can see why you have made this choice. However, I am noting that you are underutilising this data set in this paper. I work mostly quantitatively with some qualitative work, and I can tell you that if I were my qualitative colleagues, they would not allow this through review. However, as you have so much data, and have used key quotes, I respect your decision. Thanks for adding a partial thematic table to the SI.
I appreciate the difficulty of balancing such a wide range of datasets and results between the main and SI, and though you disagreed with some of my recommendations in round 1. I think the changes you have made are good.
"This paper focuses on fishers' perspectives and knowledge of marine plastic pollution impacts, rather than technical comparisons of waste audit data, which will be addressed in a separated forthcoming paper." – I look forward to seeing that paper, I will look out for it.

While injuries and fatalities related to marine plastic pollution do occur in practice, such incidents were not widely or frequently reported by fishers in our study (total 3

out of 199 fishers interviewed). – this is still a high number. Just a comment too your comment. No change required.

Response: Thank you for your thoughtful and constructive comments. We appreciate your view on the qualitative dataset and agree that it represents a valuable component of the study. Our intention was to maintain a clear narrative focus on fishers' perspectives and socioeconomic impacts within the constraints of the main text, while providing additional qualitative detail in the Supplementary Information. We acknowledge the potential for further in-depth qualitative analysis and will consider this in future publication. We also appreciate your recognition of the revisions made and your understanding of the balance between datasets. Thank you for your encouraging feedback.

Reviewer 2 comments

I agree that the authors have strengthened the paper in response to Reviewer 2's points. I am only commenting on the points which may not have arguably been 100% covered by the authors.
2.4 – I agree with the authors that the literature is limited to support this request, and believe that they have covered it adequately, given what is available and the gap they are addressing.
2.9 – To be honest, I could not see where in the method section this had been clarified. However, I think it is clear now, throughout the manuscript, and in other changes that this is a calculated cost. Thus, I believe this comment is adequately covered.
2.10 – I believe the reported references are adequate, and agree that you do not need to add the news article, as you have the journal references. However, as the authors identified, the references cover other nations, not Viet Nam. Given that, I probably would have included the news reference out of interest. However, I believe the authors have adequately addressed the point.
2.11 – The authors have expanded on this. The initial reviewer may potentially have been thinking of a core comprehensive expansion; however, I believe the succinct expansion provided is good.

Response: Thank you for your responses to the revisions that addressed Reviewer 2's comments.

Reviewer #3 (Remarks to the Author):

I am satisfied with the authors' responses to the questions and issues raised in my initial review.
The revised manuscript is clearer and easier to follow after incorporating the reviewer's comments.

Some minor comments
1. The minor issue relates to grammatical check.

Response: Thank you for your revision. We've gone through the manuscript again to check for grammar and typo issues.

2. Why fishers may overestimate debris quantities? (Line166)
>> Please provide the further explain

Response: Thank you for your questions. We have provided further clarification and additional analysis in the revised manuscript, as outlined below.

"This overestimation likely arises because fishers tend to base their estimates on occasions when debris is present in their nets, while overlooking periods with little or no plastic. When waste audit observations below 0.5 kg plastic per haul were excluded, fishers' estimates closely matched field measurements, with no significant difference between estimated (2 kg $haul^{-1}$) and observed values (2.4 kg $haul^{-1}$; Kruskal–Wallis test, $H_{1,351} = 0.18$, $P = 0.7$). We also acknowledge that the limited spatial and temporal coverage of the sampling campaign may not fully capture the variability of real fishing conditions, which could contribute to the observed differences. Fishers demonstrated their local ecological knowledge (LEK) on marine plastic debris by reliably recalling the most common marine debris items and their increased abundance during the wet season, including the seasonal fluctuation in Ben Tre province (Figure 2 and Supplementary Table 5). Together, these results indicate that fishers' local ecological knowledge can provide a reliable and cost-effective complement to field-based sampling for estimating and monitoring marine plastic debris."

3. Clarification of whether sensitivity analysis was conducted to evaluate uncertainty of the cost estimation analysis?

Response: We thank the reviewer for this comment. A full sensitivity analysis was not conducted, as it falls outside the scope of this study, which focuses on empirically quantifying observed socioeconomic impacts reported by fishers rather than developing a model-based economic assessment.

Uncertainty is nevertheless inherent in the cost estimates, reflecting variability in fisher-reported experiences, operational conditions, fishing locations, and the heterogeneous nature of plastic-related impacts.

To assess robustness of our estimation, we conducted a rapid one-way sensitivity analysis on key input variables. This indicates that total time lost due to marine plastic interactions is the primary driver of cost estimates, while variation in annual revenue of fishing vessels and fishing effort has comparatively smaller effects.

These findings suggest that productivity loss from operational disruption is the dominant mechanism underpinning economic impacts, and that the overall conclusions are robust to reasonable variation in key assumptions. Given the scope of the manuscript, these sensitivity analysis results are not included in the final version.

We also added this clarification at the end of our Supplementary Method for cost calculation in the Supplementary Information.

4. Figure captions are too detailed but could be shortened for readability.

Response: Thank you for your comment. We have revised and adapted our figure caption to the journal requirement.

Overall

The manuscript titled" Marine plastic pollution undermines the livelihoods and income of fishing communities in Vietnam" reports on the impact of plastic pollution on fisheries near two main rivers in Vietnam. The research focuses on the manatery cost, but also considers other impacts. The location utilises fishing communities that operate close to the estuaries of two very polluted rivers, the Mekong and Red Rivers. My understanding of the amount of work covered within this paper continued to grow as I read the paper. In addition to the above-named focus, the paper also includes waste audits on marine litter collected by fishers from the sea, and understanding from fishers on temporal and special hotspots.

I would like to highlight the importance of the work undertaken by the authors. They have focused on two primary gaps. 1) They have captured the socioeconomic impact of marine plastic pollution, with a quantifiable monetary value. I have been looking for such references for years, and would like to commend the authors for tackling this gap. Additionally, research within the global South and on the impact of plastic pollution is also an important area of work. Their supplementary work on litter collected by fishers at sea and fishers' understanding of hotspots is also interesting and fills similar gaps.

Having tried to find references on the measured cost of marine litter (further than cleanup costs), I consider this research to cover a very important gap, with interest from a wide range of readers and stakeholders. And I commend the Authors in their work on this. However, some additional work on the manuscript itself is required, particularly due to the layout and general readership of the chosen Journal. I need to be able to read and understand the paper before even getting to the methods. The abstract/intro, etc., is well-written, but it does not fully capture what has been done – I discovered new areas of inquiry in the results, such as the audits.

I think the presentation of the results needs to be revisited, e.g Figure 1. The written focus does not quite align with the figures. E.g. Figure 1d is the main result, but then the text focuses on other costs or the breakdown of costs rather than the differences between the fishing types (shown in Figure 1a-c). So the focus splits. The authors should consider revisiting the paper in light of their key results and focus. Additionally, the qualitative results (quotes) are highlighted as valuable but are not fully visible for full utilisation of the current form – I have given detailed comments on this within the results and discussion section.

Overall, the paper could benefit from restructuring to follow the focus of the paper. I have placed a detailed comment on this within the introduction section and then throughout.

I want to emphasise that the amount of work invested in this manuscript is truly phenomenal. Not just the use of a mixed-method survey approach, but also the additional waste audit. I would really like to see this work published. Hence, I encourage the authors to approach the comments in the supportive manner they are intended.

Overall focus

I give further details below. Please note that my approach is to write my detailed comments in order of my thoughts, like a flow of consciousness. This means that I might raise a point earlier that you deal with later. If this happens, I think you should consider moving the latter section earlier or mentioning it briefly and indicating that it is dealt with in detail later.

Detailed comments:

Title

I like the title – it's to the point and includes all the necessary information.

Abstract

The abstract is well written. It captures the key points of the paper.

I appreciated both the monetary value and how you then put it into the framework of the overall revenue and income.

Honestly, one of the best abstracts I have read in a while.

Small changes:

- You speak of 2 rivers and 3 study regions. From the abstract alone, it's unclear whether you conducted two study regions near one river or one study region by each river, along with a control further away.
- Line 20: "We found that the total incurred costs" Please change to "we calculated that…" I initially read it that way, as the fishers were telling you that cost, rather than what you calculated.

Returning to the abstract and highlights after reading the paper,

I still really like your abstract and highlights. BUT, and this is important, you make no mention of the waste audits in either. You are unlikely to have the words available for the abstract, so please work the waste audit work into your highlights. You may be able to include the waste audit in the second line of your abstract – have a look.

Keywords

Please check the keyword requirements of the paper.

1) I think you have too many
2) I use the rule of not using a keyword if that word is in my title

Highlights

Not as strong as your abstract – but fine. I would revisit once you have restructured the paper to really highlight your key takeaways.

Introduction

Overall, your introduction is succinct. However, it could improve with slightly more information – especially given the general audience of the Journal you have chosen.

I recommend bringing in a few lines on the socioeconomic impacts we do know on marine litter (globally and regionally, before you consider locally). You touched on the local impacts in theory slightly in line 61 as a gap. However, I would also include other socioeconomic aspects that we know or expect to be impacted by marine litter, and then highlight the

significant monetary gap affecting local fishers. I know that much of the socioeconomic impacts of marine litter have been more theoretically conceptualised for years rather than measured outcomes (yours is a measured outcome), and I really think you can highlight that gap that you fill within the greater socioeconomic space.

Line 48 -50 – I know and appreciate the local issues that drive plastic pollution rates in Asian countries. However, you need to include the global issues surrounding the plastic waste trade in the region, which is driven mainly by Western countries. It's an important contribution, and needs to be added here.

Line 56 – I found it interesting that you used Vietnam in the title and Viet Nam in the main text. And to be fully honest, I read into the difference and I learnt something new here. I actually appreciate the point you have made by using Viet Nam. And can see why you still utilised the English form in the title. It could be argued that you should either use Vietnam or Viet Nam throughout. Personally, I respect the choice you have made – and I presume a Vietnamese author led the decision.

Lune 64 – please remove "(See Supplementary Information F1 and methods)" – make sure it's in the methods, but do not have it in the introduction.

Line 69 – 71. Your introduction perfectly supports your point 1. Your points 2 and 3, however, are not. You need to make adjustments for this. However, I will come back to this after reading the whole paper.

Comment after reading the full paper:

I am not happy with your introduction after reading the whole paper. And I think this comes from the fact that you have collected so much information (a tremendous amount), but then in the different sections (abstract/intro/results/discussion), you have focused on different sections of information. Even between your figures and written text, there is a disparity. You need to decide what your primary and secondary results are. I would like to see you report it all; however, I need you to restructure each section carefully to show the move from primary to secondary results. Or to word it another way, list the order of your results in order of priority (most important at the top) and then restructure your entire paper to reflect that. Then, return to your introduction and include the necessary background information for each piece of information you have collected.

Line 69 -71 "(2) limitations of macroscopic models that rely on assumptions and lack empirical data from the impacted communities, and (3) potential of local perceptions to enhance such models, a factor that is often overlooked." I would roll this under one point.

Results

You have some truly fascinating results. But you need to work on some recurring issues:

1) There is a misalignment between your focus on your figures and the focus on the text. This needs to be addressed.
2) Much of your main results figures/tables are in the SI. This is an issue.
3) Although you have included standalone quotes within the results, this approach somewhat diminishes the impact of your qualitative findings. I would recommend including them all (in text and in SI in a table divided into themes). For this, I recommend looking up the latest socioeconomic work on microplastics – specifically,

papers that have utilised both quantitative and qualitative methods, which provide examples of such tables.

4) You haven't fully captured the requirements of the layout of the Journal here. I would include a brief summary of your approach here at the start of the results (1-2lines) before going into the results. I see examples from the latest articles vary in this approach. But I recommend it, e.g https://www.nature.com/articles/s43247-025-02670-x

   Remember, the layout of this Journal requires me to be able to read the results without a detailed understanding of the methods (I only read them later). Thus, I need to know the differences between the sites, and what your values mean.

Line 76 (See Methods and Supplementary Information for methodological details). In line with the above comment, I would remove this.

The reason this becomes an important point is that I initially thought your first results paragraph was discussing the amount of money the fishers claimed it was costing them. But I can see from the SI that this is not the case. Instead, it is the total cost you calculated – that's important, and it needs to be clear here.

You have a good blend of quantitative and qualitative, and have incorporated the quotes nicely. However, as you have utilised the qualitative, you could take this further. I have written elsewhere how. Further to my remarks on this point, please consider if you can take the qualitative results still further.

"Error!source not found" in the PDF but not the Word version – so an error in journal processing. Just be aware.

Lie83- 92 – need to name this figure/graph where these numbers come from. I think the detail here is important. Additionally, it appears that these values are not derived from figures in the paper. They are the main results, and so the figure/table should be in the main paper.

Line 95 – So here you report these results to be in the supplementary table- as the results are in the results section, I would argue these results should also be in a figure/table in the main paper. Results from supplementary graphs/tables should not really be focused on in the results, but should be used if mentioned only as supporting information in the discussion.

Line 99 – 104. Given this is a socioeconomic study utilising both quantitative and qualitative results, please consider creating a table of quotes supporting different themes. Then use quotes such as this in the text within the discussion.

Line 99-104. These are very good, expressive quotes. They do truly capture the human cost and tragedies involved. This could not have been easy work to explore, capture and report. I would like to express my respect for the resilience of both the fishers and researchers involved with this work. Also, again, showing the human cost here is a key result which is often only expressed in theory within the scientific literature. I think you can take the qualitative work further. It's a key part of this work.

Line 99-120 – I think it would be good to revisit your highlights to include some of the qualitative results captured here. You have it in the abstract, but I would highlight as well.

Line 105- 111. This is an interesting result in line with your quantitative work.

Figure 1. The figure is a good figure. However, the written focus does not quite align with the figure. E.g. Figure 1c/d is a main result, but then the text focuses on other costs or the breakdown of costs rather than differences between the fishing types (shown in Figure 1a-b).

So the focus splits. The authors should consider revisiting the paper in light of their key results and focus.

Line 120 – "Plastic debris composition based on fisher knowledge and waste audits" I got very excited when I saw this section, but I didn't know from your abstract/highlights nor intro that you did this. It is a problem that this came as a surprise.

Line 123 – I know you have limitations on figures within the Journal. But I really do not like that you refer to the supplementary results for what you present as key numbers. You need to revisit this.

Figure 2: This is a good figure. But again, you have data in here not mentioned in the text, which is interesting, ie the gill net vs trawl. Also, Figure2c - is it dry or wet? I presume dry. (*Theres a link here with a comment in the methods – please see that comment*)

Line165 – 171: These numbers do not come from SIT7 – where do they come from? I would like to see the relevant quotes in T7 incorporated into the main paper. Also, do you have more than one quote per point? It would be helpful to include.

Line 172: I would like to see you take the quote usage further – include a table in the main paper showing the key quotes under the different themes. I.e., take all the standalone quotes and the quotes in the SI (and any others you have, so that more than one supports each point), and combine them into one table under the different points/concepts/themes.

Line 174 – interesting but again…I don't like that the table is in the SI. You need to consider a full overhaul of your figures/tables between the main paper and SI.

Line 181: Both the initial and follow-up surveys – presuming the first was a quantitative with the follow-up qualitative – so a convergent design. Not clear at this point. Additionally, this paragraph should be supported with numbers and quotes.

Discussion

Line 190 "Our findings demonstrate the substantial negative impact of marine plastic on the income, 190 livelihoods and safety of nearshore trawl and gillnet fishers." I agree. But I also think this under-sells your results. Maybe I am still reeling from your quotes regarding the injuries and fatalities. Honestly, I think you can make this sentence stronger. And I would. "Safety" is a very safe word to use. Also, I would bring in a summary line on the site difference here – that is significant.

Line 191 "the cost": start a new paragraph.

Line 193: refer to figure

Line 203: You have discussed regional differences first. But your opening discussion line 190 does not highlight this as the most key result. I have in fact asked you to include a summary of the regional difference within your first lines before suggesting breaking into a second pargarph. This highlights to me that you agree – the regional differences are interesting but are not the key results. I recommend you reshuffle your results with this in mind. You have two potential choices: either incorporate regional differences into each discussion part or move this section down.

Line 217: So I see the economic costs you have defined, and this result is your key result.

Line 219: "often did not witness the incidents directly" interesting. But some did right? These are not urban myths. I read this as a few have directly witnessed these events. But be Careful of your wording here, as you cast doubt on that understanding. Be exact in what you mean.

Line 232 Table T8. Thanks for this summary table. It is useful. And I agree with its placement in the SI.

Line 229 – This is an ephamism. I laughed at how carefully you worded that, especially given the size of the gaps in the field. Not a comment to make a change. Feel free to make the sentence stronger if you'd like to emphasise the importance of your work.

Line 235; "In addition, previous studies often only accounted for direct costs…and did not include lost opportunity cost[32], which we do account for", I think this is a really important part of your paper. I think you should emphasise this point more in the introduction. Also, I would actually move this point up (to line 229), opening this whole section with this point. It gets lost here. But it's important.

Line 243-244: I agree. But I would like you to take your qualitative work further. In addition to the table of quotes I have suggested for your results section, I would then move all the standalone quotes you currently have in the results and weave them into the relevant discussion sections. You have done this slightly with line 172. But I would like to see it done with the others as well. I.e., I believe you can include all the quotes (without exceeding the word count, as they will be presented in a table) and then use the key ones you identified within the results to support your discussion itself.

Line246-251 are important, and I like how you have written it. But I would argue this is an aside, and not a main focus of your results…. the socioeconomics is a main result and a key result. The regional differences are interesting and important…and then you bring in the waste Audit work – which I love, BUT I wasn't expecting from your abstract/highlight/intro. If you fix this in your intro, that should solve the issue of line 246-251 not truly being in focus.

Line 249 – LEK? I think the full is only in the SI? Please double-check and fix if needed.

Line 254 – again…you have such a wealth of knowledge in here – I didn't expect this result to creep in as something you looked at. I would greatly appreciate it if you could write a concise summary of your methods at the beginning of your results. This wouldn't be needed in a journal that places the methods at the end. But it is needed here.

Line 257 "fishers' knowledge of plastic distribution reflects reality". I would argue it's the other way round – that the predictions reflect reality because they are supported by the fisher's knowledge. In fact, I feel strongly about that. Please change.

Line 245-264. As it stands, I am unhappy with this section. Not with the section itself. The section itself is valuable, BUT you need to reword your introduction if you want to include these results/dicussion as well.

Line 277-284. I would take this further. Based on your results where should it be? Or do you need more information, if so,, what information?

<u>Conclusion:</u>

Line296 Remove "Plastic pollution of coastal and marine environments is a persistent, pervasive, and costly challenge 296 impacting nearshore fishers and their local communities the most." I don't think it's the place or adds value.

Combine the rest into one paragraph to remove repetition. And then readdress your conclusion once you have reworked your intro, results and discussion, focusing on your key focus, which is slightly unclear at this stage.

Methods

Bear in mind. I have read this section last, and am going into it with preconceived ideas. Now, one of the issues I have till this point is that you did not provide a summary of what you did earlier. But I know your results, so that gives me an idea of your methods. Going in, I think you ran convergent surveys rather than a parallel design. I know already that I need that confirmed. And I know I need you to include the full survey questions for both the quantitative and qualitative in the SI – you do not currently have that. So please include both surveys questions in the SI.

Line 320 – where in the SI.

Figure 4 – nice maps, thanks.

Figure 4 by the time the figure appears, you have not explained what you mean by "*main survey …post-survey.*" I presume you mean the first was quantitative and the second qualitative. That's fine if that's your wording – but where have you mentioned the two surveys like that? Not yet. You need to mention in text before the figure.

Line 331 so you actually did the waste audits. That wasn't clear until now – I had assumed you requested the fishers to provide a breakdown. Could you please include this in the summary at the beginning of your results? You really did do an inordinate amount of work for this paper.

Line 241 – before participation selection, please say how the surveys were developed. Even if you give a summary on the lines of: 2 surveys (quantitative and qualitative OR structured and semi-structured interviews OR first and second– whichever wording you settle on) were developed in an online design (details in SI1). You have some nice information in SI1 but don't refer to it – I would here. Please note that you frequently change the terminology used to refer to the surveys. Please select a consistent term for each, provide a clear explanation, and use it consistently throughout. Please do not settle on the terms "first" and "second" that tells us very little about each survey.

What language was this all carried out in? If a language other than English, please include the original language questions in the SI along with the English translations – and make it clear in the SI that a translation occurred and how. Are the English versions direct translations or not. That's all important

Line 384:"from 46" please say from "Gesamp, 2019 [46]"

Line 384 - OH THIS IS IMPORTANT. I missed this. On your figure 2 I read it as wet and dry weight. Not wet and dry season. I know you clarified that in the figure caption. But please make that clear on the actual figures ie say "wet season" "dry season" as any scientist working on marine litter will immediately read that as wet vs dry waste. Also, that makes my above comment on how did you get the composition by weight very important – is that during the wet or dry season? Or averaged across all. You need to fix that in the figure as well.

SI methods

Can you include a table of contents for your SI or an opening paragraph highlighting what is in the SI – either will work

Please include the survey questions from each survey, with my language comment taken into account

Figure S1 – ok I need a summary of this figure written out and included in your method section – and then refer to this for detail.

Missing – did you only do the structured questions with the fishers. I.e., did they only get quantitative questions or were they also open-ended? Actually that's a good point – were they quantitative or open ended? You need that info in the summary in the methods and you really need to provide the survey questions so that I wouldn't need to ask.

Line 77 – 79. That was important. Thank you. Please mention it was converted somewhere in the main paper as well.

Line 82. I thought you followed GESAMP – did you vary in the methodology? Is that why you have provided details here? Or did you give the details here for ease? Please make that clear.

Line 108 - 110 – thank you, I actually wondered that in the main paper. I would summarise this and add it to the main as well.

Line 112-127 I am not convinced this should be in the main paper. If you leave it here due to space, I would refer to it. You do not currently.

Marine plastic pollution undermines the livelihoods and income of fishing communities in Vietnam

Duc Nguyen[1], Phan Phuong Thanh[2], Heidi L. Burdett[3,4], Inna Yaneva-Toraman[1], Zhiling Liao[1], Qingping Zou[1], Trinh Quang Tu[2], Le Trung Dung[2], Le Thi Thu Huong[2], Ryan Pereira[1], Thomas Wagner[1], Michel J. Kaiser[1*]

Affiliations:

1 The Lyell Centre, Heriot-Watt University, Riccarton, Edinburgh, United Kingdom

2 Viet Nam Institute of Fisheries Economics and Planning, 10 Nguyen Cong Hoan Street, Ba Dinh District, Hanoi, Viet Nam

3 Umeå Marine Sciences Centre, Umeå University, Norrbyn, Sweden

4 Department of Ecology, Environment and Geoscience, Umeå University, Umeå, Sweden

*Corresponding author: m.kaiser@hw.ac.uk

ABSTRACT

Globally, small-scale fishing communities are impacted by increasing quantities of plastic debris accumulating in coastal waters. We studied the socioeconomic impact of marine plastic pollution on fishers operating close to the Mekong and Red Rivers which are two of the most plastic-polluted rivers in the world. We found that the total incurred costs of plastic pollution in the three study regions were ~3400 USD per vessel per year, which is equivalent to more than ten percent of the annual revenue and approximately a quarter of the income of the vessel owners. Further harm to fishers was associated with injuries and fatalities when dealing with plastic entanglement incidents at sea. Our results also highlighted the disproportionate economic burdens faced by lower-income nearshore fishers in the Mekong Delta compared to those in the Red River Delta. The failure to manage incoming waste streams significantly erodes coastal livelihoods by shifting the burden of plastic pollution onto local communities.

Keywords: plastic pollution; marine litter; economic cost, sustainable fisheries, coastal communities, economic delta, development, Vietnam

Highlights:

- Fishing communities in Vietnam are significantly impacted by marine plastic pollution, which is compounded by other pressures.

- Consequences of marine plastic litter cost each fishing vessel >10% of the annual revenue and 25% of annual income for vessel owners.

- Mekong Delta fishers face greater economic burdens than those in the Red River Delta, revealing regional coastal inequities.

- Fishers have high level knowledge of marine plastic hotspots and a strong willingness to take collective action to mitigate marine plastic pollution.

- Failing to deal with the plastic waste issue will likely increase further the impact on coastal livelihoods and undermine sustainable development goals.

Main

Plastic debris and microplastics have been detected in almost every marine environment, from Arctic ice [1] to deep ocean trenches [2], entangling and accumulating in wildlife [3,4], and in human organs [5]. Numerous studies have identified the spatiotemporal magnitude of global marine plastic pollution, which encompasses plastic particles from metre to nanometre scales [6–8]. The majority of marine plastic debris entering oceans originates from major river systems in Asian countries[6,9], especially during extreme hydrological events[10,11], and becomes concentrated in coastal areas[12,13]. High coastal population density and the associated plastic leakage rates in Asian countries render this region as the one that bears the highest "price tag" of plastic pollution which is estimated to be 11 billion USD per year [14]. Nevertheless, we currently lack an understanding of the socioeconomic impacts of this plastic pollution on coastal communities with is primarily informed through modelling that do not accurately quantify the unseen impacts on communities and their well-being [15,16.]

Fishing activities[17] are estimated to contribute more than 10% of the plastic waste in the ocean[18]. Small-scale fisheries contribute over 40% of world catch and value, and play a vital role in nutrition and livelihoods, especially across Asia[19]. Viet Nam is one of the top five global exporters of fisheries products, valued at 10 billion USD in 2024 and fisheries are a significant contributor to national economic development, making up 4-5% of the national gross domestic product[20]. In 2023, 65% of Viet Nam's 86,820 fishing vessels operated in the nearshore fishing zone [21], mainly consisting of gillnetters and trawlers [21,22]. Despite the growing problem of plastic pollution along Viet Nam's coastline [23] there is limited understanding of the socioeconomic impact of marine plastic debris on coastal fishing communities and a lack of community voice on suitable interventions that might alleviate plastic pollution in a local context.

Commented [LP1]: Are those all small scale fisheries? Perhaps the authors can distinguish between large and small scale vessels if possible and if they think they can have significantly different contribution to plastic pollution in their study region.

We used a combination of quantitative and qualitative methods (See Supplementary Information F1 and methods) to quantify the cost of plastic pollution from the perspective of the affected fishing communities at three major fishing areas in Viet Nam, located at the outlets of the Red River and Mekong River[6]. This research provides important insights into the impacts of plastic pollution, which can help shape and implement realistic targets and policies at both local, national and international levels. It aims to advance marine plastics research by focusing on three key areas: (1) lack of data on impacts of plastics on coastal communities, (2) limitations of macroscopic models that rely on assumptions and lack empirical data from the impacted communities, and (3) potential of local perceptions to enhance such models, a factor that is often overlooked.

Commented [LP2]: And how does your study on Viet Nam contributed to those on a wider scale?

Results

Cost of marine plastic debris

Fishers (n=199) interviewed in our study incurred costs associated with plastic debris that were estimated at a median (Q1-Q3) value of 3421 (1848 - 6159) USD vessel^{-1} year^{-1} (Figure 1~~Figure 1~~) (See Methods and Supplementary Information for methodological details). This amount is equivalent to 12 (7 – 18) % of their annual revenue or 25 (15 - 48) % of the fishing vessel owner's income. The cost varied significantly among regions, costing 10% of the annual revenue of fishing vessels in Hai Phong and Nam Dinh in the Red River Delta to 18% of the annual revenue of vessels in Ben Tre in the Mekong Delta ($H_{2, 164}$=26.9, $P<0.0001$) (Figure 1d), almost doubling the impact of plastic in the region of the Mekong which is the area~~s~~ with the highest fishery production.

Commented [LP3]: Are these small scale fishers only?

Taken across all areas, the total economic cost of the impact of plastic on fishers comprised of 54% from lost revenue due to downtime that would otherwise be spent fishing, 28% from reduced catch efficiency, 12% from labour costs to remove plastics from the catch, 5% from fishing gear repair expenses, 1% from removing entangled plastics from propellers.

The total labour downtime caused by marine plastic debris was 158 (62 – 360) hours vessel^{-1} year^{-1}, equivalent to 6.5 (2-13)% of the total time spent fishing. Plastic debris accumulated in the catch and nets alone increased total labour time associated with sorting catch from plastic debris by 15 (8 - 30) minutes per 3-hour haul. Overall, 63% of the participants said they encountered marine plastics in their net almost every haul, and 18% said they frequently encountered plastics in 50-80% of their hauls.

Marine plastic debris also cause a high number of safety incidents while at sea such that, 45% of interviewees (n=90) reported having 4 (1 – 9) incidents per year of propeller entanglement with discarded or lost ropes and nets (See Supplementary Information T4). During focus group discussions (FGD) conducted in Nam Dinh and Ben Tre, fishers reported incidents where attempts to untangle debris from vessel propellers resulted in several cases of accidents or tragic fatalities. One fisher reported:

"*I have experienced it myself, my hands were cut by barnacles while trying to remove rope caught in my propeller; … in this area people have died or* [had limbs] *amputated while doing it.. they were pulled in the propeller when the old engine gear box malfunctioned…*".

During the FGD, another fisher stated:

"*There was one person … well, the person above* [on the deck of the vessel] *shifted the gear and the man below* [trying to remove entangled propeller] *was chopped to death by the propeller*".

Cross-validation of the cost estimates above was derived from follow-up semi-structured interviews with 94 fishers who did not participate in the first survey (See Supplementary Information F1**Error! Reference source not found.**). Among the interviewees, 77 % of fishers agreed, 16 % disagreed, and 7 % partially agreed with the findings regarding the total cost of marine plastic debris on fishers' incomes (**Error! Reference source not found.**~~See Supplementary Information T9~~). Those who partially agreed noted that marine debris does not equally affect all fishing vessels stating that nearshore vessels and trawlers were impacted the most.

Formatted: Highlight

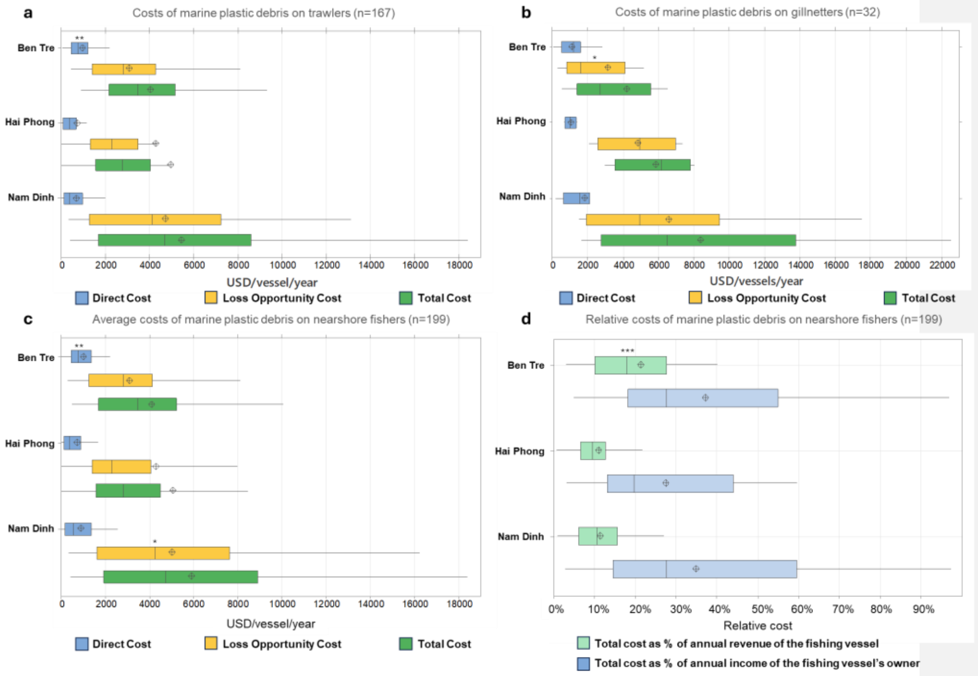

Figure 1. The economic cost of marine plastic debris on (a) trawlers, (b) gillnetters, (c and d) data from all participating fishing vessels in the study. Box and whisker plots show the distribution of cost across Hai Phong, Nam Dinh, and Ben Tre. The central line in each box represents the median; the bottom and top edges of the box represent the 25th and 75th percentiles (i.e. Q1 – Q3 or interquartile range, IQR). Whiskers extend to the minimum and maximum values within 1.5×IQR. Significant difference of each cost between the three study locations using Kruskal-Wallis test with *P<0.05, **P<0.01, ***P <0.001 (See Supplementary T1).

Commented [LP4]: I suggest the number of interviewees corresponding to the data is indicated in each figure.

Plastic debris composition based on fisher knowledge and waste audits

Fishers (n=195) reported that they catch approximately 15 (5 – 35) kg of mixed marine debris in each haul. They estimated that 17% [2 (1 - 5) kg] by weight of the marine debris in each haul was plastic (See Supplementary Information T4). The most frequently encountered plastic items were plastic bags, plastic bottles, single-use plastic items, fabrics, aquaculture feed bags, and ropes. Fishers (n=165) provided information about the geographical distribution of encounters with plastic debris, such that fishers reported that they encountered more plastic debris in either coastal zone (85% of responses) or the inshore area (13% of responses) with only 3% reporting more plastic in the offshore zone. Most fishers reported marine plastics hotspots at the outlets of rivers and agri/aqua-culture sluice gates, especially during the rainy season from May to October (See Supplementary Information T5).

Commented [LP5]: So the fishers' findings on plastic waste is also a mix of fishery-related and consumer plastic items, as were the findings from the waste audit. I would like this distinction to be more evident throughout the manuscript as to indicate that not all plastic waste affecting fishermen, and maybe not even most of it, comes from fishery activities.

The waste audit of marine debris caught by fishing vessels (Figure 4~~Figure 4~~) revealed that there were 1.3 (0.2 – 3.4) kg of mixed marine debris haul^{-1} and 0.6 (0.01 – 2.6) kg was plastic debris (**Figure 2**~~**Figure 2**~~). The heaviest marine debris recovered was predominantly associated with fishing activities, whereas the most frequently encountered items by count were consumer plastics, including both domestic and international brand labels (**Figure 3**~~**Figure 3**~~).

The participating fishers overestimated the weight of marine debris $(H_{1},\ _{477})=222$, P<0.0001) and plastic debris $(H_{1},\ _{477})=73$, P<0.0001) compared to the waste audit data by 11 and 3 times, respectively. Nevertheless, fishers demonstrated their local ecological knowledge (LEK) on marine plastic debris by reliably recalling the most common marine debris items and their increased abundance during the wet season, including the seasonal fluctuation in Ben Tre province (**Error! Reference source not found.**~~Table S5~~ and **Figure 2**~~**Figure 2**~~).

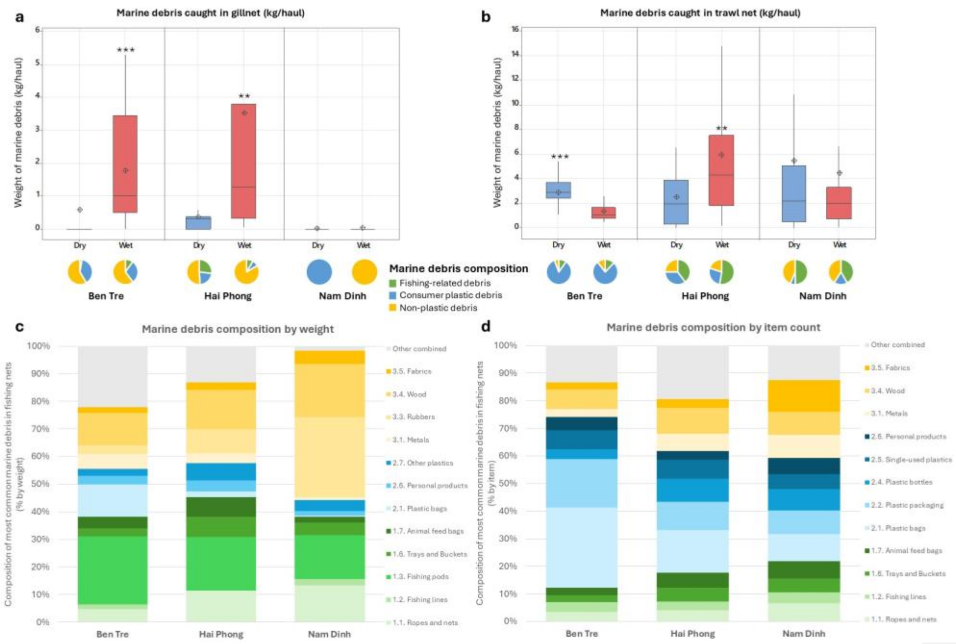

Figure 2. Waste audit of marine debris caught by nearshore fishing vessels in the studied areas. **a,b**: Weight (showing in box plot as kg/haul) and composition (showing in pie charts as average percentage by weight) of marine debris caught by gillnet (a, n=92) and trawl net (b, n=190) during dry and wet season (Method). The box and whisker plots with the central line in each box represents the median; the circle symbol represents the mean; the bottom and top edges of the box represent the 25th and 75th percentiles (i.e. Q1 – Q3 or interquartile range, IQR). Whiskers extend to the minimum and maximum values within 1.5×IQR. The scale of Figure 2a and 2b are not equal. Significant difference between marine debris caught in net between the dry and wet season is noted, analysed separately for each study location using Kruskal-Wallis test with *P<0.05, **P<0.01, ***P <0.001 (detailed statistics are available in Table S2, Supplementary Information). **c,d:** The twelve most dominant categories of marine debris caught in each haul ranked by weight (c, n=282, % by weight) and item count (d, n=282, % by item count). Green, blue, and yellow shades of colour represent fishing-related debris, consumer plastic debris, and non-plastic debris, respectively. ***Significant difference using Kruskal-Wallis test, P <0.001 (See Supplementary Information T2).

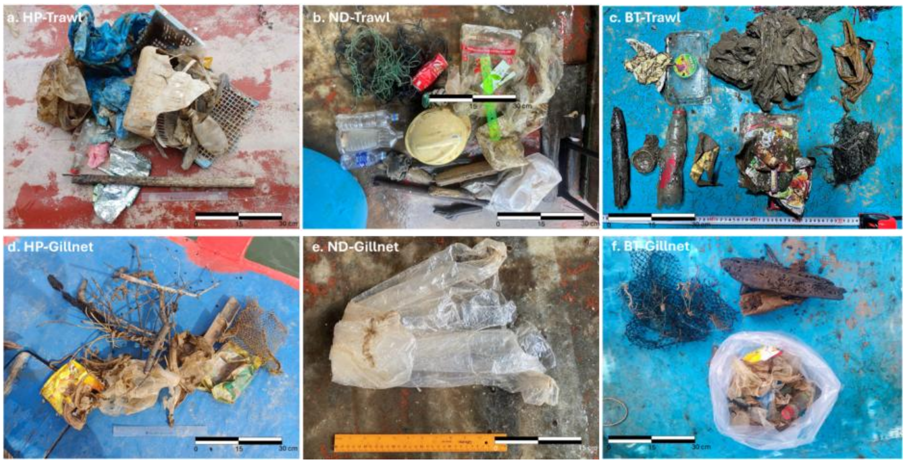

Figure 3: Photos of marine debris caught by nearshore fishing vessels in Hai Phong (HP), Nam Dinh (ND), and Ben Tre (BT). Marine debris caught in one single haul by trawlers (a,b,c) and gillnetters (d,e,f).

Fishers' perceptions of potential interventions

Participating fishers were aware of the potential negative impacts of marine plastic debris and abandoned, lost, or otherwise discarded fishing gear (ALDFG) on the environment, fish stocks, and their livelihoods (See Supplementary Information T7). Despite this awareness, 57% of fishers felt unable to take action to reduce the impact of marine plastics. The majority (77%) continued to discard marine debris caught in their nets back into the ocean, with 18% only retaining valuable items to sell or would bring back large objects to prevent future encounters. This mindset was encapsulated in a common sentiment:

"*I throw it back to the ocean, why should I bring it back. If I bring back but other people still throw the trash out, it will waste my efforts*" (See Supplementary Information T7).

The majority of fishers were interested in participating in a "fishing for litter" programme, whereby they would be compensated for collecting and bringing marine litter back to shore (n=196: 56% strongly agree and 23% agree). In general, fishers indicated that they would need to be paid a median buyback price of 0.16 (0.08 - 0.23) USD kg^{-1} of marine plastic debris, with no significant difference among gill net or trawl fishers ($H_{1,\ 119}$=0.34, p=0.6), although fishers in Hai Phong requested a higher marine litter buy-back price ($H_{1,\ 119}$=19.9, p<0.001) (See Supplementary Information T7).

Commented [LP6]: This is a very valuable information that was extracted from the surveys. It can be directly used towards mitigation measures, which are often hard to track.

Both the initial and follow-up surveys revealed that fishers consistently recommended a set of interventions to promote the return of marine debris to shore. These included implementing communication campaigns and visible signage to raise awareness about waste management at sea; establishing incentive schemes that allow fishers to exchange collected marine plastic for gifts or monetary rewards; introducing fines or penalties for discarding plastic waste at sea; enforcing clear regulations with robust monitoring mechanisms; and developing a coordinated waste collection programme supported by broad participation from all fishing vessels to ensure equity.

DISCUSSION

Inequities in the impacts of plastic pollution

Our findings demonstrate the substantial negative impact of marine plastic on the income, livelihoods and~~,~~ safety of nearshore trawl and gillnet fishers. The cost burden varied from 10 (6-16)% of annual revenue of fishing vessels in Hai Phong and Nam Dinh in the Red River Delta to 18 (10-28)% of annual revenue of vessels in Ben Tre in the Mekong Delta. Land-based marine plastics were found to be primarily confined to the coastal zone [13,24] and concentrated at outlets of major river systems such as the Mekong River in Ben Tre[6], hence coastal fishing communities bear the greatest cost burden compared with offshore fishers. Although the total absolute cost of plastic litter to fishing vessels is not significantly different between our case-study regions, the relative cost in terms of percentage of revenue loss was significantly higher for fishers in the Mekong Ben Tre province (*Figure 1*~~Figure 1~~c,d). This disparity is likely a result of nearshore fishers in Ben Tre that had significantly lower annual revenue (711 USD vessel^{-1}, $H_{2,\,198}$= 23.33, P<0.0001) and lower fishing yields (50 kg trip^{-1}, $H_{2,\,192}$=41.07, P<0.0001) than fishers in in the Red River Nam Dinh and Hai Phong provinces, making them more vulnerable to the additional economic impacts of marine plastic pollution.

Commented [LP7]: It was not clear to me whether this was their perceived or the actual, calculated cost burden. To me it would be useful to define this as readers will be more able to compare values with other studies.

Commented [LP8]: Is this trend observed in other parts of the world? A parallel with previously published literature can help position your work in a wider context, enhancing its relevance worldwide.

These regional differences are linked to a number of demographic factors. Smaller-scale fishers in Ben Tre were the most economically vulnerable to the impacts of plastic pollution because typically they had older vessels with smaller and less fuel-efficient engines, and had lower catch efficiency compared to fishers from the Red River Delta front, [25–27]. Furthermore, nearshore fishers in Ben Tre operated at the outlet of two of the world's most plastic-polluted rivers, the Mekong River and Saigon-Dong Nai river systems[6]. Plastic leakage to the coastal and nearshore environment in Ben Tre province is three times higher compared to the Red River, Hai Phong and Nam Dinh in the north of Viet Nam[28] (See Supplementary Information T6). This is reflected in our data from the waste audit which showed that significantly more plastic debris was caught in the fishing nets of fishers in Ben Tre compared to fishers in Nam Dinh (Kruskal-Wallis, $H_{2,283}$=15.92, P<0.001). Ben Tre fishers cannot move to alternative fishing grounds to avoid plastic pollution as fuel accounted for 64% of their operational costs. This was underlined by 53% of the interviewed fishers (n=68) who considered that they cannot do anything to alleviate the impact from marine debris;

"I have to accept it, what else can I do?"

Our study also revealed that marine debris results in serious accidents, especially when crew members must untangle fishing nets and ropes from the propeller. Although marine plastic-related accidents are rare and fishers often did not witness the incidents directly (making it difficult to verify whether the entangled ropes originated from their own fishing vessels or from ALDFG) the accidents reported emphasise the potential risks to nearshore fishers' health and safety[29]. Thus, plastic pollution in the coastal ocean has cumulative impacts on nearshore fishing communities thereby contributing to the lifetime cost of marine plastic pollution[30]. Marine plastic also compounds other

Commented [LP9]: Are there any reports of accidents in other regions of the world? I believe it is very important to emphasize these here, and bringing other examples would really help.

challenges confronting these fishers such as the impacts of extreme weather events, reductions in biodiversity, depletion of fish stocks, and conflicts over-fishing zones[31]. These interconnected issues intensify the pressure on vulnerable communities, making their coastal livelihoods increasingly precarious as river delta economies continue to grow and exacerbating the plastic waste problem.

Lack of comparable data among socioeconomic impact assessment studies

The current research on the socioeconomic impacts of marine plastic in Viet Nam and elsewhere is limited and lacks standardized data collection approaches[30]. Nevertheless, the estimates generated from this study are comparable to both empirical and large-scale modelling data for Viet Nam and other countries in the world (See Supplementary Information T8).

The direct cost estimated in this study is 0.6 to 3.6 times different from previous estimates for other provinces in Viet Nam (See Supplementary Information T8). The variability in estimates from elsewhere is associated with local demographics and environmental context. In addition, previous studies often only accounted for direct costs (i.e., gear damage, repair cost, time spent sorting catch from plastic) and did not include lost opportunity cost[32], which we do account for (i.e. potential revenue from time lost that could otherwise be spent fishing, reduction in catch due to interference with gear).

Commented [LP10]: Could the authors give more detail on other studies regarding their environmental and socioeconomical aspects that can differ from the present work?

Commented [LP11]: Could the authors please elaborate a bit more on why the inclusion of this factor onto cost calculation is important?

The lack of consistent reporting unit and economic estimation methods have been identified as one of the challenges in comparing the impacts of plastic pollution and ALDFG[33]. Detailed guidelines on quantifying marine debris and its impacts are therefore essential for quantifying pollution impacts[34]. Our study is an exemplar for how qualitative and quantitative data can be used to overcome the global gap of insufficient data on economic impact assessments for marine plastic pollution.

Using local knowledge to identify and mitigate plastic pollution hotspots

Identifying spatiotemporal hotspots of marine plastic debris is essential for guiding targeted mitigation and cleanup efforts. While numerical models can simulate large-scale and long-term plastic transport via ocean currents[35], they require validation through field observations, which are often costly and spatially or temporally limited. LEK from fishers, who spend extensive time at sea and rely on understanding marine dynamics for their livelihoods, offers a valuable resource to bridge these knowledge gaps and enhance hotspot detection.

Formatted: Highlight

Results from the waste audit (Figure 2~~Figure 2~~) and surveys with fishing communities (See Supplementary Information T5) were aligned, indicating that local knowledge was a valuable and reliable source of information to identify different plastic pollution hotspots of locations, seasons, items and potential polluters. The reported hotspots from fishers (See Supplementary Information T5) also agreed with the predictions of previous numerical simulation of transport of macroplastic exported from Ba Lat River in the Red River[13], implying that fishers' knowledge of plastic distribution reflects reality.

Formatted: Highlight

This local knowledge complements scientific data and, when combined, should be used to inform co-management for locating and tackling marine plastic debris[36,37]. On the other hand, once corroborated with local knowledge, field sampling data and plastics transport modelling may in turn guide fishers and coastal communities for a better practice and optimal locations for beach and ocean waste cleanup. For example, the numerical model [13] suggested that during the dry season, plastics were pushed back towards the shore, creating a potential window of opportunity to remove those plastics from the beach at a much lower cost than from the sea surface.

Solutions to mitigate impacts of plastic pollution on nearshore fishing communities

A preferred solution to actively engage nearshore fishers in studied areas in the marine pollution problem was an incentivised "fishing for litter" programme, where fishers were compensated for collecting marine plastic waste. Our data show that enrolment in such a programme should offer a median buyback price of only 0.16 USD kg^{-1} of marine plastic debris, or "*equivalent to the market price of trash fish*", as fishers often mentioned during the interviews.

Using waste audit data, each fishing boat is estimated to catch a median of 0.60 kg (0.01–2.55 kg) of marine plastic per haul, with a median of 630 hauls $year^{-1}$. A fishing-for-litter programme would therefore need to pay each participating vessel approximately 60 USD $year^{-1}$ to bring all collected marine plastic ashore (equivalent to 378 kg of plastic per vessel annually). The additional cost of treating the returned plastic would be around 20 USD $vessel^{-1}$ $year^{-1}$, assuming a waste treatment rate of 54 USD ton^{-1} in 2025 at an annual inflation rate of 5%[38]).

Such incentivised programs have been piloted in Viet Nam [39] and European countries [40,41] with initial success. However, these pilots often failed to consider socioeconomic inequalities when selecting implementation sites. Our study provides an important recommendation that the fishing for litter initiative should be prioritised at "plastic cost hotspots" to alleviate the economic impact of marine plastic on the most vulnerable fishing communities, including nearshore fishers in Ben Tre. We suggest that combining local knowledge from fishers (See Supplementary Information T5) with model simulations[13] could be applied more widely to inform a strategic plan for the prioritised time and place for coastal and marine debris cleanup.

Formatted: Highlight

Formatted: Highlight

Formatted: Highlight

Nearshore fishers interviewed in this study mentioned the importance of communication and the need for onshore waste collection systems through incentivised marine litter buyback programs. This position is echoed by studies in other regions in Viet Nam[37.] Sustainable solutions must also consider educational campaigns that explain the real cost of plastic litter, the benefits of recycling and other systemic changes. Such systems-change approaches should enhance both the "hardware" (physical/environmental changes) by increasing solid waste collection rates from upstream to downstream, and the "software" (societal changes) by altering social norms in solid waste management through legal and societal reinforcement. Co-management of marine resources has been identified as a key sustainable pathway to manage fisheries conflicts and maintain the livelihoods of coastal communities around the world[42,43].

CONCLUSION

Plastic pollution of coastal and marine environments is a persistent, pervasive, and costly challenge impacting nearshore fishers and their local communities the most. This study highlights the economic and social burdens faced by small-scale nearshore fishing communities in three delta front regions of the Mekong and the Red River in Viet Nam. Based on reports from 199 fishers and related stakeholders, the average cost on nearshore fishers is estimated to be 10%, 10.6%, and 18% of the annual revenue of each fishing vessel in the Red River Hai Phong City and Nam Dinh, and the Mekong River Ben Tre provinces, respectively.

The study identifies inequalities in the economic impacts of marine plastic debris. Nearshore fishers operating close to the Mekong estuaries face the strongest environmental and social stressors and were likely to experience greater impacts compared to Red River settings. Furthermore, fishers reported higher health and safety risks, particularly during the monsoon season, due to plastic entanglement incidents. Local fishers demonstrate a strong understanding of where and when hotspots of marine plastics may occur and proposed solutions that they are willing to participate if centrally managed. Fishers emphasised the need for interventions that promote cleanup marine plastic debris, through incentive programs or communication initiatives, and improved onshore waste collection systems, in combination effectively reducing negative impacts from marine plastic

Formatted: Highlight

Commented [LP12]: I do not recall this information from the results or discussion.

Formatted: Highlight

pollution. The new results from this study serve as components of a framework for identifying economic impact hotspots and co-designing effective solutions with community participation to tackle marine plastic pollution in coastal areas worldwide.

Formatted: Highlight

METHODS

Location and scope of study

This study focused on small-scale nearshore fishing communities in three regions: Hai Phong City (HP), Nam Dinh Province (ND), and Ben Tre Province (BT) (Figure 4~~Figure 4~~). These sites were selected for their contrasting geographical and socioeconomic characteristics (*Supplementary Information*). HP represents a coastal and island dominated area with high income, important fishing landing sites, maritime shipping industries, and tourism destinations. ND and HP are located at the outlets of the Red River in the north. BT is adjacent to the Mekong River in the south of Viet Nam and is characterised by small-scale fisheries, aquaculture, agriculture, and other coastal businesses. Previous studies show that these two rivers are among the top rivers in the world with regard to discharge of plastic waste into the coastline and ocean [6,44].

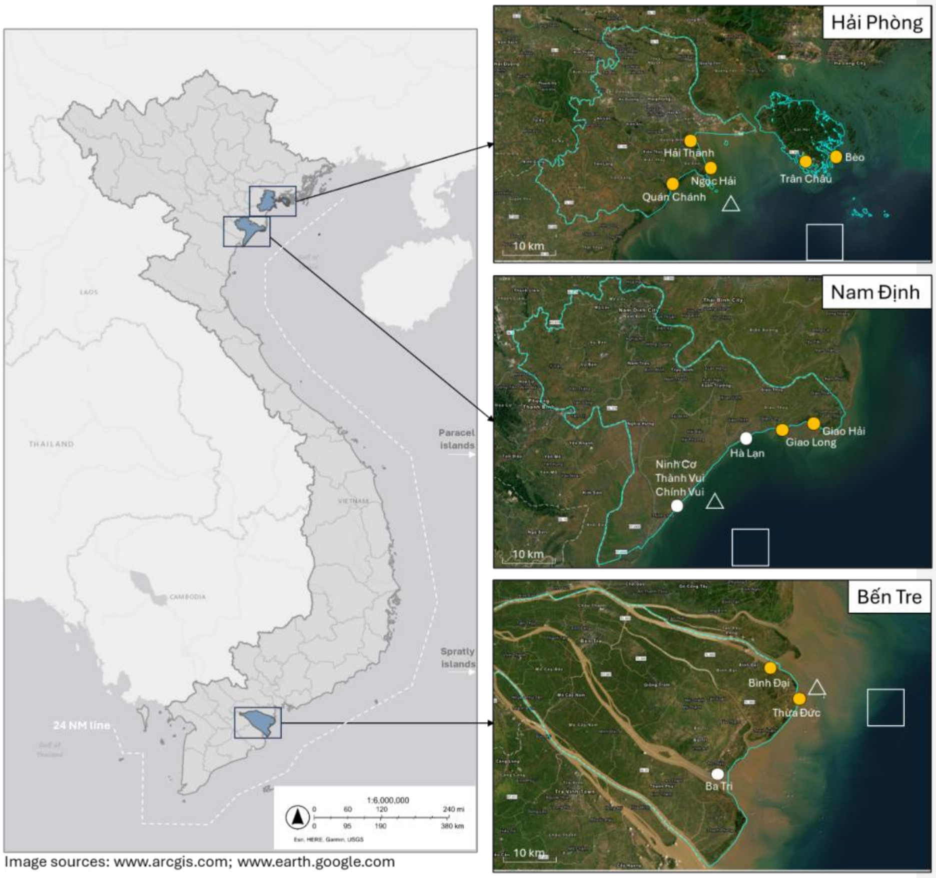

Figure 4: Locations of the study areas in Hai Phong, Nam Dinh, and Ben Tre. Yellow dots are the locations of fishing ports and communities involved in the main survey (n = 199 fishers); white dots are additional fishing port locations included in the post-survey validation (n = 94 fishers). Triangles

show the locations of waste audits on gillnetters (n = 92 observations); rectangles indicate the locations of waste audits on trawlers (n = 190 observations)."

In this study, the term "nearshore" refers to both coastal and inshore fishing zones which are defined by Vietnamese law to include the area from the shore out to 24 nautical miles. Previous research indicates that globally up to 77% of buoyant ocean plastics are concentrated in the coastal zone[24] hence this study should face the highest impact of plastic waste to the environment and coastal communities. We focused on small-scale fishing vessels owned and operated by local families using trawls and gillnets in nearshore waters that are disproportionately impacted by plastic debris [22,37,45].

Commented [LP13]: In Viet Nam or globally?

Commented [LP14]: The readers could benefit from having a reminder of this in the results section as it comes further up the main text.

Formatted: Highlight

Data collection

The study was conducted following a methodology of mixed methods[14] that were implemented in a consistent sequence at each field site **Error! Reference source not found.**. Participants were selected through a combination of snowball sampling, initiated with key informants (e.g., governmental officers from the Department of Fisheries or leaders of fishing groups within the commune), and random sampling with fishers at different ports (Figure 4~~Figure 4~~), allowing us to collect data from a total of 308 individuals. A total of fifteen semi-structured interviews with fisheries governmental officers (n=9) and fishing port managers (n=6), and three focus group discussions (FGDs) (n=8 participants each) with fishers were conducted across the three field sites. The first survey consisted of 199 structured interviews with nearshore fishers to quantify the economic impacts of marine plastic debris on their livelihoods. A follow-up survey using structured interviews was conducted with fishers who had not participated in the first survey (n=94) to assess 1) the extent of agreement or disagreement that fishers had with the research findings and 2) their perspectives on the suitable solutions. Detailed data collection procedure, calculation methods, and data limitations are available in the Supplementary Information**Error! Reference source not found.**.

Cost estimation

Using the data from the first survey with 199 fishers, the economic costs were calculated with the following formula:

Total Cost (TC*)* = Direct cost (DC=DC1+DC2+DC3) + Lost opportunity cost (LC=LC1+LC2)

Where:

DC1 = Labour cost of crew members' combined time spent separating plastic debris from fishing gear and the catch

DC2 = Labour cost of crew member's combined time spent untangling plastic debris from propellers, cooling water inlets etc.

DC3 = Cost of replacing and fixing damaged gears from encountering plastic debris and/or towing vessel to port for repairing if the vessel was disabled due to plastic debris

LC1 = Loss of revenue from reducing fishing efficiency due to plastic debris by tearing nets, blocking nets, and causing nets to float, among other issues.

LC2 = Loss of revenue from total time lost when the fishing vessel could have been fishing

The costs incurred from encounters with marine plastic debris were calculated individually for each vessel, based on the fishers' responses and estimates. Each of the five components to the total cost was calculated based on multiple contributing factors, including: type of plastic-related incidents (non-exhaustive list includes: stuck in fishing nets, mix in the catch, entangle in propeller, block water

inlet, damage fishing net, etc.), frequency of each incident (incidents per year), time loss (hours per incident), number of people involved (people per incident), labour cost (USD per working hour), repair cost (USD per incident), estimated reduction in catch efficiency (% reduction), revenue per unit effort (USD per hour fishing), and estimated plastic content in mixed marine debris (% by weight). Details on the calculation of cost is available in Supplementary Information.

The direct cost represents expenses that fishers could recall in detail, making them more reliable. The lost opportunity cost uses fishers' estimation of catch efficiency reduction due to plastic debris and loss of revenue per unit time loss, and therefore subjects to a higher degree of uncertainty and fluctuations [25,32].

Waste Audit

A waste audit was conducted on board the fishing vessels by science observers following the method adapted from [46] (Detail in SI1). A total of six small-scale fishing vessels (one trawler and one gillnetter for each of the three study areas) were contracted to participate in the study on the spatiotemporal distribution of marine litter in nearshore waters. In total, we collected 282 observations (190 hauls from trawlers and 92 hauls from gillnetters), split between three study areas and two seasons (Dry season: 17th April to 30th May, 2024; Wet season: 1st August to 30th September, 2024). The same vessel and gear were used in both sampling periods to compare seasonal variability. However, the fishing gear in the three locations had slight differences. The bottom trawlers had nets with a mesh size of 25–35 mm and operated at a maximum water depth of 10–36 m, while the gillnetters used nets with a mesh size of 45–55 mm and were deployed at a water depth of 5–10 m from the water surface.

Data analyses

Anonymized interview responses were entered, coded and screened for missing data and invalid answers (i.e. blank and "I don't know" answers). Audio recordings of the semi-structured interviews and FGDs were transcribed, translated, and coded in NVivo 14 (Version 14.23.2 (46)). Data analysis was conducted using IBM SPSS Statistics software (Version 29.0.1.0 (171)). The estimated costs and other data reported by interviewers were non-normally distributed (Shapiro–Wilk test, $p < 0.001$). Therefore, we reported all values as median (Q1 - Q3). Significant differences were tested using Kruskal–Wallis test with the test statistic H-value (degree of freedom (df), total samples (n)), presented as $H_{df, n}$ and associated P-values.

OTHER SECTIONS

CRediT authorship contribution statement

Duc Nguyen: Methodology, Data curation, Visualisation, Investigation, Writing.

Phan Phuong Thanh: Investigation, Data curation, Writing – review & editing

Heidi Burdett: Methodology, Funding acquisition, Writing – review & editing

Michel Kaiser: Project lead, funding acquisition, conceptualisation, Writing – review & editing

Thomas Wagner: Conceptualisation**,** Writing – review & editing

Ryan Pereira: Writing – review & editing

Inna Yavana-Toraman: Methodology, Writing – review & editing

Zhiling Liao: Writing – review & editing

Qingping Zou: Writing – review & editing

Trinh Quang Tu, Le Trung Dung, Le Thi Thu Huong: Investigation

Data availability

Data will be made available upon request.

Ethics declaration

Data in this study was collected and protected following the Equality and Privacy Impact Assessment and Data Management Plan approved for the project "Sources, Sinks and Solutions for Impacts of Plastics on Coastal Communities in Viet Nam" (NE/V006088/1). The research was subject to full ethics approval through the Heriot-Watt University ethics committee: approval number 2023-3366-7400.

Acknowledgement

This research was funded by the UKRI-NERC through Global Challenges Research Fund (GCRF) programme (NE/V006088/1). We thank all the fishers, informants, and other participants for contributing their time and knowledge that made this study.

REFERENCES

1. Bergmann, M. *et al.* Plastic pollution in the Arctic. *Nat Rev Earth Environ* **3**, 323–337 (2022).
2. Abel, S. M., Wu, F., Primpke, S., Gerdts, G. & Brandt, A. Journey to the deep: plastic pollution in the hadal of deep-sea trenches. *Environmental Pollution* **333**, 122078 (2023).
3. Omeyer, L. C. M. *et al.* Interactions between marine megafauna and plastic pollution in Southeast Asia. *Science of the Total Environment* **874**, 162502 (2023).
4. Ugwu, K., Herrera, A. & Gómez, M. Microplastics in marine biota: A review. *Mar Pollut Bull* **169**, (2021).

5. Liu, S. *et al.* Detection of various microplastics in placentas, meconium, infant feces, breastmilk and infant formula: A pilot prospective study. *Science of the Total Environment* **854**, 158699 (2023).

6. Meijer, L. J. J., van Emmerik, T., van der Ent, R., Schmidt, C. & Lebreton, L. More than 1000 rivers account for 80% of global riverine plastic emissions into the ocean. *Sci Adv* **7**, (2021).

7. Stubbins, A., Law, K. L., Muñoz, S. E., Bianchi, T. S. & Zhu, L. Plastics in the Earth system. *Science (1979)* **373**, 51–55 (2021).

8. Velis, C., Cottom, J. & Cook, E. A local-to-global emissions inventory of macroplastic pollution. *Nature* **633**, (2023).

9. Lebreton, L. C. M. *et al.* River plastic emissions to the world's oceans. *Nat Commun* **8**, 1–10 (2017).

10. van Emmerik, T., Mellink, Y., Hauk, R., Waldschläger, K. & Schreyers, L. Rivers as Plastic Reservoirs. *Frontiers in Water* **3**, 1–8 (2022).

11. van Emmerik, T. H. M. *et al.* River plastic transport and deposition amplified by extreme flood. *Nature Water* **1**, 514–522 (2023).

12. Morales-Caselles, C. *et al.* An inshore–offshore sorting system revealed from global classification of ocean litter. *Nat Sustain* **4**, 484–493 (2021).

13. Liao, Z., Zou, Q., Duy, V., Pan, Z. & Kaiser, M. J. Seasonal change in fate and transport of plastics from Red River to the coast of Vietnam. *Mar Pollut Bull* **208**, 116923 (2024).

14. Ramírez, D. M., Sachs, A. L. & Ekenga, C. C. Qualitative and mixed methods: informing and enhancing exposure science. *J Expo Sci Environ Epidemiol* (2024) doi:10.1038/s41370-024-00707-5.

15. Richardson, K., Wilcox, C., Vince, J. & Hardesty, B. D. Challenges and misperceptions around global fishing gear loss estimates. *Mar Policy* **129**, 104522 (2021).

16. Aretoulaki, E., Ponis, S., Plakas, G. & Agalianos, K. Marine Plastic Littering: a Review of Socio Economic Impacts. *J Sustain Sci Manag* **16**, 277–301 (2021).

17. Lebreton, L. *et al.* Industrialised fishing nations largely contribute to floating plastic pollution in the North Pacific subtropical gyre. *Sci Rep* **12**, 1–11 (2022).

18. Richardson, K., Hardesty, B. D. & Wilcox, C. Estimates of fishing gear loss rates at a global scale: A literature review and meta-analysis. *Fish and Fisheries* **20**, 1218–1231 (2019).

19. Basurto, X. *et al.* Illuminating the multidimensional contributions of small-scale fisheries. *Nature* **637**, 875–884 (2025).

20. VASEP. Fishery profile. https://seafood.vasep.com.vn/why-buy-seafood/fishery-profile (2025).

21. MARD. *REPORT ON PLANNING FOR THE PROTECTION AND UTILISATION OF FISHERIES RESOURCES FOR THE PERIOD 2021-2030, WITH A VISION TOWARD 2050 (In Vietnamese)*. (2024).

22. WWF-Viet Nam. *Khảo Sát Quốc Gia về Sự Đóng Góp Của Rác Thải Nhựa Từ Hoạt Động Khai Thác và Nuôi Trồng Thủy Sản Đến Rác Thải Nhựa Đại Dương*. (2020).

23. Veettil, B. K., Hua, N. T. A., Van, D. D. & Quang, N. X. Coastal and marine plastic pollution in Vietnam: Problems and the way out. *Estuar Coast Shelf Sci* **292**, 108472 (2023).

24. Onink, V., Jongedijk, C. E., Hoffman, M. J., van Sebille, E. & Laufkötter, C. Global simulations of marine plastic transport show plastic trapping in coastal zones. *Environmental Research Letters* **16**, (2021).

25. Ngoc, Q. T. K., Xuan, B. B., Duy, N. N. & Börger, T. The Impact of Marine Litter on Production Risk and Technical Efficiency in the Trawl Fisheries of Vietnam. *Marine Resource Economics* **38**, 65–84 (2023).

26. Nguyen, Q., Pascoe, S. & Coglan, L. Implications of regional economic conditions on the distribution of technical efficiency: Examples from coastal trawl vessels in Vietnam. *Mar Policy* **102**, 51–60 (2019).

27. Pomeroy, R. *et al.* Fish wars: Conflict and collaboration in fisheries management in Southeast Asia. *Mar Policy* **31**, 645–656 (2007).

28. WWF-Viet Nam. *Report on Plastic Waste Generation in 2022*. (Thanh Nien Publishing House, Hanoi, 2023).

29. Kim, B., Lee, H. & Kim, T. Possibility analysis of complex marine accidents caused by large marine plastic litter in the Republic of Korea. *Mar Policy* **152**, 105591 (2023).

30. WWF. *Plastics: The Costs To Society, the Environment and the Economy*. www.panda.org (2021).

31. Beaumont, N. J. *et al.* Global ecological, social and economic impacts of marine plastic. *Mar Pollut Bull* **142**, 189–195 (2019).

32. Rodríguez, Y., Ressurreição, A. & Pham, C. K. Socio-economic impacts of marine litter for remote oceanic islands: The case of the Azores. *Mar Pollut Bull* **160**, 111631 (2020).

33. Mengo, E. *et al.* Fishers' views and experiences on abandoned, lost or otherwise discarded fishing gear and end-of-life gear in England and France. *Mar Pollut Bull* **194**, 115372 (2023).

34. Walker, T. R., McGuinty, E. & Hickman, D. Marine debris database development using international best practices: A case study in Vietnam. *Mar Pollut Bull* **173**, 112948 (2021).

35. Liao, Z., Zou, Q., Vinh, V. D., Pan, Z. & Kaiser, M. J. Seasonal change in fate and transport of plastics from Red River to the coast of Vietnam. *Mar Pollut Bull* **208**, (2024).

36. Truchet, D. M. *et al.* Fishers' ecological knowledge about marine pollution: What can fek contribute to ecological and conservation studies of a southwestern atlantic estuary? *Herpetologica* **75**, 584–606 (2019).

37. Xuan, B. B., Ngoc, Q. T. K. & Börger, T. Fisher preferences for marine litter interventions in Vietnam. *Ecological Economics* **200**, (2022).

38. The World Bank. Solid and Industrial Hazardous Waste Management Assessment - Options and action area to implement the national strategy. 107 (2018).

39. Quertamp, F., Cương, N. T., Long, N., Viên, H. & Tố, N. C. Rethinking Plastics - Circular Economy Solutions to Marine Litter " FISHING FOR LITTER " SUMMARY I . Project information II . Objectives III . Achievements IV . General assessment V . Recommendations. 1–19 (2022).

40. Forleo, M. B. & Romagnoli, L. Fishing for litter for the reduction of marine plastic debris: What benefits and costs do Italians perceive? *Mar Pollut Bull* **192**, 115018 (2023).

41. Wyles, K. J., Pahl, S., Carroll, L. & Thompson, R. C. An evaluation of the Fishing For Litter (FFL) scheme in the UK in terms of attitudes, behavior, barriers and opportunities. *Mar Pollut Bull* **144**, 48–60 (2019).

42. Apete, L., Martin, O. V. & Iacovidou, E. Fishing plastic waste: Knowns and known unknowns. *Mar Pollut Bull* **205**, 116530 (2024).

43. Pomeroy, R., Thi Nguyen, K. A. & Thong, H. X. Small-scale marine fisheries policy in Vietnam. *Mar Policy* **33**, 419–428 (2009).

44. Vriend, P. & Bak, M. P. River export of macro- and microplastics to seas by sources worldwide. (2023) doi:10.1038/s41467-023-40501-9.

45. Raes, L., Jain, A., Ba, T. N. & Savels, R. The economic impact of marine plastics, including ghost fishing, on fishing boats in Phước Tinh and Loc An, Ba Ria Vung Tau Province, Viet Nam. (2022).

46. GESAMP. *Guidelines for the Monitoring and Assessment of Plastic Litter in the Ocean*. *GESAMP* vol. R99 http://www.gesamp.org/publications/guidelines-for-the-monitoring-and-assessment-of-plastic-litter-in-the-ocean (2019).

Marine plastic pollution undermines the livelihoods and income of fishing communities in Viet Nam

Duc Nguyen[1], Phan Phuong Thanh[2], Heidi L. Burdett[3,4], Inna Yaneva-Toraman[1], Zhiling Liao[1], Qingping Zou[1], Trinh Quang Tu[2], Le Trung Dung[2], Le Thi Thu Huong[2], Ryan Pereira[1], Thomas Wagner[1], Michel J. Kaiser[1*]

Affiliations:

1 The Lyell Centre, Heriot-Watt University, Riccarton, Edinburgh, United Kingdom

2 Viet Nam Institute of Fisheries Economics and Planning, 10 Nguyen Cong Hoan Street, Ba Dinh District, Hanoi, Viet Nam

3 Umeå Marine Sciences Centre, Umeå University, Norrbyn, Sweden

4 Department of Ecology, Environment and Geoscience, Umeå University, Umeå, Sweden

*Corresponding author: m.kaiser@hw.ac.uk

ABSTRACT

Globally, small-scale fishing communities are impacted by increasing quantities of plastic debris in coastal waters. We studied the socioeconomic impacts of marine plastic pollution on fishers operating near the Mekong and Red Rivers, Viet Nam, which are two of the most plastic-polluted rivers in the world. We used a mixed methods approach to quantify the impacts of marine plastics based on structured interviews with nearshore fishers (n = 199), thereafter validated with post-hoc fisher interviews (n = 94) and waste audits of plastics caught in fishing nets (n = 282) in the same study areas. We calculated that the total costs incurred from plastic pollution in the study regions were equivalent to ~10% of the annual revenue and ~25% of the vessel owners' income. Fishers experienced injuries and fatalities when dealing with plastic entanglement incidents at sea. Economic burdens faced by lower-income nearshore fishers in the Mekong Delta were higher compared to those in the Red River Delta. Incoming waste streams significantly eroded coastal livelihoods by shifting the burden of plastic pollution onto local communities. Incentive-based plastic collection systems at prioritised locations where plastic pollution most affects coastal communities would be effective at reducing marine pollution while supporting sustainable livelihoods.

Keywords: plastic pollution; marine litter; economic cost, sustainable fisheries, coastal communities, social impact, fishers, incentives, regional inequities

Highlights:

- Fishing communities in Viet Nam are significantly impacted by marine plastic pollution, which is compounded by other pressures.

- Consequences of marine plastic litter cost each fishing vessel >10% of the annual revenue and 25% of annual income for vessel owners.

- Mekong Delta fishers face greater economic burdens than those in the Red River Delta, revealing regional coastal inequities.

- Fishers have a high level of knowledge about marine plastic hotspots and a strong willingness to take collective action to mitigate marine plastic pollution.

- Failing to deal with the plastic waste issue will likely further increase the impact on coastal livelihoods and undermine sustainable development goals.

Introduction

Plastic debris and microplastics have been detected in almost every marine environment, from Arctic ice [1] to deep ocean trenches [2], entangling and accumulating in wildlife [3,4], and in human organs [5]. Numerous studies have identified the spatiotemporal magnitude of global marine plastic pollution, which encompasses plastic particles from metres to nanometres [6–8]. The majority of marine plastic debris entering oceans originates from major river systems in Asian countries[6,9], especially during extreme hydrological events[10,11], and becomes concentrated in coastal areas[12,13]. Even though plastic pollution is a global issue, the plastic waste trade[14] and export to Asian countries with high coastal population densities and associated plastic leakage rates render this region as the one that bears the highest "price tag" of plastic pollution, which is estimated to be 11 billion USD per year[15]. The lifetime socio-economic cost of marine plastic pollution in 2019 was estimated at 3.7 trillion USD and is projected to exceed 7.1 trillion USD by 2040 [16]. These costs were modelled based on quantifiable costs from greenhouse gases, waste management, and the impacts of mismanaged waste on the marine ecosystem. Globally, the direct economic impact of plastic pollution on tourism, fisheries, and governance along coastlines and waterways was 2 USD per capita per year, with 82% attributed to cleanup costs and 18% to revenue loss[17]. Nevertheless, we currently lack a detailed understanding of the socioeconomic impacts of this plastic pollution on coastal communities, which at present is primarily informed through modelling that does not accurately quantify the unseen impacts on communities and their well-being [18,19]. Here, we address the key knowledge gaps in existing macro-scale estimation models by providing empirical-based costs of marine plastic debris on the incomes and livelihoods of fishing communities.

Fishing activities[20] are estimated to contribute more than 10% of the plastic waste in the ocean[21]. Small-scale fisheries contribute over 40% of the world's catch and value, and play a vital role in nutrition and livelihoods, especially across Asia[22]. Viet Nam is one of the top five global exporters of fisheries products, valued at 10 billion USD in 2024, and fisheries are a significant contributor to national economic development, making up 4-5% of the national gross domestic product[23]. In 2023, 65% of Viet Nam's 86,820 fishing vessels operated in the nearshore fishing zone [24], mainly consisting of gillnetters and trawlers [24,25]. Despite the growing problem of plastic pollution along Viet Nam's coastline [26] there is limited understanding of the socioeconomic impact of marine plastic debris on coastal fishing communities[27] and a lack of community voice regarding suitable interventions that might alleviate plastic pollution in a local context.

We used a combination of quantitative and qualitative methods to quantify the cost of plastic pollution from the perspective of the affected fishing communities at three major fishing areas in Viet Nam,

located at the outlets of the Red River and Mekong River[6]. This research provides important insights into the impacts of plastic pollution, which can help shape and implement realistic targets and policies at both local, national and international levels. It aims to advance marine plastics research by focusing on two key areas: (1) lack of data on the socioeconomic impacts of plastics on coastal communities, (2) limitations of macroeconomic models that rely on assumptions and overlook empirical data from impacted communities.

Results

Cost of marine plastic debris

This study used a mixed-methods approach, including structured interviews with fishers (n=199), focus group discussions (n=3 with 8 participants each), and semi-structured interviews with port managers (n=6) and fisheries officers (n=9) across three nearshore regions of Hai Phong, Nam Dinh, and Ben Tre, at the outlets of the Red and Mekong Rivers in Viet Nam (Figure 4 and 5). Reported data were analysed to estimate the economic and societal costs of marine plastic debris on small-scale nearshore fisheries. The socio-economic impact assessment and fishers' ecological knowledge of marine plastic pollution were further complemented by verification surveys with different fishers (n=94) and waste audits of debris caught in fishing nets (n=282) in the same geographic areas. The main findings are presented below, with detailed provincial and fishing gear-specific data provided in the Supplementary Information.

The fishers (n=199) interviewed in our study incurred costs associated with plastic debris that were estimated to have a median (Q1-Q3) value of 3421 USD (1848 - 6159 USD) vessel^{-1} year^{-1} (**Figure *1***). This amount is equivalent to 12% (7 – 18%) of their annual revenue or 25% (15 - 48%) of the fishing vessel owner's income. The cost varied significantly among regions, costing 10% of the annual revenue of fishing vessels in Hai Phong and Nam Dinh in the Red River Delta compared with 18% of the annual revenue of vessels in Ben Tre in the Mekong Delta ($H_{2,164}$=26.9, P<0.0001) (Figure 1d), almost doubling the impact of plastic in the region of the Mekong which is the area with the highest fishery production.

The estimated total cost for trawl and gillnetting vessels was not significantly different ($H_{1,199}$=1.48, P=0.4) with the median annual loss of 3356 and 4967 USD vessel^{-1} year^{-1}, respectively (See Figure 1a-b). The economic impacts of marine plastic debris expressed as lost annual revenue were 18% (9-29%) for gillnetters and 11% (7-18%) for trawlers in our study.

Taken across all study areas, the contribution to the total economic cost of the impact of plastic on fishers comprised of 54% from lost revenue due to downtime that would otherwise be spent fishing, 28% from reduced catch efficiency, 12% from labour costs to remove plastics from the catch, 5% from

fishing gear repair expenses, 1% from removing entangled plastics from propellers (See Supplementary Table 1).

The total labour downtime caused by marine plastic debris was 158 (62 – 360) hours vessel^{-1} year^{-1}, equivalent to 6.5% (2-13%) of the total time spent fishing (See Supplementary Table 4). Plastic debris accumulated in the catch and nets alone increased total labour time associated with sorting catch from plastic debris by 15 (8 - 30) minutes per 3-hour haul. Overall, 63% of participants reported encountering marine plastics in their nets almost every haul, and 18% reported encountering plastics frequently in 50-80% of their hauls.

Marine plastic debris also caused a high number of safety incidents at sea. Among the 199 fishers interviewed, 45% (n = 90 fishers) reported propeller entanglement with discarded or lost ropes and nets, with a median of 4 (1–9) incidents per year. (See Supplementary Table 4). During focus group discussions (FGD) conducted in Nam Dinh and Ben Tre, fishers reported incidents where attempts by fishers to untangle debris from vessel propellers resulted in several cases of serious injuries or fatalities. One fisher reported:

"*I have experienced it myself, my hands were cut by barnacles while trying to remove rope caught in my propeller; … in this area people have died or* [had limbs] *amputated while doing it.. they were pulled in the propeller when the old engine gear box malfunctioned…*".

During the FGD, another fisher stated:

"*There was one person … well, the person above* [on the deck of the vessel] *shifted* [the engine into] *gear and the man below* [trying to remove entangled propeller] *was chopped to death by the propeller*".

Verification of the cost estimates above was derived from semi-structured validation interviews with 94 fishers who did not participate in the first survey (See Supplementary Figure 1). Among the interviewees, 77% of fishers agreed, 16% disagreed, and 7% partially agreed with the findings regarding the total cost of marine plastic debris on fishers' incomes (Supplementary Table 9). Those who partially agreed noted that marine debris does not equally affect all fishing vessels, stating that nearshore vessels and trawlers were impacted the most.

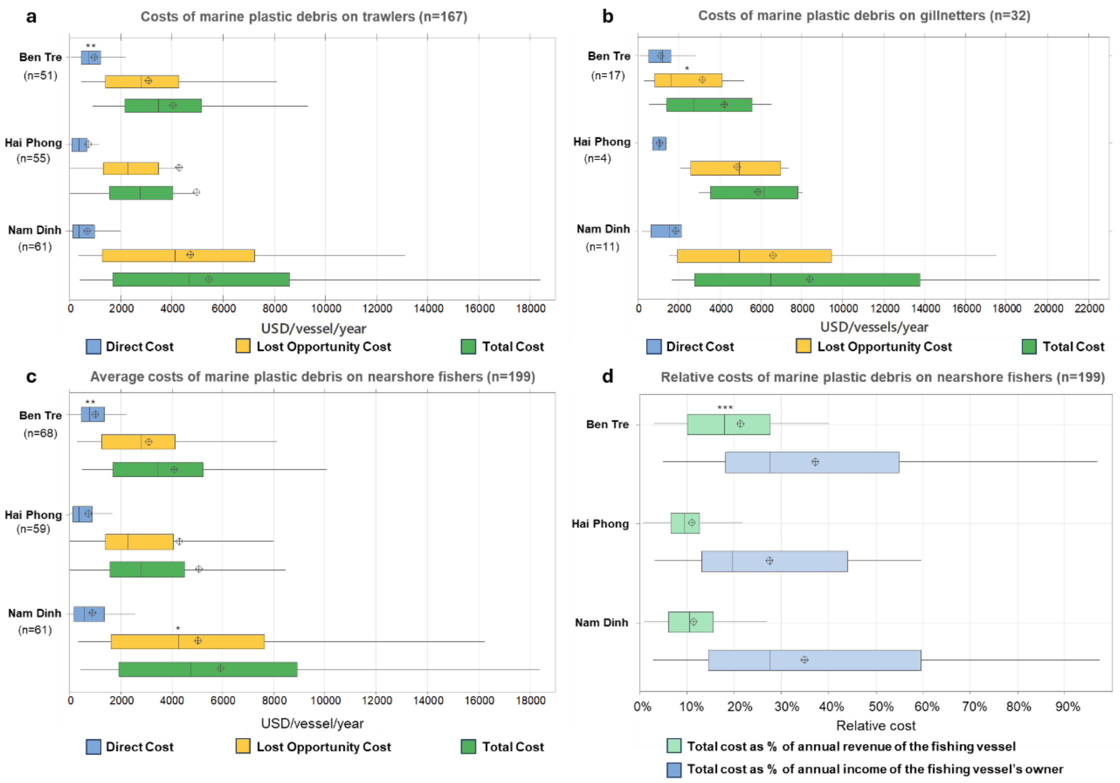

Figure 1. The economic cost of marine plastic debris on fishing vessels. The costs associated with marine plastic debris were calculated for (a) trawlers, (b) gillnetters, (c and d) all participating fishing vessels in the study. Box and whisker plots show the distribution of costs across Hai Phong, Nam Dinh, and Ben Tre. The central line in each box represents the median; the bottom and top edges of the box represent the 25th and 75th percentiles (i.e. Q1 – Q3 or interquartile range, IQR). Whiskers extend to the minimum and maximum values within 1.5×IQR. Significant difference of each cost between the three study locations using Kruskal-Wallis test with *P<0.05, **P<0.01, ***P <0.001 (See Supplementary Table 1 for detailed test statistics).

Plastic debris composition based on fisher knowledge and waste audits

Fishers (n=195) reported that they catch a median of approximately 15 kg (5 – 35 kg) of mixed marine debris in each haul. They estimated that 17% [2 (1 - 5) kg] by weight of the marine debris in each haul was plastic (See Supplementary Table 4). The most frequently encountered plastic items were plastic bags, plastic bottles, single-use plastic items, fabrics, aquaculture feed bags, and ropes. Fishers (n=165) provided information about the geographical distribution of encounters with plastic debris, such that fishers reported that they encountered more plastic debris in either the coastal zone (85% of responses) or the inshore area (13% of responses), with only 3% reporting more plastic in the offshore zone. Most fishers reported that marine plastics hotspots occurred at rivers outlets and agri/aqua-

culture sluice gates, especially during the rainy season from May to October, when plastic transport from the land is greatest (See Supplementary Table 5).

The waste audit of marine debris caught by fishing vessels (Figure 4) revealed that there were 1.3 (0.2 – 3.4) kg of mixed marine debris haul^{-1}, and 0.6 kg (0.01 – 2.6 kg) was plastic debris (Figure 2). The heaviest marine debris recovered was predominantly associated with fishing activities, whereas the most frequently encountered items by count were consumer plastics, including both domestic and international brand labels (**Figure 3**).

Although the participating fishers overestimated the weight of marine debris ($H_{1,\ 477}$ =222, P<0.0001) and plastic debris ($H_{1,\ 477}$ =73, P<0.0001) compared to the waste audit data by 11 and 3 times, respectively, it is worth noting that their estimate of plastic debris is much more accurate than their perception of overall marine debris in their catches. Nevertheless, fishers demonstrated the reliability of their local ecological knowledge (LEK) regarding marine plastic debris by accurately recalling the most common marine debris items and their increased abundance during the wet season, including seasonal fluctuations in Ben Tre province (Figure 2 and Supplementary Table 5).

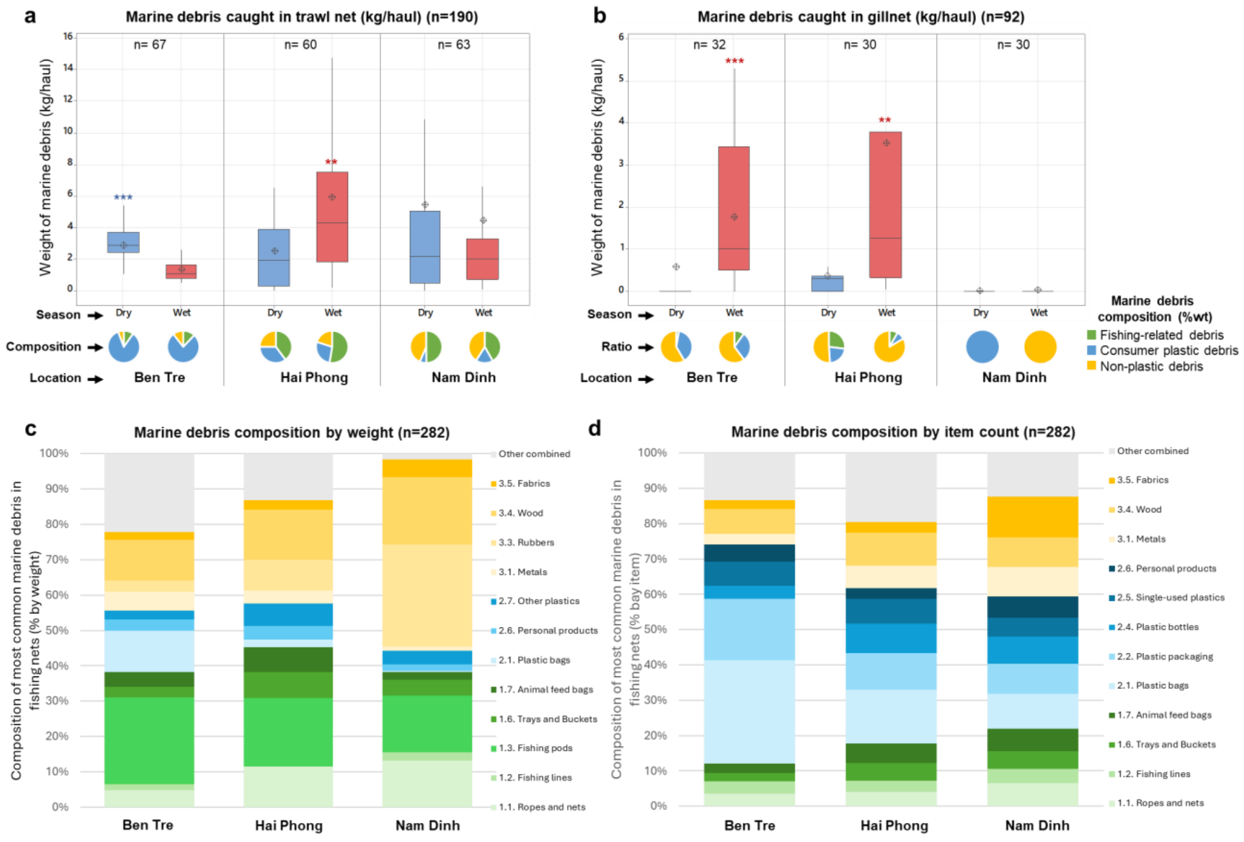

Figure 2. Waste audit of marine debris caught by nearshore fishing vessels in the studied areas during the dry and wet seasons. a,b: Weight (showing in box plot as kg/haul) and composition (showing in pie charts as average percentage by weight) of marine debris caught by trawl net (a, n=190) and gillnet (b, n=92) during dry and wet season. The box and whisker plots with the central line in each box represents the median; the circle symbol represents the mean; the bottom and top edges of the box represent the 25th and 75th percentiles (i.e. Q1 – Q3 or interquartile range, IQR). Whiskers extend to the minimum and maximum values within 1.5×IQR. Note that the y-axis scale of Figures 2a and 2b are not equal. A significant difference in marine debris caught in nets between the dry and wet season is noted, analysed separately for each study location using Kruskal-Wallis test with *P<0.05, **P<0.01, ***P <0.001 (detailed test statistics are available in Supplementary Table 2). **c,d:** The twelve most dominant categories of marine debris caught in each haul across seasons ranked by weight (c, n=282, % by weight) and item count (d, n=282, % by item count). Green, blue, and yellow shades of colour represent fishing-related debris, consumer plastic debris, and non-plastic debris, respectively.

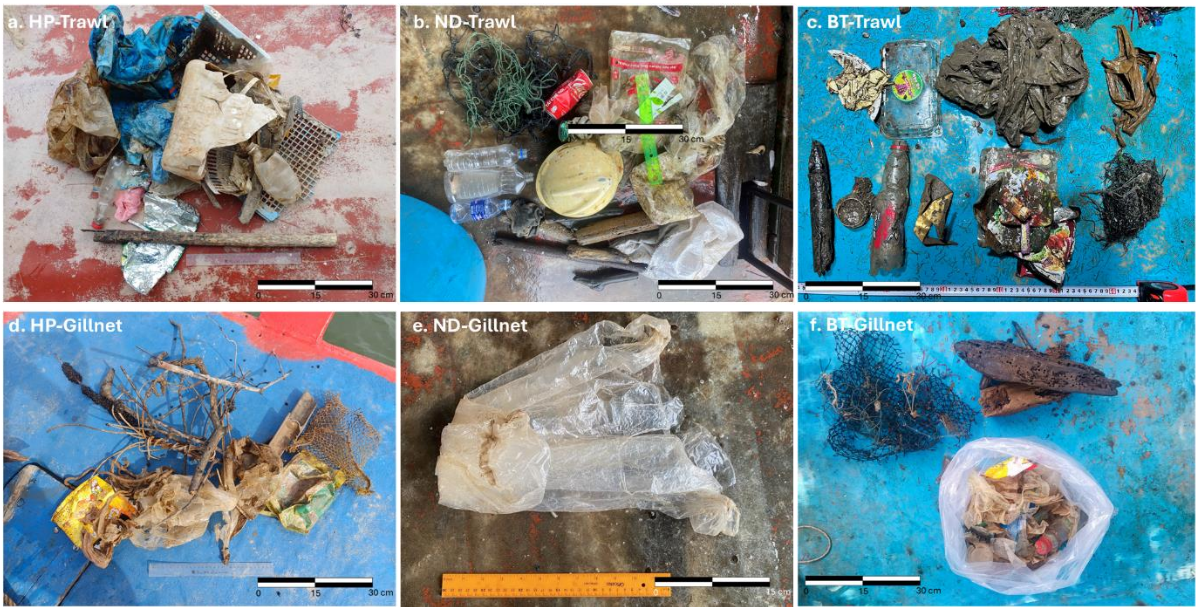

Figure 3: Visual examples of marine debris caught by nearshore fishing vessels in Hai Phong (HP), Nam Dinh (ND), and Ben Tre (BT). The images illustrate debris recovered from a single haul by trawlers (a–c) and gillnetters (d–f) during the waste audit; corresponding quantitative data are presented in Figure 2.

Fishers' perceptions of potential interventions

Participating fishers were aware of the potential negative impacts of marine plastic debris and

abandoned, lost, or otherwise discarded fishing gear (ALDFG) on the environment, fish stocks, and their livelihoods (See Supplementary Table 7). Despite this awareness, 57% of fishers felt unable to take action to reduce the impact of marine plastics. The majority (77%) continued to discard marine debris caught in their nets back into the ocean, while 18% retained only valuable items to sell or brought back large objects to prevent future encounters. This mindset was encapsulated in a common sentiment:

"*I throw it back to the ocean, why should I bring it back. If I bring back but other people still throw the trash out, it will waste my efforts*" (See Supplementary Table 7).

The majority of fishers were interested in participating in a "fishing for litter" programme, whereby they would be compensated for collecting and bringing marine litter back to shore (n=196: 56% strongly agree and 23% agree). In general, fishers indicated that they would need to be paid a median buyback price of 0.16 USD kg^{-1} (0.08 - 0.23 USD kg^{-1}) of marine plastic debris, with no significant difference among gill net or trawl fishers (H1, 119=0.34, p=0.6), although fishers in Hai Phong requested a higher marine litter buy-back price of 0.25 USD kg^{-1} of marine plastic debris (H1, 119=19.9, p<0.001). A fisher in Hai Phong noted when asked about monetary incentive: "*The buying price for marine plastic must* [be] *higher than the current price on land, otherwise no one will bring back…just throw it back to the sea to continue another haul*". (See Supplementary Table 7).

DISCUSSION

Inequities in the impacts of plastic pollution

Our findings demonstrate the substantial negative impact of marine plastic on the income, livelihoods and well-being of nearshore trawl and gillnet fishers, with pronounced regional inequities.

The median annual economic loss from marine plastic debris per nearshore fishing vessel was estimated at USD 3421 (comprising direct costs of USD 594 and lost opportunity costs of USD 2816). This finding exceeds previously reported economic losses in central Viet Nam (1,364 USD $year^{-1}$[28]), Ecuador (569 USD $year^{-1}$), and Peru (669 USD $year^{-1}$)[29]. However, it is lower than estimates from higher-income countries, such as the Azores in Portugal (3502 USD $year^{-1}$)[30] and Scotland (20-23,000 USD $year^{-1}$)[31]. Our higher estimates relative to comparable regions in Viet Nam importantly reflect the inclusion of lost opportunity costs in our approach, which were omitted in previous studies. Taken on their own, our estimates of direct costs are consistent with earlier findings (Supplementary Table 8).

Although the economic loss incurred for gillnet and trawl vessels was not significantly different ($H_{1,199}$ = 1.48, p = 0.40), gillnet fishers reported encountering less marine debris in their nets (median 7 kg

haul^{-1}) than trawlers (15 kg haul^{-1}), yet spent 35% more labour time disentangling debris from their gillnets, which resulted in 48% higher total economic loss as compared to trawlers.

There was a considerable inequity in the cost burden (10% and 18% respectively) between fishing vessels in Hai Phong and Nam Dinh in the Red River Delta, compared to vessels in Ben Tre in the Mekong Delta (**Figure *1***d). Land-based marine plastics were mainly confined to the coastal zone [13,32] and concentrated at outlets of major river systems such as the Mekong River in Ben Tre[6]. Hence, coastal fishing communities in these regions bear the greatest cost burden compared to offshore fishers. Although the total absolute cost of plastic litter to fishing vessels is not significantly different between our case-study regions, the relative cost in terms of percentage of revenue loss was significantly higher for fishers in the Mekong Ben Tre province (**Figure *1***c,d). This disparity is likely a result of nearshore fishers in Ben Tre that had significantly lower annual revenue (711 USD vessel^{-1}, $H_{2,\ 198}$= 23.33, P<0.0001) and lower fishing yields (50 kg trip^{-1}, $H_{2,\ 192}$=41.07, P<0.0001) than fishers in in the Red River Nam Dinh and Hai Phong provinces, making them more vulnerable to the additional economic impacts of marine plastic pollution.

Global inequalities in plastic pollution are known to occur, such that lower income countries experience a 10.6 times higher cost from plastic pollution than higher income countries[33,34]. Income level is a key determinant of vulnerability to plastic pollution[35], as lower-income coastal communities often have poorly developed waste management infrastructure, fewer alternatives for livelihoods, limited capacity to avoid or mitigate pollution impacts, and greater dependence on degraded marine ecosystems[33]. Plastic waste exacerbates inequality, with coastal and marginalised communities bearing disproportionate social, environmental, and health harms across all stages of the plastic lifecycle, despite contributing least to this problem and having limited influence over decision-making[36].

The regional differences in this study are linked to a number of demographic factors (Supplementary Tables 3 and 6). Smaller-scale fishers in Ben Tre were the most economically vulnerable to the impacts of plastic pollution because they typically had older vessels with smaller, less fuel-efficient engines, and had lower catch efficiency compared to fishers from the Red River Delta front [37–39]. Furthermore, nearshore fishers in Ben Tre operated at the outlet of two of the world's most plastic-polluted rivers, the Mekong River and Saigon-Dong Nai river systems[6]. Plastic leakage into the coastal and nearshore environment in Ben Tre province is three times higher than in the Red River, Hai Phong and Nam Dinh in the north of Viet Nam[40] (See Supplementary Table 6). This is reflected in our waste audit data, which showed that significantly more plastic debris was caught in the fishing nets of fishers in Ben Tre compared to fishers in Nam Dinh (Kruskal-Wallis, $H_{2,283}$=15.92, P<0.001). Ben Tre fishers have limited options to move to alternative fishing grounds to avoid plastic pollution, as fuel accounted for 64% of

their operational costs. This was underlined by 53% of the interviewed fishers (n=68) who considered that they cannot do anything to alleviate the impact of marine debris;

"*I have to accept it, what else can I do?*"

Our study also revealed that marine debris results in serious accidents, especially when crew members must untangle fishing nets and ropes from propellers. Although marine plastic-related accidents are rare and not all fishers directly witnessed the incidents (making it difficult to verify whether the entangled ropes originated from their own fishing vessels or from ALDFG), the accidents reported emphasise the potential risks to nearshore fishers' health and safety. Reports of accidents led to crew injuries, casualties, or man-overboard situations caused by marine debris were previously reported in other countries[19,41]. Thus, plastic pollution in the coastal ocean has cumulative impacts on nearshore fishing communities, thereby contributing to the lifetime cost of marine plastic pollution[16]. Marine plastic also compounds other challenges confronting these fishers, such as the impacts of extreme weather events, reductions in biodiversity, depletion of fish stocks, and conflicts over-fishing zones[42]. These interconnected issues intensify the pressure on vulnerable communities, making their coastal livelihoods increasingly precarious as river delta economies continue to grow and exacerbate the plastic waste problem.

Lack of comparable data among socioeconomic impact assessment studies

Current research on the socioeconomic impacts of marine plastic in Viet Nam and elsewhere is scarce and lacks standardized data collection approaches[30]. Previous studies often only accounted for direct costs (i.e., gear damage, repair costs, time spent sorting catch from plastic) and did not include lost opportunity cost[30] which is clearly a substantial component of total cost (i.e. potential revenue from time lost that could otherwise be spent fishing, reduction in catch due to gear interference). Although fishers are often acutely aware of these opportunity losses, they rarely quantify them in monetary terms. For example, one fisher noted in an interview: "*We have to catch two tonnes a day before going home* (to have profit). *Before, 6–7 hauls were enough for two tons; now it takes 20–25 hauls*". This illustrates how the combination of over-exploited fish stocks and plastic pollution increases the effort required to achieve the same catch, translating into substantial but frequently unaccounted economic losses. Incorporating these factors is critical, as their omission can lead to systematic underestimation of the true economic impacts of plastic pollution on small-scale fisheries and limit the relevance of cost estimates for management and policy decisions.

The direct costs estimated in this study are between 0.6 to 3.6 times higher than previously reported values for Viet Nam[27] which could be associated with differences in local demographics and environmental context. Direct comparison with other empirical and large-scale modelling estimates

from different locations worldwide remains challenging due to the absence of standardized methods and protocols, as well as differences in the scope and types of cost components and impact factors quantified across studies (See Supplementary Table 8 for detailed comparison).[30]

The lack of use of consistent reporting units and economic estimation methods has been identified as one of the challenges in comparing the impacts of plastic pollution and ALDFG[43]. Detailed guidelines on quantifying marine debris and its impacts are therefore essential for quantifying pollution impacts[44].

Using local knowledge to identify and mitigate plastic pollution hotspots

Identifying spatiotemporal hotspots of marine plastic debris is essential for guiding targeted mitigation and cleanup efforts. While numerical models can simulate large-scale and long-term plastic transport via ocean currents[45]. They require validation through field observations, which are often costly and spatially or temporally limited. LEK from fishers, who spend extensive time at sea and rely on understanding marine dynamics for their livelihoods, offers a valuable resource to bridge these knowledge gaps and enhance hotspot detection.

Results from the waste audit (

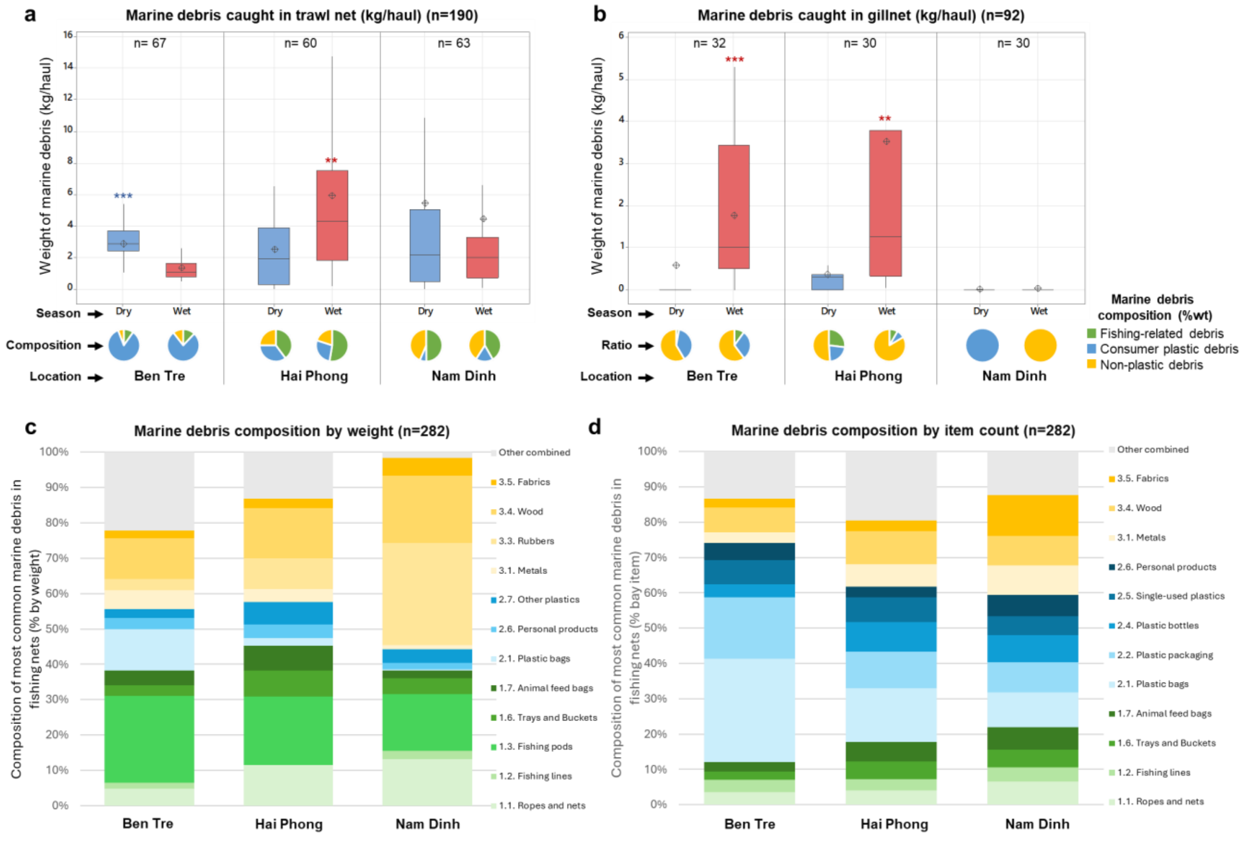

Figure ***2***) and surveys with fishing communities (See Supplementary Table 5) were aligned, indicating that local knowledge was a valuable and reliable source of information for identifying different plastic pollution hotspots, seasonality, items and potential polluters.

The scientific reliability and accuracy of fishers' knowledge of the distribution of plastic is also reflected by the fact that the reported hotspots (See Supplementary Table 5) agreed with the predictions of previous numerical simulations of the transport of macroplastics exported from Ba Lat River of the Red River system[13]. Local knowledge held by fishers has been widely used in conservation and informed context-specific problems that are often missed by top-down assessments [46]. Empirical evidence from Viet Nam[28], Cameroon[47], and European countries[48,49] shows that incorporating fishers' knowledge has supported the co-design of targeted ALDFG prevention, retrieval schemes, and improved port waste management, leading to more practical and locally accepted interventions.

The local knowledge of fishers complements scientific data and, when combined, should be used to inform co-management for locating and tackling marine plastic debris[28,46]. On the other hand, once corroborated with local knowledge, field sampling data and plastics transport modelling may inform best-practice and optimal locations for beach and ocean waste cleanup operations. Previous numerical modelling [13] suggested that during the dry season, plastics were transported back towards the shore, creating a potential window of opportunity to remove those plastics from the beach at a much lower cost than from the sea surface.

Solutions to mitigate the impacts of plastic pollution on nearshore fishing communities

Fishers' preferred solution to actively engage in the marine pollution problem was an incentivised "fishing for litter" programme, whereby fishers would be compensated for collecting marine plastic waste. Our data show that enrolment in such a programme should offer a median buyback price of only 0.16 USD kg^{-1} of marine plastic debris, or "*equivalent to the market price of trash fish*", as fishers often mentioned during the interviews.

Using waste audit data, each fishing boat is estimated to catch a median of 0.60 kg (0.01–2.55 kg) of marine plastic per haul, with a median of 630 hauls $year^{-1}$. A fishing-for-litter programme would therefore need to pay each participating vessel approximately 60 USD $year^{-1}$ to bring all collected marine plastic ashore (equivalent to 378 kg of plastic per vessel annually). The additional cost of treating the returned plastic would be around 20 USD $vessel^{-1}$ $year^{-1}$, assuming a waste treatment rate of 54 USD ton^{-1} in 2025 at an annual inflation rate of 5%[50]).

Such incentivised programs have been piloted in Viet Nam [51] and European countries [48,49] with initial success. However, these pilots often failed to consider socioeconomic inequalities when selecting

implementation sites. Our study provides an important recommendation that any ‘fishing for litter’ initiative should be prioritised at “plastic cost hotspots” to alleviate the economic impact of marine plastic on the most vulnerable fishing communities and to optimise the timing and location of coastal and marine debris cleanup initiatives.

Future research should relate data on highly plastic polluted rivers[6] to the location of low-income, fisheries-dependent areas to identify priority locations[35] where marine plastic collection incentive programmes could be implemented. Based on a rapid, exploratory analysis of currently available datasets, we identified indicative priority locations for Viet Nam and globally in Supplementary Table 10. Two branches of the Mekong River system (namely Hau River and Vam Co River) are among the top 10 priority candidate rivers for Viet Nam. Rivers in the Philippines and India are potential candidates due to their high plastic output and occurrence of lower income communities[28]. Sustainable solutions must also consider educational campaigns that explain the real cost of plastic litter, the benefits of recycling and other systemic changes. Such systems-change approaches should enhance both the “hardware” (physical/environmental changes) by increasing solid waste collection rates from upstream to downstream, and the “software” (societal changes) by altering social norms in solid waste management through legal and societal reinforcement.[52,53]

CONCLUSION

This study highlights the economic and social burdens faced by small-scale nearshore fishing communities in delta front regions of the Mekong and the Red River in Viet Nam. Based on reports from 199 fishers and related stakeholders, the average cost on nearshore fishers is estimated to be 10%, 10.6%, and 18% of the annual revenue of each fishing vessel in the Red River Hai Phong City and Nam Dinh, and the Mekong River Ben Tre provinces, respectively. The study identified inequalities in the economic impacts of marine plastic debris. Nearshore fishers operating close to the Mekong estuaries faced the strongest environmental and social stressors and were likely to experience greater impacts compared to Red River settings. Furthermore, fishers reported higher health and safety risks, particularly during the monsoon season, due to plastic entanglement incidents. Local fishers demonstrated a strong understanding of where and when hotspots of marine plastics may occur and proposed solutions in which they are willing to participate if centrally managed. These findings are corroborated by data from the validation survey with additional groups of fishers and by field-based waste audits on fishing vessels. Fishers emphasised the need for incentive-based marine plastic collection programmes, prioritised at socioeconomic hotspots where plastic pollution most severely affects low-income coastal communities. The results from this study provide components of a framework for understanding economic impact hotspots and co-designing effective solutions with

community participation to tackle marine plastic pollution in coastal areas worldwide.

METHODS

Location and scope of study

This study focused on small-scale nearshore fishing communities in three regions: Hai Phong City (HP), Nam Dinh Province (ND), and Ben Tre Province (BT) (Figure 4). These sites were selected for their contrasting geographical and socioeconomic characteristics (Supplementary Table 6). HP represents a coastal and island dominated area with high income, important fishing landing sites, maritime shipping industries, and tourism destinations. ND and HP are located at the outlets of the Red River in the north. BT is adjacent to the Mekong River in the south of Viet Nam and is characterised by small-scale fisheries, aquaculture, agriculture, and other coastal businesses. Previous studies show that these two

rivers are among the top rivers in the world in terms of discharge of plastic waste into the coastline and ocean [6,52].

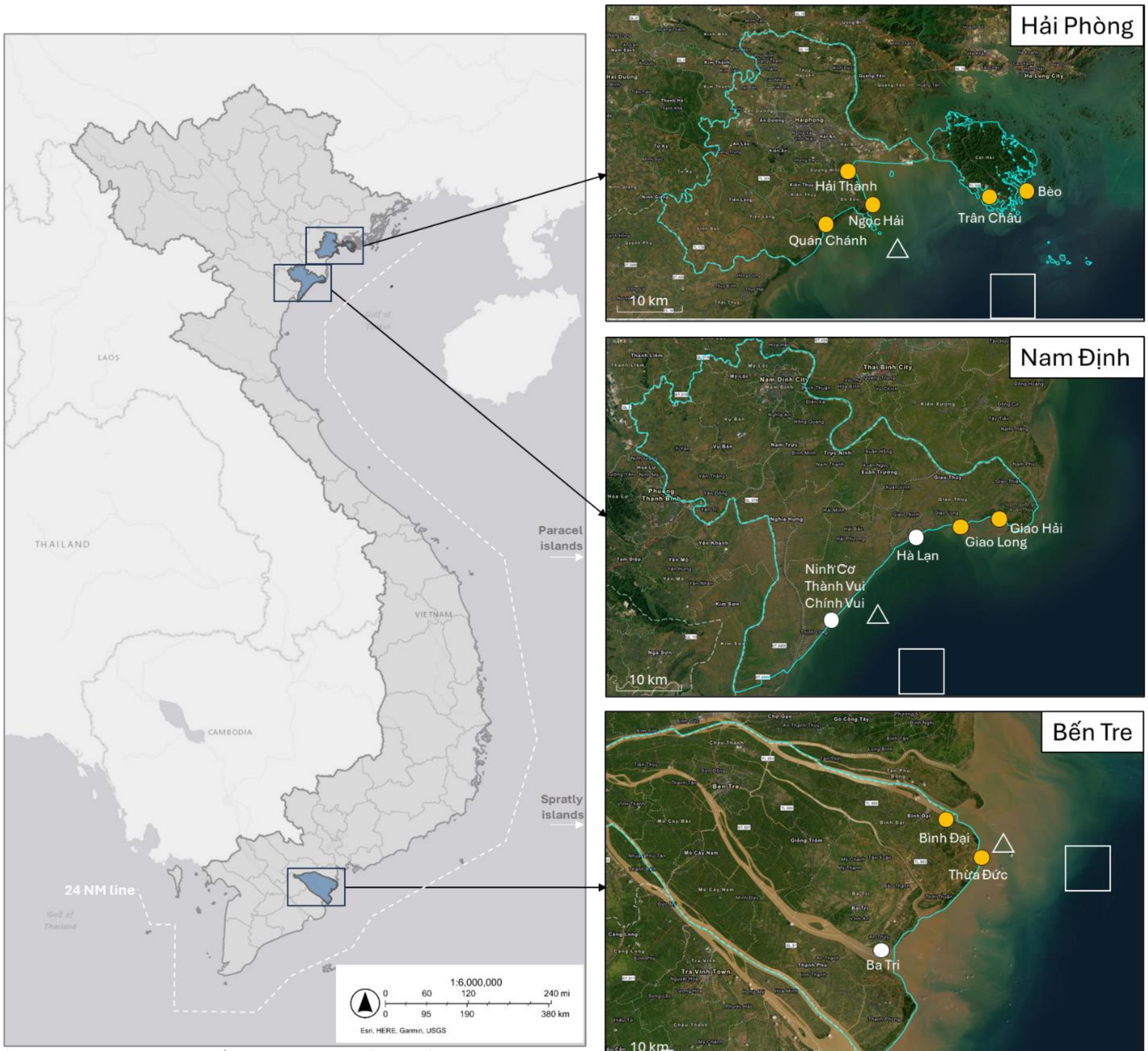

Image sources: www.arcgis.com; www.earth.google.com

Figure 4: Locations of the study areas in Hai Phong, Nam Dinh, and Ben Tre. Yellow dots are the locations of fishing ports and communities involved in the socio-economic impacts survey (n = 199 fishers); white dots are additional fishing port locations included in the verification survey (n = 94 fishers). Triangles show the locations of waste audits on gillnetters (n = 92 observations); rectangles indicate the locations of waste audits on trawlers (n = 190 observations).

In this study, the term "nearshore" refers to both coastal and inshore fishing zones, as defined by Vietnamese fisheries law, to include the area from the shore out to 24 nautical miles. Previous research indicates that globally up to 77% of buoyant ocean plastics are concentrated in the coastal zone[32]. We

focused on small-scale fishing vessels owned and operated by local families using trawls and gillnets in nearshore waters that are disproportionately impacted by plastic debris [25,28,53].

Data collection

The study was conducted following mixed methods[15] that were implemented in a consistent sequence at each field site (Figure 5). Participants were selected through a combination of snowball sampling, initiated with key informants (e.g., governmental officers from the Department of Fisheries or leaders of fishing groups within the commune), and random sampling with fishers at different ports (Figure 4), allowing us to collect data from 308 individuals. A total of fifteen semi-structured interviews with fisheries governmental officers (n=9) and fishing port managers (n=6), and three focus group discussions (FGDs) (n=8 participants each, who also participated in the socio-economic impact survey) with fishers were conducted across the three field sites. The socio-economic impact survey consisted of 199 structured interviews with nearshore fishers (see demographics in Supplementary Table 3) to quantify the economic impacts of marine plastic debris on their livelihoods. A verification survey using structured interviews was conducted with fishers who had not participated in the previous socio-economic impact survey (n=94) to assess the extent of agreement or disagreement that fishers had with the research findings. A waste audit survey (n=282 observations) was conducted in the same study sites with the socio-economic impact interviews to corroborate the quantification of marine plastic debris encountered in fishing nets. Detailed data collection procedure, calculation methods, and data limitations are available in Supplementary Information 1.

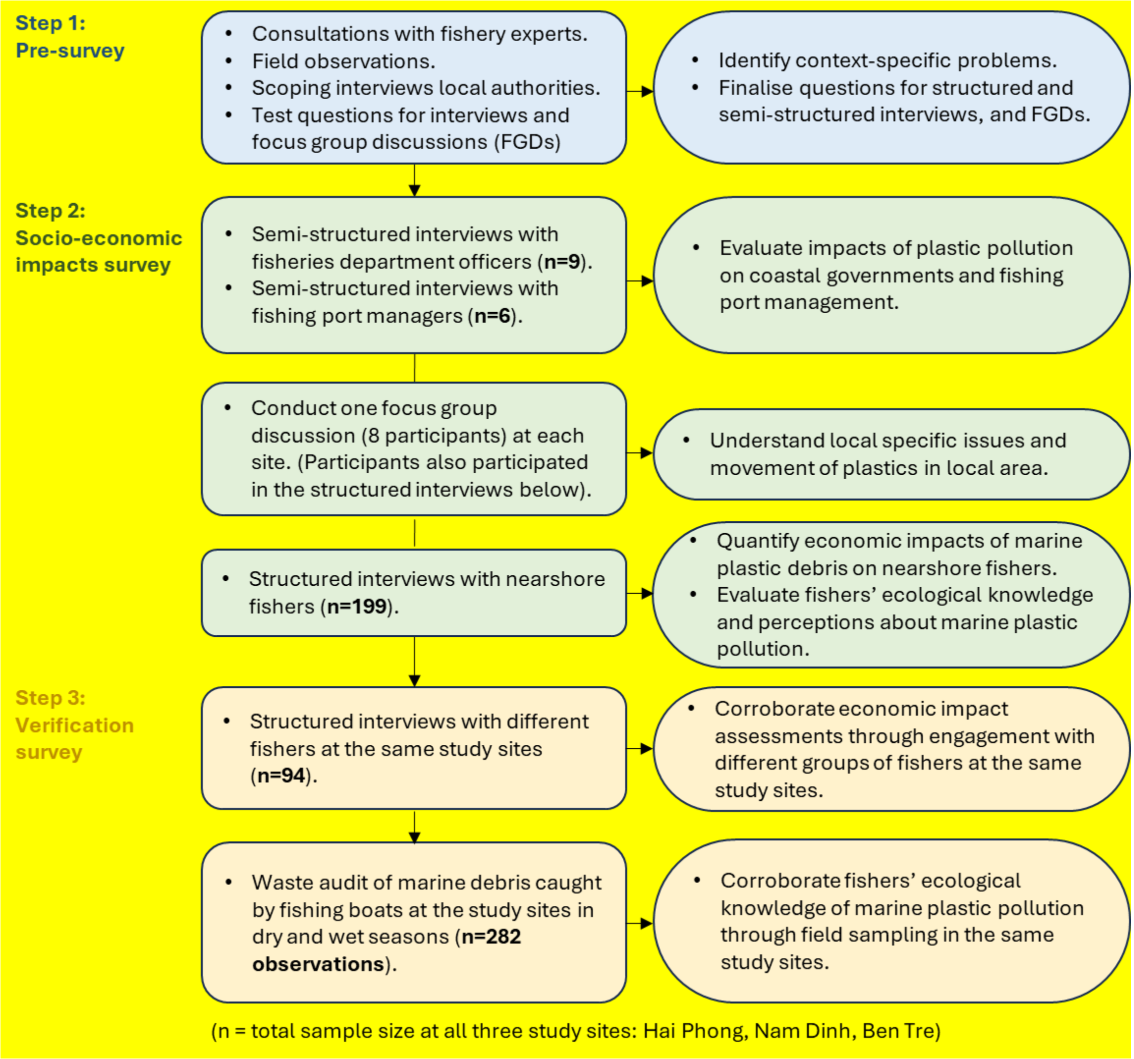

Figure 5: Data collection procedure of the mixed-method design followed in each study location.

Cost estimation

Using the data from the first survey with 199 fishers, the economic costs were calculated with the following formula:

Total Cost (TC) = Direct cost (DC=DC1+DC2+DC3) + Lost opportunity cost (LC=LC1+LC2)

Where:

DC1 = Labour cost of crew members' combined time spent separating plastic debris from fishing gear and the catch

DC2 = Labour cost of crew member's combined time spent untangling plastic debris from propellers, cooling water inlets, etc.

DC3 = Cost of replacing and fixing damaged gears from encountering plastic debris and/or towing the vessel to port for repairing if the vessel was disabled due to plastic debris

LC1 = Loss of revenue from reducing fishing efficiency due to plastic debris by tearing nets, blocking nets, and causing nets to float, among other issues.

LC2 = Loss of revenue from the total time lost when the fishing vessel could have been fishing

The costs incurred from encounters with marine plastic debris were calculated individually for each vessel, based on the fishers' responses and estimates. Each of the five components to the total cost was calculated based on multiple contributing factors, including: type of plastic-related incidents (non-exhaustive list includes: stuck in fishing nets, mix in the catch, entangle in propeller, block water inlet, damage fishing net, etc.), frequency of each incident (incidents per year), time loss (hours per incident), number of people involved (people per incident), labour cost (USD per working hour), repair cost (USD per incident), estimated reduction in catch efficiency (% reduction), revenue per unit effort (USD per hour fishing), and estimated plastic content in mixed marine debris (% by weight). All interviews were conducted in Vietnamese, and the cost was calculated in local currency before being converted to USD. Details of the cost calculation are available in Supplementary Information 1.

The direct cost represents expenses that fishers could recall in detail, making them more reliable. The lost opportunity cost uses fishers' estimation of catch efficiency reduction due to plastic debris and loss of revenue per unit time lost, and therefore is subject to a higher degree of uncertainty and fluctuations [30,37].

Waste Audit

A waste audit was conducted on board fishing vessels by observers following the method adapted from GESAMP, 2019[54]. A total of six small-scale fishing vessels (one trawler and one gillnetter for each of the three study areas) were contracted to participate in the study on the spatiotemporal distribution of marine litter in nearshore waters. In total, we collected 282 observations (190 hauls from trawlers and 92 hauls from gillnetters), split between three study areas and two seasons (Dry season: 17th April to 30th May, 2024; Wet season: 1st August to 30th September, 2024). The same vessel and gear were used in both sampling periods to compare seasonal variability. However, the fishing gear at the three locations differed slightly. The bottom trawlers had nets with a mesh size of 25–35 mm and operated at a maximum water depth of 10–36 m, while the gillnetters used nets with a mesh size of 45–55 mm and were deployed at a water depth of 5–10 m from the water surface.

Data analyses

Anonymized interview responses were entered, coded and screened for missing data and invalid answers (i.e. blank and "I don't know" answers). Audio recordings of the semi-structured interviews and FGDs were transcribed, translated, and coded in NVivo 14 (Version 14.23.2 (46)). Data analysis was

conducted using IBM SPSS Statistics software (Version 29.0.1.0 (171)). The estimated costs and other data reported by interviewers were non-normally distributed (Shapiro–Wilk test, $p < 0.001$). Therefore, we reported all values as medians (Q1 - Q3). Significant differences were tested using Kruskal–Wallis test with the test statistic H-value (degree of freedom (df), total samples (n)), presented as $H_{df, n}$ and associated P-values. Detailed results of data analyses are available in supplementary tables in Supplementary Information 2.

OTHER SECTIONS

CRediT authorship contribution statement

Duc Nguyen: Methodology, Data curation, Visualisation, Investigation, Writing.

Phan Phuong Thanh: Investigation, Data curation, Writing – review & editing

Heidi Burdett: Methodology, Funding acquisition, Writing – review & editing

Michel Kaiser: Project lead, funding acquisition, conceptualisation, Writing – review & editing

Thomas Wagner: Conceptualisation**,** Writing – review & editing

Ryan Pereira: Writing – review & editing

Inna Yavana-Toraman: Methodology, Writing – review & editing

Zhiling Liao: Writing – review & editing

Qingping Zou: Writing – review & editing

Trinh Quang Tu, Le Trung Dung, Le Thi Thu Huong: Investigation

Data availability

The data used in this study are included in the article, supplementary information and are publicly available through PURE at https://doi.org/10.17861/47e741f5-3810-4f40-9b03-fba8cf825913 . Any additional information is available upon request to the corresponding author.

Ethics declaration

Data in this study was collected and protected following the Equality and Privacy Impact Assessment and Data Management Plan approved for the project "Sources, Sinks and Solutions for Impacts of Plastics on Coastal Communities in Viet Nam" (3SIP2C) (NE/V006088/1). The research was subject to full ethics approval through the Heriot-Watt University ethics committee: approval number 2023-3366-7400.

Acknowledgement

This research was funded by the UKRI-NERC through Global Challenges Research Fund (GCRF) programme (NE/V006088/1). We thank all the fishers, informants, and other participants for contributing their time and knowledge that made this study. We thank all team members of the project 3SIP2C (https://lessplasticvietnam.com).

REFERENCES

1. **Bergmann, M. *et al.* Plastic pollution in the Arctic. *Nat. Rev. Earth Environ.* 3, 323–337 (2022).**
2. **Abel, S. M., Wu, F., Primpke, S., Gerdts, G. & Brandt, A. Journey to the deep: plastic pollution in the hadal of deep-sea trenches. *Environmental Pollution* 333, 122078 (2023).**
3. **Omeyer, L. C. M. *et al.* Interactions between marine megafauna and plastic pollution in Southeast Asia. *Science of the Total Environment* 874, 162502 (2023).**
4. **Ugwu, K., Herrera, A. & Gómez, M. Microplastics in marine biota: A review. *Mar. Pollut. Bull.* 169, (2021).**
5. **Liu, S. *et al.* Detection of various microplastics in placentas, meconium, infant feces, breastmilk and infant formula: A pilot prospective study. *Science of the Total Environment* 854, 158699 (2023).**
6. **Meijer, L. J. J., van Emmerik, T., van der Ent, R., Schmidt, C. & Lebreton, L. More than 1000 rivers account for 80% of global riverine plastic emissions into the ocean. *Sci. Adv.* 7, (2021).**
7. **Stubbins, A., Law, K. L., Muñoz, S. E., Bianchi, T. S. & Zhu, L. Plastics in the Earth system. *Science (1979).* 373, 51–55 (2021).**
8. **Velis, C., Cottom, J. & Cook, E. A local-to-global emissions inventory of macroplastic pollution. *Nature* 633, (2023).**
9. **Lebreton, L. C. M. *et al.* River plastic emissions to the world's oceans. *Nat. Commun.* 8, 1–10 (2017).**
10. **van Emmerik, T., Mellink, Y., Hauk, R., Waldschläger, K. & Schreyers, L. Rivers as Plastic Reservoirs. *Frontiers in Water* 3, 1–8 (2022).**
11. **van Emmerik, T. H. M. *et al.* River plastic transport and deposition amplified by extreme flood. *Nature Water* 1, 514–522 (2023).**

12. **Morales-Caselles, C. *et al.* An inshore–offshore sorting system revealed from global classification of ocean litter. *Nat. Sustain.* 4, 484–493 (2021).**

13. **Liao, Z., Zou, Q., Duy, V., Pan, Z. & Kaiser, M. J. Seasonal change in fate and transport of plastics from Red River to the coast of Vietnam. *Mar. Pollut. Bull.* 208, 116923 (2024).**

14. **Wen, Z., Xie, Y., Chen, M. & Dinga, C. D. China's plastic import ban increases prospects of environmental impact mitigation of plastic waste trade flow worldwide. *Nat. Commun.* 12, 1–9 (2021).**

15. **Ramírez, D. M., Sachs, A. L. & Ekenga, C. C. Qualitative and mixed methods: informing and enhancing exposure science. *J. Expo. Sci. Environ. Epidemiol.* https://doi.org/10.1038/s41370-024-00707-5 (2024) doi:10.1038/s41370-024-00707-5.**

16. **WWF. *Plastics: The Costs To Society, the Environment and the Economy*. www.panda.org (2021).**

17. **Deloitte. *The Price Tag of Plastic Pollution - An Economic Assessment of River Plastic*. *Deloitte* https://www2.deloitte.com/content/dam/Deloitte/nl/Documents/strategy-analytics-and-ma/deloitte-nl-strategy-analytics-and-ma-the-price-tag-of-plastic-pollution.pdf (2019).**

18. **Richardson, K., Wilcox, C., Vince, J. & Hardesty, B. D. Challenges and misperceptions around global fishing gear loss estimates. *Mar. Policy* 129, 104522 (2021).**

19. **Aretoulaki, E., Ponis, S., Plakas, G. & Agalianos, K. Marine Plastic Littering: a Review of Socio Economic Impacts. *J. Sustain. Sci. Manag.* 16, 277–301 (2021).**

20. **Lebreton, L. *et al.* Industrialised fishing nations largely contribute to floating plastic pollution in the North Pacific subtropical gyre. *Sci. Rep.* 12, 1–11 (2022).**

21. **Richardson, K., Hardesty, B. D. & Wilcox, C. Estimates of fishing gear loss rates at a global scale: A literature review and meta-analysis. *Fish and Fisheries* 20, 1218–1231 (2019).**

22. **Basurto, X. *et al.* Illuminating the multidimensional contributions of small-scale fisheries. *Nature* 637, 875–884 (2025).**

23. **VASEP. Fishery profile. https://seafood.vasep.com.vn/why-buy-seafood/fishery-profile (2025).**

24. **MARD. *REPORT ON PLANNING FOR THE PROTECTION AND UTILISATION OF FISHERIES RESOURCES FOR THE PERIOD 2021-2030, WITH A VISION TOWARD 2050 (In Vietnamese)*. (2024).**

25. **WWF-Viet Nam. *Khảo Sát Quốc Gia về Sự Đóng Góp Của Rác Thải Nhựa Từ Hoạt Động Khai Thác và Nuôi Trồng Thủy Sản Đến Rác Thải Nhựa Đại Dương*. (2020).**

26. **Veettil, B. K., Hua, N. T. A., Van, D. D. & Quang, N. X. Coastal and marine plastic pollution in Vietnam: Problems and the way out. *Estuar. Coast. Shelf Sci.* 292, 108472 (2023).**

27. **Guerrato, N. R. & Gonçalves, L. R. Netting the problem: a comprehensive analysis of marine litter on artisanal fishers. *Frontiers in Ocean Sustainability* 2, (2025).**

28. **Xuan, B. B., Ngoc, Q. T. K. & Börger, T. Fisher preferences for marine litter interventions in Vietnam. *Ecological Economics* 200, (2022).**

29. **Llerena, P. D. *et al.* Assessing economic losses in artisanal fisheries from marine plastic pollution in coastal Ecuador and Peru. *Mar. Policy* 173, (2025).**

30. **Rodríguez, Y., Ressurreição, A. & Pham, C. K. Socio-economic impacts of marine litter for remote oceanic islands: The case of the Azores. *Mar. Pollut. Bull.* 160, 111631 (2020).**

31. **KIMO. *Economic Impacts of Marine Litters*. http://www.vliz.be/imis/imis.php?refid=205614 (2010).**

32. **Onink, V., Jongedijk, C. E., Hoffman, M. J., van Sebille, E. & Laufkötter, C. Global simulations of marine plastic transport show plastic trapping in coastal zones. *Environmental Research Letters* 16, (2021).**

33. **WWF. *WHO PAYS FOR PLASTIC POLLUTION ? ENABLING GLOBAL EQUITY IN THE PLASTIC VALUE CHAIN*. (2023).**

34. **McIlgorm, A., Raubenheimer, K., McIlgorm, D. E. & Nichols, R. The cost of marine litter damage to the global marine economy: Insights from the Asia-Pacific into prevention and the cost of inaction. *Mar. Pollut. Bull.* 174, (2022).**

35. **Hardesty, B. D. *et al.* Socioeconomics effects on global hotspots of common debris items on land and the seafloor. *Global Environmental Change* 71, (2021).**

36. **UNEP. *Neglected: Environmental Justice Impacts of Marine Litter and Plastic Pollution*. (2021).**

37. **Ngoc, Q. T. K., Xuan, B. B., Duy, N. N. & Börger, T. The Impact of Marine Litter on Production Risk and Technical Efficiency in the Trawl Fisheries of Vietnam. *Marine Resource Economics* 38, 65–84 (2023).**

38. Nguyen, Q., Pascoe, S. & Coglan, L. Implications of regional economic conditions on the distribution of technical efficiency: Examples from coastal trawl vessels in Vietnam. *Mar. Policy* 102, 51–60 (2019).

39. Pomeroy, R. *et al.* Fish wars: Conflict and collaboration in fisheries management in Southeast Asia. *Mar. Policy* 31, 645–656 (2007).

40. WWF-Viet Nam. *Report on Plastic Waste Generation in 2022*. (Thanh Nien Publishing House, Hanoi, 2023).

41. Kim, B., Lee, H. & Kim, T. Possibility analysis of complex marine accidents caused by large marine plastic litter in the Republic of Korea. *Mar. Policy* 152, 105591 (2023).

42. Beaumont, N. J. *et al.* Global ecological, social and economic impacts of marine plastic. *Mar. Pollut. Bull.* 142, 189–195 (2019).

43. Mengo, E. *et al.* Fishers' views and experiences on abandoned, lost or otherwise discarded fishing gear and end-of-life gear in England and France. *Mar. Pollut. Bull.* 194, 115372 (2023).

44. Walker, T. R., McGuinty, E. & Hickman, D. Marine debris database development using international best practices: A case study in Vietnam. *Mar. Pollut. Bull.* 173, 112948 (2021).

45. Liao, Z., Zou, Q., Vinh, V. D., Pan, Z. & Kaiser, M. J. Seasonal change in fate and transport of plastics from Red River to the coast of Vietnam. *Mar. Pollut. Bull.* 208, (2024).

46. Truchet, D. M. *et al.* Fishers' ecological knowledge about marine pollution: What can fek contribute to ecological and conservation studies of a southwestern atlantic estuary? *Herpetologica* 75, 584–606 (2019).

47. Beseng, M. Managing abandoned, lost, or otherwise discarded fishing gear in Cameroon's marine small-scale fisheries: Perspective of local ecological knowledge. *Mar. Policy* 185, (2026).

48. Forleo, M. B. & Romagnoli, L. Fishing for litter for the reduction of marine plastic debris: What benefits and costs do Italians perceive? *Mar. Pollut. Bull.* 192, 115018 (2023).

49. Wyles, K. J., Pahl, S., Carroll, L. & Thompson, R. C. An evaluation of the Fishing For Litter (FFL) scheme in the UK in terms of attitudes, behavior, barriers and opportunities. *Mar. Pollut. Bull.* 144, 48–60 (2019).

50. The World Bank. Solid and Industrial Hazardous Waste Management Assessment - Options and action area to implement the national strategy. 107 (2018).

51. **Quertamp, F., Cương, N. T., Long, N., Viên, H. & Tố, N. C. *RESULTS OF THE PILOT PROJECT "FISHING FOR LITTER"*. (2022).**

52. **Apete, L., Martin, O. V. & Iacovidou, E. Fishing plastic waste: Knowns and known unknowns. *Mar. Pollut. Bull.* 205, 116530 (2024).**

53. **Pomeroy, R., Thi Nguyen, K. A. & Thong, H. X. Small-scale marine fisheries policy in Vietnam. *Mar. Policy* 33, 419–428 (2009).**

54. **Strokal, M. *et al.* River export of macro- and microplastics to seas by sources worldwide. https://doi.org/10.1038/s41467-023-40501-9 (2023) doi:10.1038/s41467-023-40501-9.**

55. **Raes, L., Jain, A., Ba, T. N. & Savels, R. The economic impact of marine plastics, including ghost fishing, on fishing boats in Phước Tinh and Loc An, Ba Ria Vung Tau Province, Viet Nam. https://biblio.ugent.be/publication/8764101/file/8764102 (2022).**

56. **GESAMP. *Guidelines for the Monitoring and Assessment of Plastic Litter in the Ocean*. *GESAMP* vol. R99 http://www.gesamp.org/publications/guidelines-for-the-monitoring-and-assessment-of-plastic-litter-in-the-ocean (2019).**
